# Phase II Study Evaluating 2 Dosing Schedules of Oral Foretinib (GSK1363089), cMET/VEGFR2 Inhibitor, in Patients with Metastatic Gastric Cancer

**DOI:** 10.1371/journal.pone.0054014

**Published:** 2013-03-14

**Authors:** Manish A. Shah, Zev A. Wainberg, Daniel V. T. Catenacci, Howard S. Hochster, James Ford, Pamela Kunz, Fa-Chyi Lee, Howard Kallender, Fabiola Cecchi, Daniel C. Rabe, Harold Keer, Anne-Marie Martin, Yuan Liu, Robert Gagnon, Peter Bonate, Li Liu, Tona Gilmer, Donald P. Bottaro

**Affiliations:** 1 The Weill Cornell Medical College/New York–Presbyterian Hospital, New York, New York, United States of America; 2 University of California Los Angeles, Los Angeles, California, United States of America; 3 University of Chicago, Chicago, Illinois, United States of America; 4 Yale University, New Haven, Connecticut, United States of America; 5 Stanford University, Stanford, California, United States of America; 6 University of New Mexico, Albuquerque, New Mexico, United States of America; 7 GlaxoSmithKline, Collegeville, Pennsylvania, United States of America; 8 Urologic Oncology Branch, Center for Cancer Research, National Cancer Institute, Bethesda, Maryland, United States of America; 9 Exelixis, South San Francisco, California, United States of America; 10 GlaxoSmithKline, Research Triangle Park, North Carolina, United States of America; University Clinic of Navarra, Spain

## Abstract

**Purpose:**

The receptors for hepatocyte and vascular endothelial cell growth factors (MET and VEGFR2, respectively) are critical oncogenic mediators in gastric adenocarcinoma. The purpose is to examine the safety and efficacy of foretinib, an oral multikinase inhibitor targeting MET, RON, AXL, TIE-2, and VEGFR2 receptors, for the treatment of metastatic gastric adenocarcinoma.

**Patients and Methods:**

Foretinib safety and tolerability, and objective response rate (ORR) were evaluated in patients using intermittent (240 mg/day, for 5 days every 2 weeks) or daily (80 mg/day) dosing schedules. Thirty evaluable patients were required to achieve alpha = 0.10 and beta = 0.2 to test the alternative hypothesis that single-agent foretinib would result in an ORR of ≥25%. Up to 10 additional patients could be enrolled to ensure at least eight with *MET* amplification. Correlative studies included tumor *MET* amplification, MET signaling, pharmacokinetics and plasma biomarkers of foretinib activity.

**Results:**

From March 2007 until October 2009, 74 patients were enrolled; 74% male; median age, 61 years (range, 25–88); 93% had received prior therapy. Best response was stable disease (SD) in 10 (23%) patients receiving intermittent dosing and five (20%) receiving daily dosing; SD duration was 1.9–7.2 months (median 3.2 months). Of 67 patients with tumor samples, 3 had *MET* amplification, one of whom had SD. Treatment-related adverse events occurred in 91% of patients. Rates of hypertension (35% vs. 15%) and elevated aspartate aminotransferase (23% vs. 8%) were higher with intermittent dosing. In both patients with high baseline tumor phospho-MET (pMET), the pMET:total MET protein ratio decreased with foretinib treatment.

**Conclusion:**

These results indicate that few gastric carcinomas are driven solely by MET and VEGFR2, and underscore the diverse molecular oncogenesis of this disease. Despite evidence of MET inhibition by foretinib, single-agent foretinib lacked efficacy in unselected patients with metastatic gastric cancer.

**Trial Registration:**

ClinicalTrials.gov NCT00725712

## Introduction

Gastric cancer (GC) is the fourth most common cancer worldwide [1], [2], with an estimated 990,000 new cases and 730,000 deaths occurring annually [3], Despite its prevalence, drug development for GC has lagged behind the progress observed in other malignancies [4], with median survival of <1 year for advanced disease [5]. Recent success in targeting human epidermal growth factor receptor 2 (HER2) in GC [6] provides hope for similar success with other molecular targets.

The receptor tyrosine kinases (RTKs) MET and vascular endothelial growth factor receptor 2 (VEGFR2/KDR) are emerging therapeutic targets in gastric adenocarcinoma. MET, the receptor for hepatocyte growth factor (HGF), is a central mediator of tumor cell growth, survival and motility [7]. *MET* amplification has been demonstrated in 5–23% of primary gastric tumors [8]–[14] and is associated with poor prognosis [8], [9], [14]. In GC cell lines, *MET* amplification is associated with the presence of homogeneous staining regions, indicating targeted amplification and suggesting vulnerability to MET inhibition [15]. An activating *MET* mutation in GC has also been reported [16]. MET protein overexpression correlates with increased depth of tumor invasion and metastatic potential [17], [18]. VEGFR2 mediates endothelial cell migration, proliferation and survival [19], [20], and MET and VEGFR2 work in concert to promote neoangiogenesis [20]. RON, a MET-related RTK, was recently found to be highly expressed in 74% of GC tumors [14]. MET was highly expressed in 43% of RON-expressing tumors, and co-expression was predictive of worse overall survival (OS) than overexpression of RON alone [14].

Foretinib is an oral, small-molecule multikinase inhibitor that targets MET, RON, AXL, TIE-2 and VEGFR2 receptors with high *in vitro* affinity [21], [22]. Foretinib binds deep in the adenosine triphosphate pocket of its targets, resulting in conformational change and kinase inhibition [20], [21]. In preclinical studies, foretinib inhibited tumor cell proliferation, invasion and tumor angiogenesis [20], [21]. In Phase I evaluation, oral foretinib 240 mg daily for 5 days of each 2-week cycle (intermittent dosing) and 80 mg daily (continuous dosing) was well tolerated and showed preliminary evidence of anti-tumor activity in patients with solid tumors [23], [24]. Pharmacodynamic (PD) studies performed on serial tumor biopsy samples in three patients who received intermittent-dose foretinib also showed decreased AKT and ERK phosphorylation following foretinib dosing [23]. Data from one Phase II and one Phase I/II study showed evidence of tumor regression in patients with papillary renal carcinoma [25], [26], and hepatocellular carcinoma [27], respectively. Foretinib was generally well tolerated in these populations.

Based on published evidence of oncogenic MET and VEGFR2 signaling in GC, and MET pathway inhibition in Phase I foretinib evaluation, we examined the safety and efficacy of single-agent foretinib in the treatment of previously treated metastatic gastric adenocarcinoma. We additionally analyzed the relationship between pharmacokinetic (PK) and PD profiles and anti-tumor efficacy, and compared safety and efficacy for intermittent versus daily dosing regimens. As correlative objectives, we assessed *MET* amplification and MET targeting with foretinib in advanced GC.

## Patients and Methods

The protocol for this trial and supporting CONSORT checklist are available as supporting information; see Checklist S1 and Protocol S1.

### Patients

This study was performed in accordance with Good Clinical Practice and followed applicable patient privacy requirements and the Declaration of Helsinki. The study protocol was approved by the medical ethical committees at all participating institutions, and patients provided written informed consent prior to participation. A list of all participating institutions and their medical ethics committees is provided in Appendix S1.

Initial patient eligibility (March 2007) included histologically confirmed, poorly differentiated, advanced or metastatic gastric adenocarcinoma, including signet ring cell GC and non-squamous, non-sarcomatous tumors of the gastroesophageal junction (GEJ). In April 2008, the study protocol was amended to include patients with moderately and well-differentiated gastric/gastroesophageal junction tumors and patients with distal esophageal adenocarcinomas. Patients were required to have measurable disease per Response Evaluation Criteria in Solid Tumors (RECIST) (Version 1.0) [28], Eastern Cooperative Oncology Group (ECOG) performance status 0–2 and adequate renal, hepatic, hematologic and adrenocortical function. Individuals with known brain metastases, or more than three lines of prior cytotoxic chemotherapy for locally advanced or metastatic disease were excluded.

### Study Design and Treatment

This was a single-arm, multicenter, Phase II study examining sequentially two dosing regimens of oral foretinib bisphosphate (hereafter referred to as foretinib): intermittent (240 mg/day for 5 consecutive days every 2 weeks) and daily dosing (80 mg/day during each 2-week cycle). Daily dosing cohort enrollment commenced after enrollment into the intermittent dosing cohort was completed. Laboratory evaluations and physical examinations were conducted before each 2-week dosing cycle for both cohorts, and tumors were assessed after 8 weeks of treatment and approximately every 8 weeks thereafter. Safety evaluations were scheduled 30, 90 and 180 days after the last foretinib dose.

### Efficacy Outcome Measures

The primary efficacy outcome was objective response rate (ORR) defined as the proportion of subjects for whom best objective response was a confirmed complete response or confirmed partial response by RECIST [28]. Secondary efficacy measures were progression-free survival (PFS), disease stabilization rate, duration of stable disease (SD) and OS.

### Safety Outcome Measures

Toxicity grade of adverse events (AEs) and laboratory variables were defined according to the National Cancer Institute Common Terminology Criteria for Adverse Events (CTCAE) version 3.0.

### Correlative Studies

Correlative objectives were to assess *MET* gene amplification in GC, to determine the response to foretinib of tumors bearing *MET* amplification, to assess foretinib targeting of MET and to evaluate potential plasma biomarkers of foretinib activity. Analysis of *MET* amplification at 7q31 was performed on archival or recently prepared paraffin-embedded tumor biopsies by fluorescent *in situ* hybridization (FISH) analysis (CarisDX, Phoenix, AZ). Polysomy was differentiated from gene amplification using the ratio of ≥2 for the average copy number of *MET* and CEP 7 (chromosome 7 centromeric marker). PD markers of clinical activity (e.g. phospho-MET [pMET]) were assessed in paired fresh tumor biopsy samples collected at baseline and 5–8 days after starting foretinib treatment in the daily cohort.

Plasma PK levels of soluble MET (sMET), HGF, soluble VEGFR2 (sVEGFR2) and VEGF-A were assessed using electrochemiluminescent two-site immunoassays (Meso Scale Discovery, Gaithersburg, MD). Plasma samples were analyzed for foretinib using an analytical method based on liquid-liquid extraction, followed by high performance liquid chromatography/tandem mass spectrometry analysis.

#### MKN-45 human gastric carcinoma xenograft studies

Female athymic nude mice were housed in the Piedmont Research Center in compliance with the recommendations of the NIH Guide for Care and Use of Laboratory Animals.

Xenografts were established by subcutaneous implantation of 1×10^7^ MKN-45 cells suspended in 50% Matrigel into the right flanks of test mice. Tumor volumes were calculated using the equation (l×w^2^)/2, where l and w refer to the larger and smaller dimensions obtained from caliper measurements at the indicated days post-implantation. For efficacy studies, treatments began when group mean tumor volumes reached ∼180 mm^3^. The vehicle for foretinib is 1% hydroxypropyl methylcellulose (low viscosity grade, E3): 0.2% SLS (sodium lauryl sulfate, HPLC grade): 98.8% water. Mice bearing MKN-45 gastric tumors (n = 10 per group) were treated with vehicle or foretinib once per day (qd) for 21 days at 6 mg/kg or 10 mg/kg, or with 30 mg/kg every other day (q2d) for 42 days or left untreated. The Kruskal-Wallis with Dunn's post-hoc statistical test was performed.

* *P*<0.05 was considered significant.

For pMET/MET and PK analyses, nude mice with MKN-45 tumors measuring 200 to 300 mm^3^ in size received vehicle, foretinib or pazopanib orally once daily for 3 days. Foretinib was quantified by HPLC-MS/MS in plasma samples collected during the 24-hour sampling period after three consecutive daily doses. Tumors were harvested 1, 2, 4, 8 or 24 hours after the last dosing. Phosphorylation of MET in MKN-45 tumors was determined by immunoprecipitation with anti-MET antibody (Cell Signaling #3127) followed by immunoblotting with anti-phosphotyrosine antibody (Sigma #P5872) and, in parallel, with anti-MET antibody (Invitrogen #18-2257). Images were visualized and quantified at both the 700 and 800 channels using the LI-COR Odyssey Infrared Imaging System. Samples were also analyzed using two-site electrochemiluminescent immunoassays for total MET and pMET. Data were analyzed statistically with two sided t-test, where *P*<0.01 was considered significant.

#### Plasma Sampling Schedule for sMET, HGF, sVEGFR2 and VEGF-A_165_

For patients receiving intermittent dosing, plasma samples were collected pre-dose and 4 hours after dosing on days 1, 5, 43 and 47, and pre-dose only on days 15 and 29. For patients in the daily dosing cohort, plasma samples were collected pre-dose and at 4 hours after dosing on days 1, 8, and 15, and pre-dose only on days 29 and 43. The week 1 pre-dose sample was considered baseline for both cohorts.

#### FISH

Sixty nuclei were analyzed by FISH using a BAC bacterial artificial chromosome probe containing the entire *MET* gene (labeled with red Cy3) and a centromere 7-specific probe, CEP7 (labeled with SpectrumGreen), from Molecular Profiling Institute, now CarisDx (Phoenix, AZ). Cells were analyzed from a minimum of two different regions of the metastatic tumor specimen. *MET* amplification was defined as follows: ratio of at least 2 for the average copy number of *MET* and CEP7 across at least 60 cells or any cells containing multiple copies of the *MET* gene in homogenously staining regions performed by Caris Dx (Phoenix, AZ).

#### Met Functional Profiling

Prometheus Laboratories (San Diego, CA) performed functional profiling of MET. Protein lysates were prepared according to the Prometheus standard operating procedure. Briefly, each tumor sample was treated with 10x volume of “Protein later” lysis buffer, followed by tissue homogenization. Supernatants were collected after centrifuging the tissue homogenate at 16,000 rpm for 15 minutes at 4°C. Protein concentrations were determined using BCA assay. Protein lysates were aliquoted and stored at −70°C prior to COPIA analysis. MET activation and expression were profiled using COPIA, a multiplexed, proximity-based, collaborative immunoassay platform (COPIA, Prometheus Laboratories, San Diego, CA). Immunohistology evaluation was performed using sections made from the available OCT-embedded tissues.

#### Pharmacodynamic biomarker assays

Electrochemiluminescent two-site immunoassays for HGF and sMET were developed using commercially available reagents as described previously (Athauda et al., 2006). Assay kits for similar analysis of plasma sVEGFR2 and VEGF levels were obtained from Meso Scale Discovery (Gaithersburg, MD). The VEGF-A assay system used recognizes VEGF-A isoforms 165 and 121 with comparable affinity; it does not recognize the VEGF family members PlGF or VEGF-D. Recognition of VEGF-B or -C has not been determined. Standard curves made using purified recombinant proteins were included in every 96-well plate, and all assays had an overall coefficient of variation of <10%. All samples and standards were analyzed in triplicate. Raw data were processed and analyzed using Microsoft Excel (2008 for Mac) and GraphPad Prism (V5.0) software packages.

### Statistical Analysis

For each dosing schedule, the study was powered for the alternative hypothesis that ORR with foretinib would be at least 25% against the null hypothesis that it is less than 10% (H_0_: p_o_≤0.10 vs. p_a_≥0.25). To achieve a type I error rate of less than 0.10 and at least 80% power, enrollment of 30 evaluable patients was planned in each cohort. The sample size was based on a binomial distribution calculation of exact probabilities for the error rates under the null and alternative hypotheses. If fewer than eight of the evaluable patients had *MET*-amplified tumors, up to 10 additional evaluable patients could be enrolled to a total of 40 patients.

The safety population included all patients who received ≥1 dose of drug. Efficacy endpoints were analyzed in patients who had a baseline and post-baseline tumor assessment, and received ≥75% of the protocol-mandated doses during the first 8 weeks of treatment, or who, prior to completing the first 8 weeks, discontinued foretinib due to drug-related toxicity or progressive disease.

The number (%) of patients with objective response and SD was summarized including 95% confidence intervals. Time-to-event endpoints, including 95% confidence intervals, were summarized using Kaplan–Meier methods.

Correlative analyses included correlation of foretinib exposure with measures of safety and efficacy. Foretinib exposure was defined as the pre-dose concentration on day 5 for the intermittent cohort and the trough concentration on day 15 for the daily cohort. Robust logistic regression analyses using MM-estimation and subsequent Monte Carlo simulations were used to evaluate the relationship between foretinib exposure and percentage change in tumor size at nadir. Because the PK sampling time points represent different exposure measures for each dosing regimen, efficacy analyses were done separately for each regimen.

For plasma levels of sMET, sVEGFR2, VEGFA and HGF, marker baseline and changes from baseline were analyzed at each time point using analysis of variance, and their relationships with plasma foretinib concentrations and clinical outcome (sum of longest diameter [SLD], PFS and RECIST response) were examined using Spearman analysis. Correlation between plasma PD, dosing and plasma concentrations of foretinib, and PFS and RECIST responses, respectively, were tested using proportional hazards and logistic regression.

## Results

### Patients

**Figure 1:**
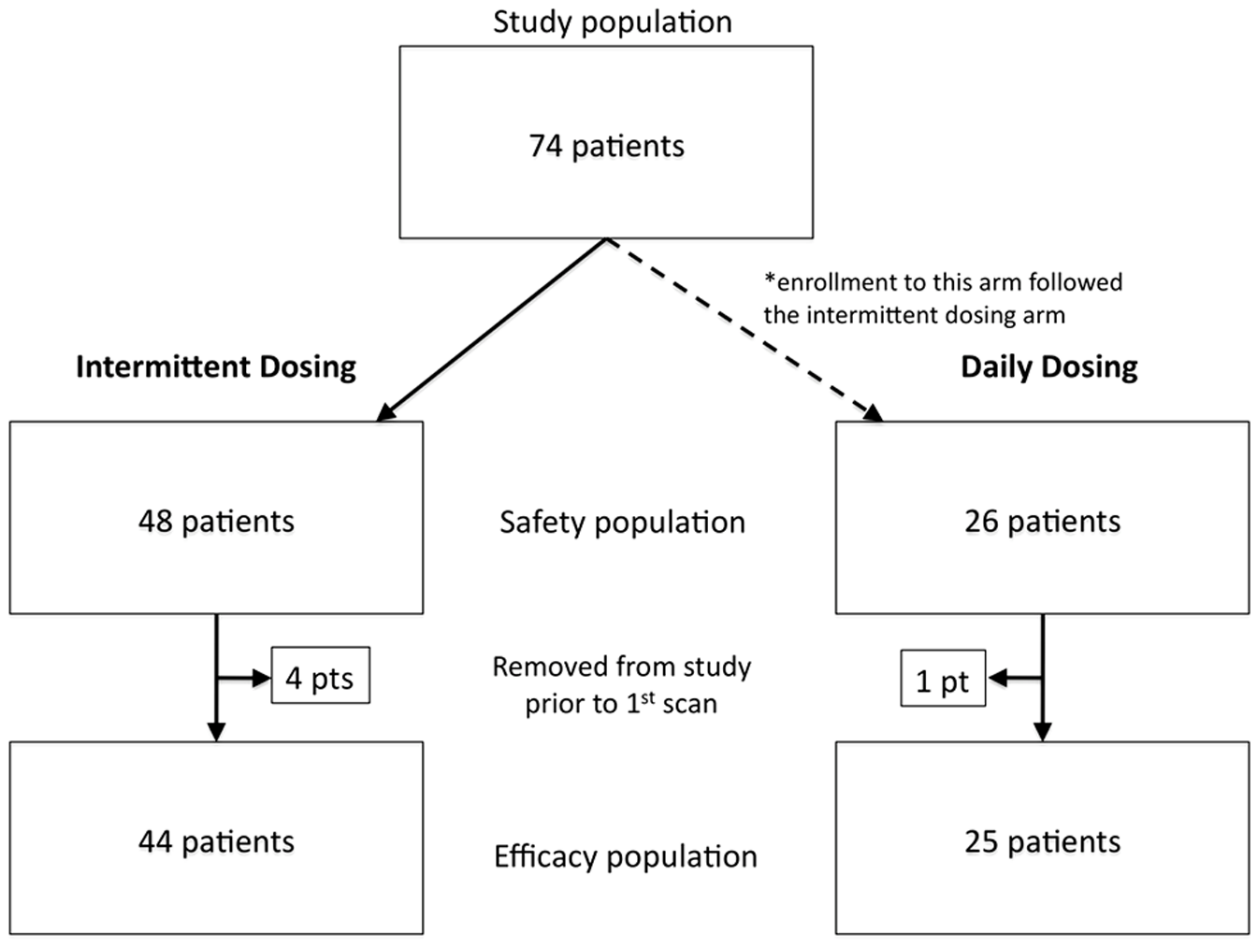
Patient enrollment and participation flow chart. * Patients were enrolled on the Intermittent dosing schedule first, and then enrolled on the daily dosing schedule.

From March 2007 to October 2009, 74 patients were enrolled from 15 participating US centers: 48 in the intermittent dosing cohort and 26 in the daily dosing cohort (Figure 1). Demographics and baseline patient characteristics are provided in Table 1. Sixty-nine patients (44 in the intermittent cohort, 25 in the daily cohort) were evaluable for efficacy. Note, enrollment in the daily cohort stopped when it became apparent that the primary efficacy endpoint would not be met, and that enrollment of 10 additional patients would not yield a significant number of *MET-*amplified patients.

**Table 1:** Demographics, baseline characteristics and cancer history (safety population).

	Intermittent 5/9 dosing cohort	Daily dosing cohort (n = 26)
(n = 48)		
Age, years		
Median (range)	61.0 (25–88)	60.0 (28–82)
Sex		
Male, n (%)	35 (72.9)	20 (76.9)
Race, n (%)		
American Indian or Alaska Native	0	2 (7.7)
Asian	2 (4.2)	2 (7.7)
Black or African American	2 (4.2)	2 (7.7)
White	42 (87.5)	18 (69.2)
Not reported	1 (2.1)	2 (7.7)
Other	1 (2.1)	0
ECOG performance status,^a^ n (%)		
0	21 (43.8)	9 (34.6)
1	25 (52.1)	15 (57.7)
2	2 (4.2)	2 (7.7)
Differentiation,^b^^,^^c^ n (%)		
Moderate	8 (17.3)	7 (27)
Moderate to poor	8 (17.3)	3 (12)
Poor	30 (65.2)	13 (50)
Not reported	0 (0)	3 (11)
Lauren's type,^c^ n (%)		
Diffuse	12 (25)	3 (12)
Intestinal	17 (35)	14 (54)
Mixed	17 (35)	6 (23)
Not reported	0 (0)	3 (11)
Primary site, n (%)		
Gastroesophageal junction	14 (30.4)	12 (46.2)
Cardia	7 (14.6)	2 (7.7)
Antrum	15 (31.3)	3 (11.5)
Distal esophagus	0	2 (7.7)
Other^d^	10 (20.8)	7 (26.9)
Site of metastatic disease,^e^ n (%)		
Bone	2 (4.2)	1 (3.8)
Lymph node	24 (50.0)	14 (53.8)
Liver	27 (56.3)	19 (73.1)
Lung	12 (25.0)	9 (34.6)
Retroperitoneal	3 (6.3)	5 (19.2)
Other^f^	24 (50.0)	8 (30.8)

Abbreviations: ECOG, Eastern Cooperative Oncology Group.

Denominators for percentages are n, the total number of subjects in each dosing cohort or overall.

a The last measurement on or before first dose date.

b All subjects had metastatic GC. In the original protocol, enrolled subjects had to have poorly differentiated diffuse metastatic gastric carcinoma. After a protocol amendment, enrolled subjects did not have to have diffuse pathology; therefore, subjects did not have to have poorly differentiated metastatic GC.

c Tumor differentiation status and Lauren classification were assessed by a central independent pathologist and available in 23 of 26 patients assigned to the daily dosing cohort.

d Curvature, lesser curvature, gastric body, fundus, incisura, pyloric sphincter.

e Some patients had multiple sites of metastatic disease.

f Abdomen, adrenal glands, ascites, colon (adnexa), diaphragm, diaphragmatic surface, falciform ligament, kidney, mesentery, omentum, ovary, pelvis, pericardial effusion, perihepatic tissue, peritoneum, pleura, pleural effusion, portocaval space, presacral mass, rectus abdominus muscle, soft tissue, spleen, suprahepatic adhesion.

The majority of patients were male, white non-Hispanic, with poorly differentiated tumors (one-third Lauren's diffuse histology). Ninety-three percent of patients were previously treated; the median number of prior anticancer therapies was 1, range (1–3), most commonly including a fluoropyrimidine (96%), platinum (84%) or docetaxel (46%). Three patients had *MET* gene amplification and an additional 22% had increased copy number due to polysomy.

### Efficacy

**Figure 2:**
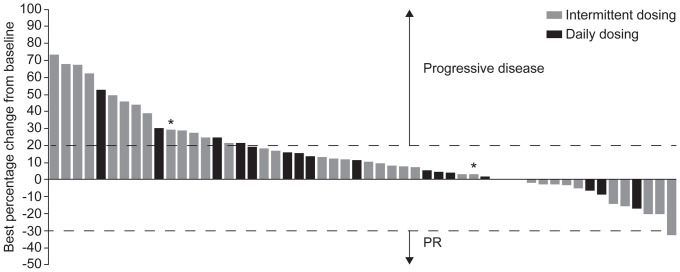
Waterfall plot for best percentage change from baseline in tumor measurement (safety population). *Patients with MET gene amplification (third individual discontinued therapy before tumor measurement).

As shown in Table 2, no patient in either cohort achieved a complete or partial response. Ten (23%) evaluable patients in the intermittent cohort and five (20%) evaluable patients in the daily cohort had a best outcome of SD. The duration of SD (Fig S2A in File S1) ranged from 1.91 to 7.16 months, median duration of 3.2 months. The waterfall plot for response (Figure 2) suggests modest activity observed with single-agent foretinib in previously treated advanced or metastatic GC.

**Table 2:** Tumor response and disease stabilization (evaluable population).

	Intermittent 5/9 dosing cohort (n = 44)	Daily dosing cohort (n = 25)
Subjects achieving best overall response of:		
SD, n (%)	10 (22.7)	5 (20.0)
Progressive disease, n (%)	34 (77.3)	20 (80.0)
ORR		
n (%)	0	0
95% confidence interval^a^	0.0–8.0	0.0–13.7
Disease stabilization rate		
n (%)	10 (22.7)	5 (20.0)
95% confidence interval^a^	11.5–37.8	6.8–40.7

Denominators for percentages are n, the total number of subjects in each dosing cohort. Best overall response was assessed by the investigator per RECIST. The ORR was defined as the percentage of subjects achieving best overall response of confirmed CR or PR. Disease stabilization rate was defined as the proportion of subjects achieving best overall response of confirmed CR or PR or SD.

a Exact confidence intervals were obtained using the Clopper-Pearson method.

There were no tumor responses in the three patients with *MET* gene amplification; one patient experienced SD (2.1 months). A second *MET*-amplified patient was not evaluable for tumor response, having discontinued foretinib due to elevated alanine aminotransferase (ALT) and aspartate aminotransferase (AST) before tumor assessment was performed. The third *MET*-amplified patient had progressive disease. All three patients were in the intermittent cohort. The estimated median PFS was 1.7 months (95% CI: 1.6–1.8 months) overall (1.6 months in the intermittent cohort, 1.8 months in the daily cohort). The estimated median OS was 7.4 months with intermittent dosing and 4.3 months with daily dosing (Fig. S2B in File S1).

### Safety

Table 3 lists treatment-related AEs reported in ≥10% of subjects overall. The intermittent cohort displayed a higher incidence of hypertension, diarrhea, and ALT and AST elevations compared with the daily cohort. Most treatment-related AEs were of mild severity (< grade 3). Ten deaths occurred during treatment or within 30 days of the last foretinib dose; eight patients died due to disease progression, one patient with prior cardiac disease died of a cardiac arrest considered unrelated to foretinib, and one patient death without clear cause was considered possibly related to foretinib. This patient was found at home and a cause of death was not ascertained.

**Table 3:** Treatment-related AEs of all grades reported by ≥10% of patients in either cohort (safety population) and treatment-related AEs of grade 3 and 4.

	Intermittent dosing cohort (n = 48) n (%)		Daily dosing cohort (n = 26) n (%)	
	All grades	Grades 3 or 4	All grades	Grades 3 or 4
≥1 treatment-related AE	45 (93.8)	21 (43.8)	22 (84.6)	9 (34.6)
Fatigue	21 (43.8)	3 (6.3)	12 (46.2)	4 (15.4)
Hypertension	17 (35.4)	2 (4.2)	4 (15.4)	1 (3.8)
Nausea	13 (27.1)	0	7 (26.9)	1 (3.8)
Diarrhea	13 (27.1)	0	3 (11.5)	1 (3.8)
Aspartate aminotransferase increased	11 (22.9)	5 (10.4)	2 (7.7)	0
Vomiting	8 (16.7)	0	5 (19.2)	1 (3.8)
Decreased appetite	7 (14.6)	0	4 (15.4)	1 (3.8)
Alanine aminotransferase increased	7 (14.6)	2 (4.2)	2 (7.7)	0
Blood alkaline phosphatase increased	6 (12.5)	1 (2.1)	1 (3.8)	0
Dizziness	4 (8.3)	0	3 (11.5)	1 (3.8)
γ-glutamyltransferase increased	5 (10.4)	3 (6.3)	2 (7.7)	0
Dysgeusia	6 (12.5)	0	0	0
Dysphonia	5 (10.4)	0	1 (3.8)	0
Rash	3 (6.3)	1 (2.1)	3 (11.5)	0
Abdominal pain	2 (4.2)	0	3 (11.5)	1 (3.8)

Denominators for percentages are n, the total number of subjects in each dosing cohort. At each level of subject summarization, a subject was counted once if the subject reported one or more events. Related events were defined as any event that the investigator assessed as possibly, probably or definitely related to the study drug.

AEs were defined as those occurring or worsening in severity on or after the first dose of foretinib, and no later than 30 days after the last dose. AEs were coded using the Medical Dictionary for Regulatory Activities (MedDRA) Version 10.1.

### PK and PD

Mouse xenograft studies using the human GC-derived *MET*-amplified cell line MKN-45 showed dose-dependent blockade of tumor growth that coincided with significant, durable inhibition of tumor pMET levels (Figure S1 in File S1). The relationship between foretinib PK (i.e. trough samples) and tumor size for each cohort is shown in Fig S3 in File S1. Paired tumor biopsy samples were collected at baseline and at 5 to 8 days after starting treatment with foretinib in the final nine patients enrolled in the daily cohort. Five patients had paired tissue biopsy samples adequate for analysis of MET activation (pMET/total MET) and MET pathway inhibition. Of these five, two had high levels of pMET at baseline, which were substantially reduced after foretinib treatment (>5-fold decrease in pMET/total MET ratio). Despite this, both patients had progressive disease at their first on-treatment scan.

**Figure 3:**
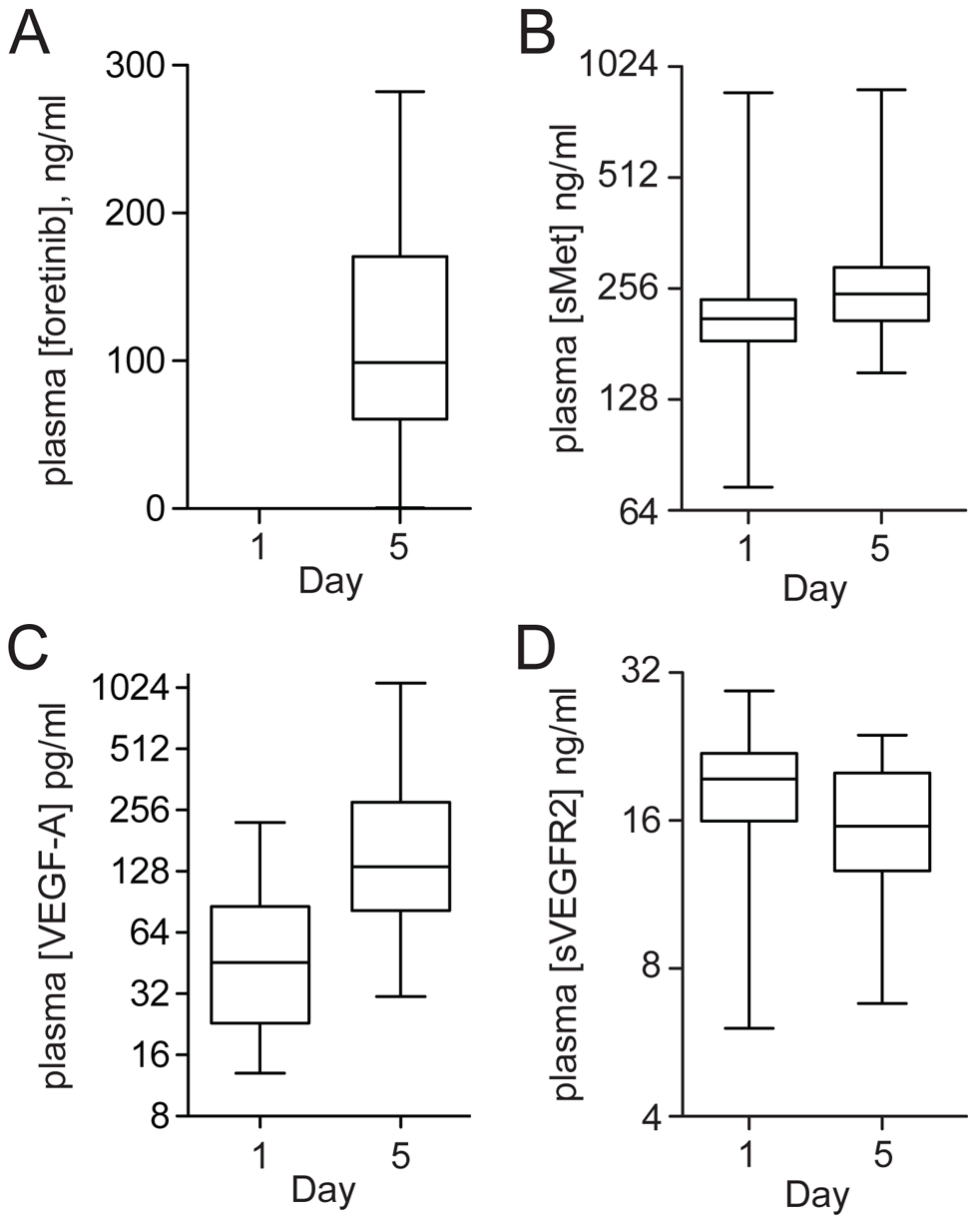
Plasma concentrations of foretinib (A), sMET (B), VEGF-A (C) and sVEGFR2 (D) at days 1 and 5, which encompass the first dosing interval of the intermittent 5/9 dosing group. Box and whisker plots show median ±25% within the box and 100% range of all values within whiskers. Median values for plasma foretinib and each marker shown change significantly over this interval (*P*<0.0001). Other significant marker changes are discussed in the text.

**Figure 4:**
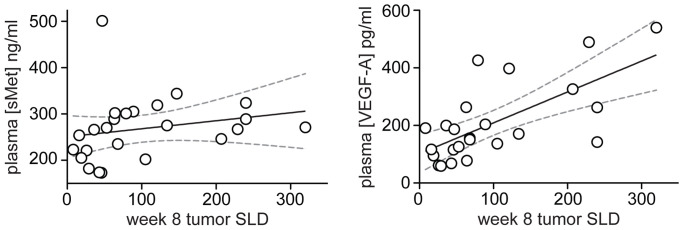
Plasma concentrations of sMET (left) and VEGF-A (right) correlate significantly with tumor burden (sum of longest diameters, SLD) at week 8. Spearman *R* values were 0.5157 (*P* = 0.0099) and 0.6216 (*P* = 0.0012) for sMET and VEGF-A, respectively. The dotted lines indicate 95% confidence intervals.

To further assess foretinib activity, plasma levels of sMET, HGF, sVEGFR2 and VEGF-A were measured at baseline and during treatment. Median concentrations of sMET, sVEGFR2 and VEGF-A changed significantly over the first dosing interval in the intermittent cohort (Figure 3 and Table S1 in File S1). Median sMET and VEGF-A levels correlated with the increase in plasma foretinib (Fig. 2A, B and C), whereas median sVEGFR2 (Fig. 2D) decreased over this interval. Median levels of sMET and VEGF-A in the intermittent cohort decreased over the subsequent 9-day drug holiday (data not shown), suggesting a short-term effect of foretinib. Median HGF levels also increased during dosing periods and decreased during treatment holidays, but rose significantly only over the period from baseline to day 47 (*P*<0.0009). In contrast, circulating levels of sVEGFR2 decreased steadily and significantly over the 47-day period (*P*<0.0001). Plasma concentrations of these markers did not correlate with RECIST response; however, modest but significant correlations were observed between tumor burden at week 8 (SLD) and sMET level (Spearman *R* = 0.5441, *P* = 0.0049), and VEGF-A level (Spearman *R* = 0.6216, *P* = 0.0012), respectively (Figure 4).

## Discussion

GC is an aggressive and common disease for which new therapies are desperately needed. We examined the efficacy of single-agent foretinib, a novel oral inhibitor of MET, RON, AXL, TIE-2 and VEGF2R RTKs, in patients previously treated for metastatic GC. As the first clinical evaluation of MET inhibition in GC, important conclusions were drawn from this study. We show that targeting the MET pathway can be safe and well tolerated in patients with advanced disease. The most common foretinib-related AEs (fatigue, hypertension and gastrointestinal problems) were readily manageable. The most common foretinib-related laboratory abnormalities (elevated ALT and AST) were asymptomatic. Hypertension, a dose-limiting AE for foretinib, is thought to result from its anti-VEGFR activity. Minimal anti-tumor activity was seen with single-agent foretinib, despite evidence of target engagement and tissue evidence of target inhibition.

The two dosing cohorts showed similar safety profiles. Where these differed, the frequency of AEs was generally lower in the daily compared with the intermittent cohort. Although both cohorts demonstrated evidence of pathway inhibition, neither demonstrated significant anti-tumor activity. OS numerically improved in the intermittent cohort compared with the daily cohort. The reason for this is unclear, but the patient populations differed and the number of patients in the daily cohort was small.

We note that fewer than 5% of patients in our study exhibited *MET* amplification. This is lower than the 5% to 23% frequency estimates reported previously for GC tumor specimens [8]–[14]. In contrast, increased *MET* gene copy number due to polysomy 7 (i.e. ≥3 *MET* gene copies across 60 cell nuclei by FISH) occurred in 27% of our study population. Our findings are consistent with another recent evaluation of *MET* gene amplification in localized GC [29] and predict that a low percentage of GCs are driven by *MET* gene amplification.

The minimal efficacy observed with foretinib despite PK and PD evidence of target inhibition further suggests that MET signaling may not be critical in the majority of GC cases without *MET* amplification. Dosing schedules and serum foretinib levels in this study were similar to those observed in a Phase II trial of foretinib for the treatment of papillary renal cell carcinoma, where efficacy of single-agent foretinib was reported [30] (and D. Bottaro, personal communication), suggesting that serum concentrations needed to adequately inhibit foretinib RTK targets were achieved. PD evidence indicates that foretinib did inhibit MET phosphorylation and downstream signaling in patients with high baseline MET phosphorylation. The lack of tumor response in these patients and in the small number of patients with *MET-*amplified tumors suggests that gastric tumors depend on oncogenic signaling pathways other than, or in addition to, MET.

Reinforcing this conclusion, rapid and significant increases in plasma VEGF-A concentrations were observed during dosing periods in the intermittent dosing group, consistent with the reported “class effect” of small-molecule VEGFR inhibitors [31], [32]. These plasma VEGF changes indicate a systemic response to drug and occur maximally at doses providing optimal target kinase coverage [32], [33]. The fact that foretinib inhibits MET and VEGFR with similar potency, and that we observed a similar PD change in sMET, further suggests that adequate MET inhibitory levels of foretinib were achieved. Furthermore, the increase in VEGF-A and sMET significantly correlated with tumor burden (Fig. 3), suggesting that the changes in these PD markers reflect tumor-related foretinib inhibition. The significant negative modulation of plasma sVEGFR2 levels is also consistent with a prior clinical trial with a VEGFR inhibitor that demonstrated anti-tumor activity [33]. Thus, the significant changes in plasma VEGF-A, sMET and sVEGFR2 observed here are consistent with effective target kinase engagement and inhibition. Because median tumor SLD did not change over the course of the study, it remains unclear whether the long-term marker changes observed for sMET, VEGF-A and sVEGFR2 were related, in part, to tumor burden. The significant correlation between week 8 tumor SLD and sMET or VEGF-A levels may suggest such a relationship and warrants further investigation.

It is also possible that oncogenic MET signaling in GC is dynamic and that MET inhibition can be overcome by activation of other signaling pathways. For example, HER kinase activation has been demonstrated to overcome MET tyrosine kinase inhibition in *MET* oncogene-addicted GC preclinical models [34]. Conversely, EGFR inhibition can be overcome by MET pathway activation through *MET* amplification [35], suggesting that these pathways can redundantly activate AKT and promote cell survival. HER2 is overexpressed or amplified in 20% of gastric tumors and is predictive of trastuzumab efficacy [6], suggesting that this may provide a route to tumor cell survival that circumvents MET inhibition in a significant proportion of patients with GC. To assess potential mechanisms of resistance, Cepero et al. exposed human tumor cells that were “MET-addicted” to increasing concentrations of two different MET inhibitors [36]. Cells that developed resistance to these drugs acquired *MET* amplification and subsequent overexpression of wild-type *KRAS*, suggesting that these changes may represent a general resistance mechanism [36]. Foretinib may be more effective if administered at an earlier stage of disease to inhibit invasion and metastasis—known preclinical effects of foretinib.

In summary, this is the first study to evaluate MET, RON, AXL, TIE-2 and VEGFR2 inhibition in GC. Single-agent foretinib showed minimal anti-tumor activity in this unselected, previously treated, advanced or metastatic GC population. Even in patients who were *MET-*amplified or showed elevated pMET and evidence of inhibition on treatment biopsy samples, single-agent foretinib was not associated with significant tumor regression. Future clinical studies targeting MET in gastric tumors should consider enriching the patient population for those with *MET* amplification and evidence of pathway activation. In addition, foretinib or other MET inhibitors may be more effective in combination with other chemotherapeutic or targeted agents with complementary mechanisms of action.

## Supporting Information

Checklist S1 CONSORT Checklist.
(DOC)

Protocol S1 Trial Protocol.
(PDF)

Appendix S1 These are the study sites and the approving ethical review boards for participating sites.
(DOCX)

File S1 This file contains: Table S1. Drug-related modulation of median plasma HGF, sVEGFR2, sMET and VEGF-A concentrations observed over the first dosing interval in the intermittent 5/9 dosing group. Figure S1. Foretinib inhibits gastric tumor xenograft growth and MET activation in mice xenografts. (A) Mice bearing MKN-45 gastric tumors (n = 10 per group) were treated with vehicle alone (black circles) or foretinib once per day (qd; green arrow) for 21 days at 6 mg/kg (green triangles) or 10 mg/kg (green diamonds), or with 30 mg/kg every other day (q2d; blue arrow) for 42 days (blue circles), or left untreated (black squares). Values represent mean ± SEM of tumor volume obtained from caliper measurements at the indicated days. The log-rank test results were significant (P<0.001) for foretinib at all groups when compared with vehicle control. (B) Mice with MKN-45 tumors measuring 200 to 300 mm3 in size received vehicle, foretinib or pazopanib orally once daily for 3 days at the doses indicated. Tumors were collected at the time points indicated (n = 3 per time point) after last dosing, and phospho-MET (pMET) and total MET (MET) were analyzed by immunoblotting; representative tumor samples are shown. (C) pMET/MET ratios (mean +/− SEM) obtained by quantitative (LI-COR) imaging of tumor samples described in panel B. Filled bars, 4 hours post-dose; clear bars, 24 hours post-dose. Asterisks indicate P<0.01 when compared with vehicle control. (D) Two-site electrochemiluminescent immunoassay analysis of pMET/MET ratio (mean +/− SEM) for tumor samples obtained from animals treated with foretinib 30 mg/kg (black squares) or vehicle (black circles) at the indicated times after dosing. Plasma foretinib concentrations (mean +/− SEM; red triangles) were obtained over the same time course. Figure S2A. Duration of stable disease in the evaluable population (all subjects with best response that was not progressive disease). Figure S2B. Overall survival in the evaluable population. Figure S3. Relationship between trough concentrations on (A) day 5 for the intermittent dosing cohort and percentage change in the sum of the longest diameters (pchange), and on (B) day 15 for the daily dosing cohort and percentage change in the sum of the longest diameters (pchange).
(PDF)
