# Correction: Phase II Study Evaluating 2 Dosing Schedules of Oral Foretinib (GSK1363089), cMET/VEGFR2 Inhibitor, in Patients with Metastatic Gastric Cancer

**DOI:** 10.1371/journal.pone.0261994

**Published:** 2021-12-23

**Authors:** Manish A. Shah, Zev A. Wainberg, Daniel V. T. Catenacci, Howard S. Hochster, James Ford, Pamela Kunz, Fa-Chyi Lee, Howard Kallender, Fabiola Cecchi, Daniel C. Rabe, Harold Keer, Anne-Marie Martin, Yuan Liu, Robert Gagnon, Peter Bonate, Li Liu, Tona Gilmer, Donald P. Bottaro

## Notice of republication

This article was republished on October 20, 2020, to remove a Supporting Information file. Please download this article again to view the correct version.
