# Correction: Phase II Study Evaluating 2 Dosing Schedules of Oral Foretinib (GSK1363089), cMET/VEGFR2 Inhibitor, in Patients with Metastatic Gastric Cancer

**DOI:** 10.1371/journal.pone.0276211

**Published:** 2022-10-10

**Authors:** Manish A. Shah, Zev A. Wainberg, Daniel V. T. Catenacci, Howard S. Hochster, James Ford, Pamela Kunz, Fa-Chyi Lee, Howard Kallender, Fabiola Cecchi, Daniel C. Rabe, Harold Keer, Anne-Marie Martin, Yuan Liu, Robert Gagnon, Peter Bonate, Li Liu, Tona Gilmer, Donald P. Bottaro

This Correction provides updates to the linked article’s [1] Methods section, Competing Interests disclosure, and Funding Statement.

The “Correlative Studies” section of the Methods says “Plasma samples were analyzed for foretinib using an analytical method based on liquid-liquid extraction, followed by high performance liquid chromatography/tandem mass spectrometry analysis.” The detailed methods used for these experiments were reported in the following article:

Mohamed W Attwa, Adnan A Kadi, Hany W Darwish, Sawsan M Amer, Haitham Alrabiah (2018) A reliable and stable method for the determination of foretinib in human plasma by LC-MS/MS: Application to metabolic stability investigation and excretion rate. Eur J Mass Spectrom (Chichester). 24(4):344–351. Epub 2018 Apr 8. PMID: 29629565; DOI: 10.1177/1469066718768327.

The “Pharmacodynamic biomarker assays” section of the Methods includes a citation to a 2006 article by Athauda et al., which was not included in the References section. This citation refers to:

Athauda G, Giubellino A, Coleman JA, Horak C, Steeg PS, Lee M-J, Trepel J, Wimberly J, Sun J, Coxon A, Burgess TL, Bottaro DP. (2006) c-Met Ectodomain Shedding Rate Correlates with Malignant Potential. Clinical Cancer Research 12(14): 4154–4162

The Competing Interests statement incorrectly declared that there were no patents to declare, and it came to light after the article [1] was published that additional relevant information should be added to the disclosure. The Competing Interests statement is updated to:

Dr. Kallender, Dr. Martin, Dr. Y. Liu, Dr. Gagnon, Dr. L. Liu, and Dr. Gilmer are employees of, and have equity interest in, GlaxoSmithKline. Dr. Keer is an employee of FivePrime Therapeutics Inc. with equity interest in Exelixis. Exelixis originally discovered foretinib and exclusively licensed foretinib/XL880 to GlaxoSmithKline (GSK) in 2002. That product development and license agreement ran until 2014. Exelixis played no role in the clinical trial reported in [1]. In 2007, NCI and Exelixis had a Clinical Trial Agreement for a potential collaboration involving foretinib development under a separate papillary renal cell carcinoma study; NCI and Exelixis did not move forward with the study proposed under that agreement. Editorial support in the form of development of draft outline, development of manuscript first draft, assembling tables and figures, collating author comments, copyediting and referencing was provided by Ann Sherwood, PhD of CONNEXION Healthcare, Newtown, PA, and by MediTech Media, Manchester, UK, and was funded by GlaxoSmithKline. There are no marketed products to declare. D. Bottaro is an inventor on US Government held patents generally related to this report: US 5,648,273 and related international WO/1992/013097 ("The Hepatocyte Growth Factor Receptor is the Met Proto-Oncogene Product") and US 7,964,365 and related international WO/2007/ 056523 (“Methods for Diagnosing and Monitoring the Progression of Cancer”). This information does not alter the authors’ adherence to *PLOS ONE* policies on sharing data and materials, as detailed online in the guide for authors.

The Funding Statement is updated to:

Funding for this study was provided by GlaxoSmithKline. This work was also supported in part by the Intramural Research Program of the NIH, National Cancer Institute, Center for Cancer Research. The funders were intimately involved in study design, data collection, and analysis. They had no role in the decision to publish, but they were involved in manuscript preparation.
