# Correction: Correction: Phase II Study Evaluating 2 Dosing Schedules of Oral Foretinib (GSK1363089), cMET/VEGFR2 Inhibitor, in Patients with Metastatic Gastric Cancer

**DOI:** 10.1371/journal.pone.0276650

**Published:** 2022-10-20

**Authors:** The PLOS ONE Editors

## Notice of republication

This article was republished on October 10, 2022, to correct an error. Please download this article again to view the correct version.
