# Supplementary material for: Phase II Study Evaluating 2 Dosing Schedules of Oral Foretinib (GSK1363089), cMET/VEGFR2 Inhibitor, in Patients with Metastatic Gastric Cancer
Source: PLoS One. 2013 Mar 14;8(3):e54014. doi: 10.1371/journal.pone.0054014 (PMC3597709; doi:10.1371/journal.pone.0054014)
Supplement: Protocol S1 — Trial Protocol. (PDF) [file pone.0054014.s002.pdf]

CLINICAL STUDY PROTOCOL

A Phase 2 Study of GSK1363089 (XL880) Administered Orally to Subjects with Metastatic Gastric Cancer

PROTOCOL NUMBER: MET111643 (formerly XL880-204)

STUDY DRUG: GSK1363089 (formerly XL880)
IND NUMBER: 71,390

SPONSOR: GlaxoSmithKline
1250 South Collegeville Road
Collegeville
PA 19426, USA

GSK STUDY PHYSICIAN: Stewart McCallum, MD

SECONDARY GSK STUDY PHYSICIAN Sadhna Shankar, MD

GLOBAL STUDY LEADER Howard Kallender, PhD

DATE FINAL: 20 October 2006

**DATE AMENDED:**	13 April 2007	**AMENDMENT NUMBER:**	1.0
**DATE AMENDED:**	01 October 2007	**AMENDMENT NUMBER:**	2.0
**DATE AMENDED:**	09 April 2008	**AMENDMENT NUMBER:**	3.0
**DATE AMENDED:**	24 October 2008	**AMENDMENT NUMBER:**	4.0

CONFIDENTIAL UM2008/00140/01
MET111643

PROTOCOL APPROVAL PAGE

**PROTOCOL TITLE:**	A Phase 2 Study of GSK1363089 (XL880) Administered Orally to Subjects with Metastatic Gastric Cancer
**PROTOCOL NUMBER:**	MET111643 (formerly XL880-204)
**STUDY DRUG:**	GSK1363089 (formerly XL880)
**IND NUMBER:**	71,390
**SPONSOR:**	GlaxoSmithKline<br>1250 South Collegeville Road<br>Collegeville<br>PA 19426, USA
**GSK STUDY PHYSICIAN:**	Stewart McCallum, MD
**SECONDARY GSK STUDY PHYSICIAN**	Sadhna Shankar, MD
**GLOBAL STUDY LEADER**	Howard Kallender, PhD
**DATE FINAL:**	20 October 2006

**DATE AMENDED:**	13 April 2007	**AMENDMENT NUMBER:**	1.0
**DATE AMENDED:**	01 October 2007	**AMENDMENT NUMBER:**	2.0
**DATE AMENDED:**	09 April 2008	**AMENDMENT NUMBER:**	3.0
**DATE AMENDED:**	24 October 2008	**AMENDMENT NUMBER:**	4.0

Approval of protocol by sponsor:

[signature] 10/24/08

Stewart McCallum, MD — Date
Director, Oncology Clinical Development

[signature] Oct. 24 2008

Michael Arning, MD — Date
Vice President, Oncology Clinical Development

10/28/2008
Page 2 of 107

PROTOCOL ACCEPTANCE FORM

**PROTOCOL TITLE:**	A Phase 2 Study of GSK1363089 (XL880) Administered Orally to Subjects with Metastatic Gastric Cancer
**PROTOCOL NUMBER:**	GSK1363089 (formerly XL880-204)
**STUDY DRUG:**	GSK1363089 (formerly XL880)
**IND NUMBER:**	71,390
**SPONSOR:**	GlaxoSmithKline<br>1250 South Collegeville Road<br>Collegeville<br>PA 19426, USA
**GSK STUDY PHYSICIAN:**	Stewart McCallum, MD
**SECONDARY GSK STUDY PHYSICIAN**	Sadhna Shankar, MD
**GLOBAL STUDY LEADER**	Howard Kallender, PhD
**DATE FINAL:**	20 October 2006

**DATE AMENDED:**	13 April 2007	**AMENDMENT NUMBER:**	1.0
**DATE AMENDED:**	01 October 2007	**AMENDMENT NUMBER:**	2.0
**DATE AMENDED:**	09 April 2008	**AMENDMENT NUMBER:**	3.0
**DATE AMENDED:**	24 October 2008	**AMENDMENT NUMBER:**	4.0

By my signature below, I hereby state that I have read, and agree to abide by, the instructions, conditions, and restrictions of the protocol and amendment referenced above.

____

Name of Investigator (print)

____

Name of Investigator (signature) Date

SYNOPSIS

Title

A Phase 2 Study of GSK1363089 (XL880) Administered Orally to Subjects with Metastatic Gastric Cancer

Protocol Number

MET111643 (formerly XL880-204)

Sponsor

GlaxoSmithKline

Clinical Phase

Two

Rationale

Gastric cancer is one of the most common types of cancer in the world today. The clinical outcome for this malignancy remains poor, with a 5-year survival rate of only about 20% (all stages). The primary curative option in this clinical scenario is surgical excision. However, a significant unmet medical need exists for patients with unresectable gastric cancer because no effective therapy has yet been identified.

GSK1363089 (formerly XL880) is a new chemical entity that inhibits multiple receptor tyrosine kinases (RTKs) with growth-promoting and angiogenic properties. The primary targets of GSK1363089 are the hepatocyte growth factor (HGF), and vascular endothelial growth factor (VEGF) RTK families (e.g., hepatocyte growth factor receptor (HGFR) protein [c-MET], VEGFR2/kinase domain receptor [KDR], platelet-derived growth factor receptor [PDGFR]).

Because amplification of *c-MET* has been associated with the pathogenesis of gastric cancer, inhibition of c-MET receptor activation by agents such as GSK1363089 may be of therapeutic benefit in this patient population.

Objectives

The primary objectives of this study are as follows:

- To determine the confirmed response rate of GSK1363089 treatment in subjects with gastric carcinoma or adenocarcinoma of the gastroesophageal junction or of the distal esophagus.
- To further evaluate the safety and tolerability of GSK1363089.

The secondary objectives of this study are as follows:

- To assess the disease stabilization rate, progression-free survival, duration of stable disease, and overall survival in subjects with gastric carcinoma or adenocarcinoma of the gastroesophageal junction or of the distal esophagus who are treated with GSK1363089.
- To characterize the pharmacokinetic (PK) and pharmacodynamic parameters of GSK1363089 in subjects with gastric carcinoma or adenocarcinoma of the gastroesophageal junction or of the distal esophagus.

Note: Each of the primary and secondary study objectives will be assessed for each dosing Cohort (1 – Intermittent 5 days on/9 days off dosing; 2 – daily dosing) separately, as well as together.

The exploratory objectives of this study are as follows:

- To characterize the *c-MET* amplification status of patients in this study population and possible associations with response to GSK1363089 therapy. In addition, the mutational status as well as expression of c-MET and related genes and proteins (e.g., RON) and of relevant downstream targets may also be assessed.
- To evaluate the efficacy and safety profiles between two GSK1363089 dosing regimens - intermittent 5-days on/9-days off and daily.

To evaluate the effect of GSK1363089 treatment on potential pharmacodynamic markers of clinical activity in paired tumor biopsies (e.g. phospho-MET).

Study Design

A Phase 2, non-randomized, sequential-cohort, open-label, safety and efficacy study

Number of Subjects

Subjects with a histological diagnosis of metastatic gastric carcinoma or adenocarcinoma of the gastroesophageal junction or of the distal esophagus[1] may be eligible to enroll in this study as follows:

In an initial cohort, subjects will be enrolled in the study, to receive intermittent dosing (5 days on, 9 days off; referred to throughout the protocol as "intermittent 5&9 dosing") of GSK1363089, until a total of 30 subjects is determined to be evaluable (i.e. subjects who received at least 75% of protocol-mandated doses during the 8-week Study Treatment Period, and have a baseline and post-baseline tumor assessment, as well as subjects who require study drug discontinuation due to GSK1363089-related toxicity or have objective evidence of progressive disease prior to completing the 8-week Study Treatment Period). If fewer than 8 of the evaluable subjects have a *c-MET* amplification at 7q31, as defined as ≥3 genomic copies of the *c-MET* gene, as determined by retrospective FISH analysis, then up to 10 extra evaluable subjects will be enrolled, in order to have at least 8 *c-MET*-amplified subjects in total.

Then, once Cohort 1 has completed enrolment, an extra cohort of subjects will be enrolled in the study, to receive daily dosing of GSK1363089, until a total of 30 subjects are determined to be evaluable. If fewer than 8 of the evaluable subjects have a *c-MET* amplification at 7q31, as defined as ≥3 genomic copies of the *c-MET* gene, then up to 10 extra evaluable subjects will be enrolled, in order to have at least 8 *c-MET*-amplified subjects in the daily-dosing cohort.

Additionally, as part of Amendment 4.0, there will be a requirement in Cohort 2 to have at least 4 evaluable subjects who meet a more stringent definition of *c-MET* gene amplification (i.e. ≥2 copies of *c-MET* per copy of chromosome 7). In the event that the enrolment of 30 evaluable subjects in Cohort 2 is completed before the target of 4 subjects with *c-MET* amplification has been reached, then extra subjects, with proven *c-MET* amplification, pre-screened by FISH, will be enrolled in the study, until the target is reached.

This sample size will allow an initial assessment of the tolerability of GSK1363089 in metastatic gastric cancer subjects. Some hypothesis testing will also be possible with

[1] Prior to Amendment 3.0, only subjects with poorly-differentiated gastric carcinoma or adenocarcinoma of the gastroesophageal junction were included. Amendment 3.0 expanded this, 1) to remove the requirement for poorly-differentiated histology, and 2) to include subjects with distal esophageal carcinoma.

regards to the primary efficacy variable, which is the objective response rate (ORR), defined as the proportion of subjects for whom their best overall response is a confirmed CR or confirmed PR by RECIST:

For each Cohort, if at least 6 of the 30 evaluable subjects respond, this regimen of GSK1363089 will have demonstrated a response rate of 20%. This design is used to test the following hypothesis on the ORR:

Ho: $P_o \le 0.10$ versus $P_a \ge 0.25$

Assuming a type I error rate (alpha) of 0.10 and a type II error rate (beta) of 0.20, with 30 evaluable subjects and at least 6 observed responses (≥ 0.20) subjects, there is a 0.0732 chance of falsely concluding this regimen is preliminarily effective if the true response rate is 0.10. And the chance for falsely rejecting the effect of the regimen is 0.2026 if the true response rate is 0.25. If additional enrollment is required and at least 8 of the 40 (≥ 0.20) evaluable subjects respond, then the above two probabilities would be 0.0419 and 0.18196, respectively.

Target Population

Subjects will be eligible for enrollment as defined by the inclusion and exclusion criteria as follows:

Inclusion Criteria

1. The subject has a histologically-confirmed diagnosis of advanced or metastatic gastric carcinoma, or adenocarcinoma of the gastroesophageal junction or of the distal esophagus. Subjects with tumors of the gastroesophageal junction or of the distal esophagus may be eligible provided that the tumor is not of squamous or sarcomatous histology.
2. The subject has measurable disease, defined as at least one lesion that can be accurately measured in at least one dimension (longest diameter to be recorded) as ≥20 mm with conventional techniques or as ≥10 mm with spiral computerized tomography (CT) scan.
3. Ten unstained slides of representative tumor tissue or a paraffin block of tumor, archival or recently-prepared, have been identified for shipment to the central analysis laboratory. These samples will be used initially for determining the *c-MET*-amplification status by FISH.
4. The subject consents to provide paired tumor biopsies, directly prior to commencing study treatment and then between Days 5 and 8. These samples will be used for analysis of pharmacodynamic markers of GSK1363089 activity; they are in addition to the tumor tissue described in Inclusion Criterion 3.

5. The subject is at least 18 years old.
6. The subject has an Eastern Cooperative Oncology Group (ECOG) performance status ≤2.
7. The subject is able to ingest the GSK1363089 capsules.
8. In the adrenocorticotropic hormone (ACTH) stimulation test, the subject has a serum cortisol level ≥20 µg/dL (552 nmol/L) 30-90 minutes after injection of ACTH.
9. The subject has organ and marrow function as follows: absolute neutrophil count (ANC) ≥1500/mm3, platelets ≥100,000/mm3, hemoglobin ≥9 g/dL bilirubin ≤1.5 mg/dL; serum creatinine ≤1.5 mg/dL or ≤1.5 x ULN if no liver involvement (or ≤2 mg/dL or ≤2 x ULN with liver involvement); or calculated creatinine clearance ≥60 mL/min, alanine aminotransferase (ALT) and aspartate aminotransferase (AST) ≤2.5 x ULN if no liver involvement or ≤5 x ULN with liver involvement.
10. The subject is capable of understanding and complying with the protocol and has signed the informed consent document.
11. Sexually active subjects (male and female) must use a medically accepted method of contraception during the course of the study.
12. Female subjects of childbearing potential must have a negative serum pregnancy test at screening.
13. The subject has had no other diagnosis of malignancy (unless non-melanoma skin cancer or a malignancy diagnosed ≥5 years ago, and has no evidence of disease for 5 years prior to the screening for this study).
14. The subject has a QTcB (bazett corrected) or QTcF (Frederica corrected) < 470 msec. If the first ECG reading is ≥470 msec, repeat the ECG twice over a 10 minute period. Then use the average QTc value to determine whether the subject can be included.

Exclusion Criteria

A subject who meets any of the following criteria is ineligible for the study:

1. The subject has received more than two lines of prior cytotoxic chemotherapy for locally advanced or metastatic disease. For the purpose of this protocol, neoadjuvant therapy would not be considered to be prior cytotoxic chemotherapy. In addition, potential subjects who have received prior treatment with a MET signaling inhibitor (e.g. Small molecule MET inhibitors and/or antibodies against HGF) are excluded.
2. The subject has received an investigational drug within 14 days of the first dose of study drug.
3. The subject has received chemotherapy, immunotherapy, or radiation therapy (to ≥25% of his or her bone marrow) within 14 days or has received nitrosoureas or mitomycin C within 6 weeks prior to the scheduled first dose of GSK1363089.
4. The subject has adverse events (AEs) due to investigational drugs or anti-cancer therapy administered more than 21 days prior to enrollment that have not recovered

to Grade ≤1 using National Cancer Institute (NCI) Common Terminology Criteria for Adverse Events (CTCAE) v3.0, with the exception of alopecia greater than grade 1.

5. The subject has known brain metastases.
6. The subject has uncontrolled intercurrent illness including, but not limited to, ongoing or active infection, symptomatic congestive heart failure, unstable angina pectoris, cardiac arrhythmia, or psychiatric illness/social situations that would limit compliance with study requirements.
7. The subject is pregnant or breastfeeding.
8. The subject is known to be positive for the human immunodeficiency virus (HIV).
9. The subject has a previously identified allergy or hypersensitivity to components of the GSK1363089 formulation.
10. The subject is unable or unwilling to abide by the study protocol or cooperate fully with the investigator or designee.

Estimated Study Length

40 months

Estimated Study Dates

November 2006 (first study visit) to February 2010 (last study visit)

Investigational Product Dose/Route/Regimen

GSK1363089 will be supplied as 20-mg, and 100-mg capsules and will be administered orally as a single agent. In Cohort 1, dosing will occur at 240 mg per dosing day on a 5 days on and 9 days off (referred to throughout the protocol as “Intermittent 5&9 dosing”) cycle every 2 weeks for 8 weeks. In Cohort 2, dosing will occur at 80 mg per dosing day on a daily basis, for 8 weeks. In the absence of progressive disease (PD) and unacceptable toxicity, subjects may continue to receive GSK1363089 treatment on the same dosing schedule for up to a total of 2 years at the discretion of the investigator, and beyond 2 years with the approval of the sponsor.

Comparator Drug

None

Procedures

The study will consist of the following procedures:

Pre-Treatment Period: The screening period must occur within 28 days of the first dose of study medication. The screening visit may be counted as the baseline visit if completed within 72 hours of dosing. Archival, unstained slides or a paraffin block of tumor tissue from the subject should be submitted to the central analysis laboratory during this time for assessment of the *c-MET* amplification status. In addition, as part of Amendment 4.0, a pair of fresh, frozen tumor tissue samples will be collected for pharmacodynamic assessment of GSK1363089's clinical activity; the first sample collected as soon as possible prior to commencing study treatment, and the second sample collected at Day 5.

Study Treatment Period: The 8-week Study Treatment Period will consist of four 2-week treatment cycles. Laboratory evaluations and physical examinations will be conducted before each dosing cycle. GSK1363089 treatment will be stopped if there is disease progression at any time. Tumor assessment will occur at the end of the treatment period (8 weeks).

Treatment Extension Period: In the absence of PD and unacceptable toxicity, subjects may continue to receive study drug on the same dosing schedule for up to a total of 2 years at the discretion of the investigator and beyond 2 years with the approval of the sponsor. Tumor assessment will continue approximately every 8 weeks that the subject remains on study.

Post-Treatment Period: Subjects will return to the study site 30 (±4) days after receiving the last dose of study drug for an end-of-treatment assessment. On Days 90 and 180 (±15 days) after the last dose of study drug or until death (if before Day 180), the investigator (or designee) will obtain follow-up information regarding subject survival.

Assessments of Efficacy

Tumors will be assessed by standard methods (e.g., CT or magnetic resonance imaging [MRI]) at baseline, and then approximately every 8 weeks thereafter in the absence of clinically evident progression of disease. A consistent methodology should be used for the evaluation of each individual subject. Responses will be confirmed with a follow-up tumor assessment at ≥28 days.

Assessments of Safety

Safety will be assessed by standard clinical and laboratory tests (hematology, blood chemistry, and urinalysis). ACTH stimulation and TSH tests, plus a complete eye

examination will be performed prior to and at intervals during dosing with GSK1363089. Toxicity grade is defined by the NCI CTCAE v3.0.

Pharmacokinetics/Pharmacodynamics

In Cohort 1, blood samples for PK and pharmacodynamic analysis will be collected during the 8-week Study Treatment Period at the following timepoints: pre-dose and at 4 hours post-dose on Days 1, 5, 43, and 47; and pre-dose on Days 15 and 29. In Cohort 2, blood samples for PK and pharmacodynamic analysis will be collected at the following timepoints: pre-dose and at 4 hours post-dose on Days 1, 8 and 15, then pre-dose on Days 29 and 43.

In all subjects enrolled after Amendment 4.0 is approved, the effect of GSK1363089 on the expression of pharmacodynamic markers (e.g. total c-MET, phospho-MET and downstream markers) will be evaluated in paired tumor biopsies collected pre-treatment and at Day 5.

Statistical Methods

The primary efficacy endpoint is objective response rate (ORR). ORR will be estimated for the safety and the evaluable populations. A corresponding exact 95% confidence interval will be calculated.

Secondary efficacy endpoints are disease stabilization rate (DSR), progression-free survival (PFS), duration of stable disease, and duration of overall survival (OS). DSR will be summarized using a point estimate of the proportion and the exact 95% confidence interval. PFS, OS, and duration of stable disease will be summarized using Kaplan-Meier methods.

For the primary safety endpoint, AEs and serious adverse events (SAEs) will be tabulated by system organ class and preferred terms. Laboratory test results after the first dose will be summarized using change from baseline values and shift from baseline tables.

Each endpoint will assessed both in the combined Cohorts and in each Cohort separately.

TABLE OF CONTENTS

PROTOCOL APPROVAL PAGE 1

PROTOCOL ACCEPTANCE FORM 2

SYNOPSIS 3

Title 3
Protocol Number 3
Sponsor 3
Clinical Phase 3
Rationale 3
Objectives 4
Study Design 4
Number of Subjects 5
Target Population 6
Estimated Study Length 8
Estimated Study Dates 8
Investigational Product Dose/Route/Regimen 8
Comparator Drug 8
Procedures 8
Assessments of Efficacy 9
Assessments of Safety 9
Pharmacokinetics/Pharmacodynamics 10
Statistical Methods 10

LIST OF ABBREVIATIONS 17

1. BACKGROUND AND RATIONALE 19
1.1. Gastric Cancer 19
1.2. Gastric Cancer Biology and MET 19
1.3. GSK1363089 20
1.3.1. Genotoxic Potential 22
1.3.2. Nonclinical Toxicology 22
1.3.2.1. Single-Dose Toxicology Studies 22
1.3.2.2. Single-Dose Toxicokinetic Analyses 24
1.3.2.3. Repeat-Dose Toxicology Studies 24
1.3.2.4. Repeat-Dose Toxicokinetic Analyses 25
1.3.3. Clinical Data from GSK1363089 26
1.3.3.1. GSK1363089 Studies and Methodology 26

1.3.3.2. Study Methodology....26
1.3.3.3. GSK1363089 Clinical Safety Profile....27
1.3.3.4. GSK1363089 Clinical Pharmacokinetics....32
1.3.3.5. GSK1363089 Clinical Observations in Study XL880-001....32
1.3.3.6. GSK1363089 Clinical Pharmacodynamics....33
1.4. Rationale....33
1.4.1. Rationale for the Study and Study Design....33
1.4.2. Rationale for Dosage Selection....34

2. STUDY OBJECTIVES....35

3. STUDY DESIGN....36
3.1. Overview of Study Design....36
3.1.1. Pre-Treatment Period....36
3.1.2. Study Treatment Period....37
3.1.3. Treatment Extension Period....37
3.1.4. Post-Treatment Period....38
3.2. Study Sites....38
3.3. Treatment Assignment....38
3.3.1. Treatment Groups....38
3.4. Dose Reduction or Treatment Delay for Toxicity....41
3.4.1. Symptoms/Signs Unrelated to Study Drug....41
3.4.2. Symptoms/Signs Related to Study Drug....41
3.4.3. Dose Reduction in Cohort 1 (Intermittent 5&9 Dosing)....42
3.4.4. Dose Reduction in Cohort 2 (Daily Dosing)....42
3.5. Requirement for Collection of Paired Tumor Biopsies....42

4. STUDY POPULATION....43
4.1. Target Population....43
4.2. Inclusion Criteria....43
4.3. Exclusion Criteria....45
4.4. Concomitant Medication and Treatment....45
4.4.1. Anticancer Treatment....45
4.4.2. Supportive Care....46
4.4.3. Management of Treatment-Emergent Hypertension....46
4.4.4. Management of Treatment-Emergent Hepatotoxicity....47
4.5. Potential Drug Interactions....50

5. STUDY ASSESSMENTS AND PROCEDURES....51
5.1. Overview....51
5.2. Pre-Treatment Period....52

5.3. Study Treatment Period 54
5.3.1. Week 1, Day 1 — First Dose of Study Drug 54
5.3.1.1. Pre-Dose Evaluations 54
5.3.1.2. Administration of GSK1363089 55
5.3.1.3. Post-Dose Evaluations 55
5.3.2. Week 1, Day 5-8 (Fifth-Eighth Dose of Study Drug) 55
5.3.3. Week 1, Day 5 (Fifth-Eighth Dose of Study Drug) For Intermittent 5&9 Regimen; Week 2, Day 8 (Eighth Dose of Study Drug) For Daily Dosing Regimen 55
5.3.3.1. Pre-Dose Evaluations 55
5.3.3.2. Administration of GSK1363089 56
5.3.3.3. Post-Dose Evaluations 56
5.3.4. First Dose of Subsequent 2-Week Treatment Cycles (Study Treatment Period Weeks 3 [Day 15], 5 [Day 29], and 7 [Day 43]) 56
5.3.4.1. Pre-Dose Evaluations 56
5.3.4.2. Administration of GSK1363089 57
5.3.4.3. Post-Dose Evaluations 57
5.3.5. Week 7 [Day 47] For Intermittent 5&9 Regimen Only 57
5.3.5.1. Pre-Dose Evaluations 57
5.3.5.2. Administration of GSK1363089 58
5.3.5.3. Post-Dose Evaluations 58
5.3.6. Week 8 58
5.4. Treatment Extension Period 59
5.5. Post-Treatment Period 60
5.5.1. End-of-Treatment Visit 60
5.5.2. Extended Follow-Up Period 60
5.6. Laboratory Assessments 61
5.6.1. Liver Function Tests 62
5.6.2. Urinalysis 62
5.6.3. ACTH Stimulation Test 62
5.6.4. Thyroid-Stimulating Hormone (TSH) Test 63
5.6.5. Ocular Assessments 63
5.6.6. 12-Lead Electrocardiogram (ECG) 65
5.6.6.1. At Screening 65
5.6.6.2. While on Study Drug 66
5.7. Pharmacokinetic Assessments 66
5.8. Pharmacodynamic Assessments - Blood 67
5.9. Tumor Assessments 67
5.9.1. Archival Tumor Tissue 67

5.9.2. Fresh Frozen Tumor Tissue - Paired Tumor Biopsies 68
5.9.3. Tumor Response Assessment 69
5.9.3.1. Routine Tumor Assessment 69
5.9.3.2. Confirmation of Tumor Response 69
5.9.4. Central Pathology Review 70
5.10. Withdrawal Criteria 70
5.10.1. Managing Subject Withdrawals 70
5.10.2. Replacements 71
5.11. Warnings and Precautions..71

6. INVESTIGATIONAL PRODUCT...72
6.1. Dose and Schedule of Study Drug and Comparator(s) 72
6.2. GSK1363089 Capsules 72
6.3. Blinding and Randomization 73
6.4. Compliance 73
6.5. Study Drug Accountability 73

7. SAFETY 73
7.1. Adverse Events and Laboratory Abnormalities...73
7.1.1. Clinical Adverse Events...73
7.1.2. Laboratory Test Abnormalities 74
7.2. Handling of Safety Parameters 74
7.2.1. Serious Adverse Events 74
7.2.2. Treatment and Follow-Up of Adverse Events 76
7.2.3. Follow-Up of Abnormal Laboratory Test Values...............................76
7.2.4. Pregnancy...76

8. STATISTICAL METHODS, ANALYSES, AND DETERMINATION OF SAMPLE SIZE..76
8.1. Statistical and Analytical Plans...76
8.1.1. Analysis Populations...77
8.1.2. Primary Efficacy Analysis 77
8.1.3. Secondary Efficacy Analyses 77
8.1.4. Safety Analysis 78
8.2. Determination of Sample Size 79
8.3. Pharmacokinetic Analysis...80
8.4. Pharmacodynamic Analysis...80

9. DATA QUALITY ASSURANCE 80

10. STUDY COMMITTEES 81

11. ETHICAL ASPECTS 81
  11.1. Local Regulations 81
  11.2. Informed Consent....................81
  11.3. Institutional Review Board 82

12. CONDITIONS FOR MODIFYING THE PROTOCOL....................82

13. CONDITIONS FOR TERMINATING THE STUDY 83

14. STUDY DOCUMENTATION, CRFS, AND RECORD KEEPING 83
  14.1. Investigator's Files and Retention of Documents....................83
  14.2. Source Documents and Background Data 84
  14.3. Audits and Inspections....................84
  14.4. Case Report Forms....................84

15. MONITORING THE STUDY....................84

16. CONFIDENTIALITY OF TRIAL DOCUMENTS AND SUBJECT RECORDS 85

17. PUBLICATION OF DATA AND PROTECTION OF TRADE SECRETS....................85

18. REFERENCES 86

LIST OF TABLES

Table 1 GSK1363089 ED_{50} Values in Tumor Models 21

Table 2 Management of Treatment-Emergent Hypertension 47

Table 3 Liver Enzyme Entry Criteria 48

Table 4 Tumor Scanning Criteria.....69

Table 5 GSK1363089 Capsule Components.....72

Table 6 Composition of GSK1363089 Capsule Shells.....72

Table 7 Lower Bounds for 1-Sided Confidence Intervals.....80

Table 8 Evaluation of Best Overall Response (for RECIST-Based Response Assessment) 99

LIST OF APPENDICES

PAGE

Appendix A: Study Assessments.....89

Appendix A: Study Assessments (continued) 90

Appendix B: Pharmacokinetic and Pharmacodynamic Assessments.....91

Appendix C: Performance Status Criteria 92

Appendix D: Concomitant drugs that should be avoided unless clinically necessary.....93

Appendix E: Urine Protein Creatinine Ratio (UPC) 96

Appendix F: The Response Evaluation Criteria in Solid Tumors (RECIST) Guidelines 97

Appendix G: Liver Chemistry Stopping and Follow-up Criteria.....101

Appendix H: Summary of Changes.....102

LIST OF ABBREVIATIONS

ACTH	adrenocorticotropic hormone
AE	adverse event
ALP	alkaline phosphatase
ALT	alanine aminotransferase
ANC	absolute neutrophil count
AST	aspartate aminotransferase
AUC	area under the plasma drug concentration time curve
BP	blood pressure
BUN	blood urea nitrogen
CFR	Code of Federal Regulations
C_{max}	maximum plasma concentration
c-MET	hepatocyte growth factor receptor (HGFR) protein
CNS	central nervous system
CR	complete response
CRF	case report form
CRO	Contract Research Organization
CT	computerized tomography
CTCAE	Common Terminology Criteria for Adverse Events
CYP	cytochrome P450
DLT	dose-limiting toxicity
DSMC	Data and Safety Monitoring Committee
ECG	electrocardiogram
ECOG	Eastern Cooperative Oncology Group
ED_{50}	dose required for 50% tumor inhibition
FDA	Food and Drug Administration
FLT	fms-like tyrosine kinase
gDNA	genomic deoxyribonucleic acid
GCP	Good Clinical Practice
GGT	γ-glutamyltransferase
GI	gastrointestinal
GLP	Good Laboratory Practice
GSK	GlaxoSmithKline
HGF	hepatocyte growth factor
HIV	human immunodeficiency virus
IC_{50}	concentration required for 50% inhibition
ICH	International Conference on Harmonisation

LIST OF ABBREVIATIONS (continued)

IND	Investigational New Drug
INR	International Normalized Ratio
IRB	Institutional Review Board
ITT	intent-to-treat
IV	intravenous
KDR	kinase insert domain receptor
MedDRA	Medical Dictionary for Regulatory Activities
MET	hepatocyte growth factor receptor (HGFR) gene
MRI	magnetic resonance imaging
MTD	maximum tolerated dose
NCI	National Cancer Institute
NOAEL	no observable adverse effect level
PCR	polymerase chain reaction
PD	progressive disease
PDGF(R)	platelet-derived growth factor (receptor)
PI	principal investigator
PK	pharmacokinetic
PR	partial response
PRC	papillary renal-cell carcinoma
PT/PTT	prothrombin time/partial thromboplastin time
QPCR	quantitative polymerase chain reaction
RBC	red blood cell
RECIST	response evaluation criteria in solid tumors
RTK	receptor tyrosine kinase
SAE	serious adverse event
SD	stable disease
t_{max}	time to attain C_{max}
TUNEL	terminal deoxynucleotide transferase-mediated dUTP nick-end labeling
VEGF(R)	vascular endothelial growth factor (receptor)
WBC	white blood cell

1. BACKGROUND AND RATIONALE

1.1. Gastric Cancer

Malignancies of the upper gastrointestinal (GI) tract (esophagus, gastroesophageal junction, and stomach) result in substantial morbidity and mortality. Worldwide, gastric cancer is the second most common malignancy, with an estimate of over 800,000 cases diagnosed and 650,000 resultant deaths (reviewed in Dicken et al. 2005). In the United States, where the incidence of gastric cancer is comparatively low, approximately 22,280 new cases of gastric cancer are expected to be diagnosed in 2006, which will likely result in 11,430 deaths. This disease has become the eighth most common cause of cancer death in the United States (American Cancer Society 2006). Surgical resection is the only curative treatment for gastric cancer. For appropriate patients, surgical management should include complete resection with adequate margins (5 cm). Unfortunately, most patients, when diagnosed, have advanced disease, making surgical resection either not feasible or available only with purely palliative intent (National Comprehensive Cancer Network 2006).

Chemotherapy likely provides some benefit for patients with advanced gastric cancer. In a recent meta-analysis, it was concluded that chemotherapy produces superior survival compared with best supportive care. It was also concluded that combination chemotherapy (usually using a fluorouracil, an anthracycline, and a platinum agent) improves survival compared with single-agent chemotherapy, but with additional toxicity (Wagner et al. 2006). Nonetheless, the prognosis for patients with advanced gastric cancer is dismal, with median survival typically being less than 10 months (Dicken et al. 2005). For patients treated with chemotherapy, there are no effective treatments at the point of disease progression (salvage therapy). Overexpression of hepatocyte growth factor receptor (HGFR) gene (*c-MET*) associated with amplification of the 7q31 chromosomal region has been reported in some gastric cancers and is associated with a poor prognosis (Nakajima et al. 1999). Inhibition of c-MET by a small molecule such as GSK1363089 may be of therapeutic value in patients with advanced gastric cancer who have progressed on their initial chemotherapy regimen.

1.2. Gastric Cancer Biology and MET

Most gastric cancers are adenocarcinomas, and may be classified based on surgical or endoscopic findings (e.g., ulcerative, polypoid, scirrhous) or based on cellular features

(e.g., intestinal, well-differentiated). The natural history of gastric cancer suggests the following two biologically distinct diseases: (1) intestinal histology more commonly occurs in the distal stomach and is ulcerated, and (2) carcinomas are usually located in the cardia and may be described as "linitus plastica" lesions (reviewed in Fuchs and Mayer 1995). Advances in molecular techniques have implicated the receptor for hepatocyte growth factor (HGF), the *c-MET* gene located at 7q, as a gene that is frequently overexpressed in gastric cancers (Tahara 1993; reviewed in Lin et al. 2000). Amplification of *c-MET* was initially described in five gastric cancer cell lines derived from scirrhous gastric cancer, and also in 5 of 13 (38%) scirrhous gastric tissue and 5 of 25 (25%) of poorly differentiated gastric cancer specimens tested (Kuniyasu et al. 1992). The scirrhous classification in this study corresponds to diffusely infiltrating carcinoma showing vast fibrous stroma with rapid and extensive growth. The authors also noted an association between *c-MET* amplifications and stage, in that stage I gastric carcinoma had no amplifications and stage III and stage IV gastric carcinomas had frequent amplification (Kuniyasu et al. 1992). Subsequent reports have consistently identified *c-MET* overexpression or amplification as an independent prognostic indicator that is correlated with the depth of tumor invasion (Nakajima et al. 1999; Huang et al. 2001). The association of c-MET overexpression with histologic subtype is not as clear; however, in the study by Huang et al., 14 of 21 tumors evaluated were identified as being poorly differentiated for positive c-MET expression and having markedly more c-MET than corresponding normal gastric tissue (Huang et al. 2001). Other features associated with poor prognosis (e.g., presence of liver metastases, overexpression of alpha-fetoprotein) have been associated with c-MET overexpression (Amemiya 2000, 2002). Another study showed elevated serum levels of c-MET in patients with advanced disease (Wang et al. 2004). Together, these data suggest that c-MET dysregulation may be important in the pathogenesis of gastric cancer and that inhibition of c-MET is a biologically rational therapeutic approach. GSK1363089 is a novel, small-molecule inhibitor of multiple receptor tyrosine kinases (RTKs) including c-MET, and therefore may be of use in the treatment of metastatic gastric cancer.

1.3. GSK1363089

GSK1363089 is a new chemical entity that inhibits multiple RTKs with growth-promoting and angiogenic properties. The primary targets of GSK1363089 are the receptors for the HGF and vascular endothelial growth factor families (c-MET and vascular endothelial growth factor receptor 2/kinase insert domain receptor

[VEGFR2/KDR], respectively), although GSK1363089 also inhibits platelet-derived growth factor receptor (PDGFR), KIT, RON, fms-like tyrosine kinase 3 (FLT3), and Tie-2.

GSK1363089 is orally bioavailable, as demonstrated by pharmacokinetic (PK) studies in animals and humans. Target modulation studies show that the administration of GSK1363089 in mice causes dose-dependent inhibition of c-Met, KDR, Tie-2, mutationally activated KIT (D816V), and mutationally activated FLT3 (FLT3-ITD). Immunohistochemical studies demonstrate rapid effects on the endothelium and vascular breakdown and tumor cell death within 8 hours after administration of GSK1363089. This effect translates into significant tumor growth inhibition following GSK1363089 treatment in multiple tumor models (Table 1). The models utilizing H441 and B16F10 are notable for their expression of high levels of c-Met protein, indicating potential reliance on signaling pathways involving c-Met for maintaining a tumorigenic phenotype *in vivo* (Christensen et al. 2003). It has also been shown that U87MG cells require c-Met expression for growth *in vivo* (Lal et al. 2005). In addition, in many of the models (e.g., human breast cancer, human colorectal carcinoma, rat glioblastoma), marked tumor regression was observed. In a FLT3-driven model of leukemia (FLT3AC-Ba/F3), median survival time in animals receiving GSK1363089 was increased in a dose-dependent manner up to 289% compared with control animals.

Table 1 GSK1363089 ED_{50} Values in Tumor Models

Tumor Cell Line, Tissue of Origin	Species	ED_{50} (mg/kg/day)	Treatment Duration[a] (days)
C6, rat glioblastoma	rat	2	12
U87MG, human glioblastoma	mouse	2	21
MDA-MB-231, human breast carcinoma	mouse	4	14
HT29, human colon carcinoma	mouse	7	17
H441, human lung carcinoma	mouse	7	18
B16F10, mouse melanoma	mouse	<30	10

ED_{50}, dose required for 50% tumor inhibition.

a Dosing once daily.

GSK1363089 inhibited cellular HGF-induced c-Met phosphorylation and VEGF-induced ERK phosphorylation with concentration required for 50% inhibition (IC_{50}) values of 23 nM and 16 nM, respectively. GSK1363089 inhibited the mutationally activated forms

of FLT3 and KIT found in human tumors (IC_{50}, 5 to 15 nM) and had broad anti-proliferative activities against a panel of tumor cell lines (IC_{50}, 0.150 to 3.1 μM). Additionally, GSK1363089 inhibited PDGFRß with an IC_{50} value of 23 nM in an autophosphorylation assay. GSK1363089 also inhibited anchorage-independent growth of tumor cells (IC_{50}, 29-165 nM) as well as HGF-induced responses in tumor and endothelial cells (chemotaxis, invasion) that are thought to contribute to invasion and metastasis in vivo (IC_{50}, 25-50 nM). Therefore GSK1363089 has the potential to prevent tumor growth in vivo both by effects on tumor cell proliferation and inhibition of the host-angiogenic response.

A summary of GSK1363089 pharmacology is contained in the Investigator's Brochure supplied by GSK (or designee). This document should be reviewed in conjunction with this study protocol.

1.3.1. Genotoxic Potential

A genotoxic impurity, 4-fluoroaniline, has been found to be present in the current clinical batches of bisphosphate (GSK1363089A) drug substance. For subjects in the ongoing trials, the highest possible amount present in a daily dose is 8.4 micrograms (for a subject on the Intermittent 5&9 dosing schedule receiving 240 mg per dosing day) or 2.8 micrograms (for a subject on the daily dosing schedule receiving 80 mg per dosing day); for reference, a current European Medicines Agency guideline allows genotoxin levels of up to 1.5 micrograms per day for drugs taken daily on a chronic basis; http://www.emea.europa.eu/pdfs/human/swp/519902en.pdf). While the presence of these levels of this genotoxic substance are not believed to pose a significant risk to subjects participating in this study, 4-fluoroaniline levels in the drug substance will continue to be monitored until further process understanding is gained.

1.3.2. Nonclinical Toxicology

Toxicity associated with a single or repeat oral dosing of GSK1363089 was characterized in dose range-finding (non-Good Laboratory Practice [GLP]) and definitive (GLP) nonclinical studies in rats and dogs. Toxicokinetic modeling was also performed on plasma drug concentration data derived from these studies.

1.3.2.1. Single-Dose Toxicology Studies

Toxicity associated with single oral doses of GSK1363089 in rodent and non-rodent species was characterized by dose-dependent changes in clinical signs; clinical laboratory

changes reflective of toxicity to liver, hematopoietic, and lymphoid tissues; and histopathologic findings in multiple tissues in treated rats. The minimum lethal oral dose for GSK1363089 was 600 mg/kg in rats (which occurred 4 to 6 days post-dose in five of five treated males), and exceeded 36 mg/kg in dogs (the highest dose tested). Clinical signs of toxicity, which were dose-dependent and generally reversible, included ataxia, sternal or lateral recumbancy, thinness, pale color, rough haircoat, liquid feces, hypoactivity, and hunched posture in rats; liquid, non-formed and/or red discolored feces in rats and dogs; and vomitus in dogs. The lowest single doses of GSK1363089 in which these clinical signs of toxicity were observed were 600 mg/kg in rats, and 18 mg/kg in dogs. GSK1363089 administration resulted in dose-dependent, reversible decreases in body weights and/or food consumption in rats (≥200 mg/kg) and dogs (≥18 mg/kg). Clinical laboratory parameter changes in rats reflected a primary hepatotoxic effect of GSK1363089 at doses of ≥200 mg/kg (increased alanine aminotransferase [ALT], aspartate aminotransferase [AST], and total bilirubin) and ≥600 mg/kg (increased alkaline phosphatase [ALP] and γ-glutamyltransferase [GGT], and decreased total protein and albumin). Correlative dose-dependent histopathologic findings were present in the liver (e.g., hepatocellular necrosis, Kupffer cell necrosis, and megakaryocytosis). Hematology evaluations in rats suggested a primary toxic effect of GSK1363089 on lymphoid and hematopoietic tissues at higher single doses (e.g., lower hemoglobin, hematocrit, red blood cell [RBC], lymphocyte, eosinophil, and platelet counts). Correlative dose-dependent anatomic microscopic lesions were observed in bone marrow (e.g., hemorrhage, necrosis, and depletion) and lymphoid tissues (e.g., lymphoid necrosis and/or depletion of the thymus, spleen, and lymph nodes). However, at lower GSK1363089 doses, many of these hematologic parameters were increased, were reversible upon discontinuation of dosing, and were considered secondary changes consistent with dehydration (e.g., increased RBC counts, hemoglobin, hematocrit) and inflammation (e.g., increased white blood cell [WBC] counts, neutrophils). Rats dosed at ≥600 mg/kg showed significant histopathologic changes in GI tract tissues that were considered likely to be responsible for significant fluid and electrolyte losses and a site of inflammation. In addition, fecal abnormalities observed in dogs dosed at 18 mg/kg were also suggestive of GI tract toxicity. In rats dosed at lethal GSK1363089 doses (≥600 mg/kg), inflammatory and/or necrotic changes present in adrenals, heart, glandular tissue, lung, reproductive tissue, and brain may reflect a primary drug effect or a secondary event associated with general systemic toxicity. At the lower, non-lethal GSK1363089 dose (200 mg/kg), histopathologic changes present in adrenal, liver, lung, bone marrow, and lymphoid tissues in rats are more likely to be direct test article-related

effects. Clinical laboratory changes in rats also suggested a primary adverse effect on renal function (e.g., higher urea nitrogen and creatinine; increased incidence and severity of urinary protein and occult blood); however, no histopathologic effects in kidney tissue were observed. The no observable adverse effect level (NOAEL) for the definitive single oral dose toxicity studies of GSK1363089 in rats and dogs were considered to be <200 mg/kg and 36 mg/kg, respectively.

1.3.2.2. Single-Dose Toxicokinetic Analyses

Toxicokinetic analyses indicated that systemic drug exposure parameters (maximum plasma concentration [C_{max}] and area under the plasma drug concentration-time curve [AUC_{0-t}] values) increased less than dose-proportionally with increasing single oral GSK1363089 doses in rats (200-2000 mg/kg), but increased slightly greater than dose-proportionally with increasing single oral GSK1363089 doses in dogs (6–36 mg/kg). Time to attain C_{max} (t_{max}) ranged from 12 to 24 hours post-dose in rats, and from 2.25 to 6 hours post-dose in dogs. In rats, GSK1363089 cleared slowly from plasma, and no distinct elimination phase could be estimated. At 48 hours post-dose, plasma drug concentrations were approximately 5%, 62%, and 99% of C_{max} at the 200-, 600-, and 2000-mg/kg dose levels, respectively. These findings suggest possible saturation of drug absorption, particularly at the higher (>600 mg/kg) doses. In dogs, plasma half-life ($t_{1/2}$) values ranged from 8.02 to 9.77 hours, and did not change significantly with dose. No significant (>2-fold) gender-related differences in GSK1363089 toxicokinetic parameters were apparent.

1.3.2.3. Repeat-Dose Toxicology Studies

Repeat daily oral dosing of GSK1363089 resulted in dose-dependent toxicity in both rats and dogs. Significant clinical signs of toxicity (e.g., labored breathing hypoactivity, tremors, decreased body weight gains, cold-to-the-touch) preceding moribund changes and/or death were present in rats dosed daily for 12-16 days at 20 mg/kg/day or 9–12 days at 100 mg/kg/day. Significant clinical signs of toxicity (e.g., vomitus, fecal abnormalities, hypoactivity, decreased body weight gains, and decreased food consumption) were also present in dogs dosed daily for 3 days at 10 mg/kg/day. Clinical laboratory changes in rats were generally dose-dependent, were reversible upon discontinuation of treatment, and were consistent with liver injury (increased ALT and GGT), non-specific tissue damage (increased AST), dehydration, pre-renal azotemia, and/or inflammation. Clinical laboratory changes in dogs suggested relatively

non-specific, reversible effects of GSK1363089 administration; these changes included increased RBC count, hemoglobin, hematocrit and cholesterol, and moderately decreased absolute reticulocyte counts at doses of ≥3 mg/kg. At a dose of 10 mg/kg qd × 3 and 6 mg/kg qd × 6 separated by an 11-day non-treatment period, absolute lymphocyte counts, albumin to globulin ratio, and AST values were decreased; globulin levels and incidence and severity of urine occult blood were increased. Histopathologic findings in rats treated at 20 mg/kg/day consisted of generally reversible necrotic, inflammatory, and/or hypertrophic changes present in multiple tissues (e.g., adrenal cortex, GI tract, liver, pituitary, kidney, lung, thymus, spleen, lymph tissue, testis, heart, pancreas, brain, bone, and/or bone marrow). Severe adrenal cortical hemorrhage was considered to be causal to the two unscheduled rat deaths at the 20-mg/kg/day dose. Although these microscopic anatomic changes appear to reflect an overall general debilitated condition, a specific cytotoxic action of GSK1363089 on these tissues cannot be ruled out. Histopathologic changes associated with GSK1363089 administered to dogs at 10 mg/kg qd x 3 and 6 mg/kg qd x 6 separated by an 11-day non-treatment period were observed in the GI tract (e.g., edema, hemorrhage and/or inflammation of stomach, duodenum, and esophagus), lymphoid tissues (depletion/necrosis of spleen, thymus, mesenteric lymph nodes, and GI lymphoid tissue), salivary gland (acinar atrophy), bone (atrophy), and bone marrow (cellular depletion of erythroid elements). These lesions were not evident in animals that were provided a 23-day non-treatment recovery period, and were therefore considered to be reversible. Bone marrow cellular depletion was the primary finding in GSK1363089-treated dogs dosed at 3 mg/kg/day for 14 days. The NOAELs for the repeated oral dose toxicity studies in rats and dogs administered GSK1363089 daily for 14 consecutive days were considered to be <2 mg/kg/day and 1 mg/kg/day, respectively.

Preliminary results from the chronic toxicity studies of GSK1363089 indicate that no significant treatment-related clinical toxicity, hematology or clinical chemistry parameter changes, or histopathologic changes were evident in rats and dogs administered GSK1363089 once daily for up to 26 weeks at doses up to 1 mg/kg/day, the highest doses tested and tentative NOAELs for these studies. Toxicokinetic evaluations for these chronic toxicity studies are ongoing.

1.3.2.4. Repeat-Dose Toxicokinetic Analyses

Toxicokinetic analyses associated with repeated GSK1363089 oral dosing showed that AUC_{0-t} and C_{max} generally increased dose-proportionally in rats (2-20 mg/kg) and slightly

greater than dose-proportionally in dogs (1-10 mg/kg) with increasing GSK1363089 dose following both Day 1 and Day 14 dosing. There was no evidence of significant drug accumulation. Values for t_{max} ranged from 2 to 8 hours post-dose in rats, and from 3 to 4 hours post-dose in dogs. Plasma half-life values ranged from 6.9 to 9.7 hours in rats and dogs, and showed slight increases with dose.

1.3.3. Clinical Data from GSK1363089

1.3.3.1. GSK1363089 Studies and Methodology

GSK1363089 is currently being evaluated in two Phase 1 studies and three Phase 2 studies: (1) XL880-001, a Phase 1 dose-ranging study that is examining the Intermittent 5&9 dosing schedule, (2) XL880-002, a Phase 1 study that is examining a daily dosing schedule, (3) XL880-201, a Phase 2 study that is examining GSK1363089 in subjects with papillary renal-cell carcinoma using the Intermittent 5&9 dosing schedule, (4) XL880-203 that is examining GSK1363089 in recurrent or metastatic squamous cell cancer of the head and neck, and (5) XL880-204, a Phase 2 study that is examining GSK1363089 in subjects with metastatic gastric cancer.

1.3.3.2. Study Methodology

Study XL880-001, “A Phase 1 Dose Escalation Study of the Safety and Pharmacokinetics of GSK1363089 Administered Orally to Subjects with Solid Tumors,” is ongoing. In this study, subjects with advanced solid tumors were enrolled in successive cohorts to receive GSK1363089 orally as a single dose on Day 1 with associated PK sampling, followed by five consecutive daily doses starting on Day 4 with additional PK sampling (all subjects have completed this initial treatment period). Beginning 14 days later, subjects may have been eligible to receive GSK1363089 cyclically, and one subject is currently still receiving study drug during this treatment extension period. Each treatment cycle is 2 weeks (14 days) in duration and consists of GSK1363089 administration for five consecutive days followed by a 9-day observation period. This dosing regimen is referred to throughout the remainder of this protocol as the “Intermittent 5&9” dosing schedule.

Study XL880-002, “A Phase 1 Dose Escalation Study of the Safety and Pharmacokinetics of GSK1363089 Administered Orally Daily to Subjects with Solid Tumors,” is ongoing. In this study, subjects with advanced solid tumors are enrolled in successive cohorts to receive GSK1363089 orally once daily, with PK sampling at various timepoints over a

28-day treatment period. Dosing for the initial cohort was at 60 mg/day and MTD was determined to be 80 mg/day.

Study MET111644 (formerly XL880-201), "A Phase 2 Study of the c-MET RTK Inhibitor GSK1363089 in Subjects with Papillary Renal-Cell Carcinoma (PRC)," is ongoing. In this study, subjects with PRC are enrolled to receive GSK1363089 at 240 mg on an Intermittent 5&9 dosing schedule and on a daily dosing schedule. Subjects are stratified based on germ line *c-MET* mutational status into sporadic or hereditary and somatic PRC.

Study MET111646 (formerly XL880-203), "A Phase 2 Study of the c-MET RTK Inhibitor GSK1363089 in Subjects With Recurrent or Metastatic Squamous Cell Cancer of the Head and Neck (SCCHN)" is ongoing. In this study, subjects with SCCHN are enrolled to receive GSK1363089 at 240 mg per dose on an Intermittent 5&9 dosing schedule. At study entry, subjects will have a tumor sample collected (either previously archived or newly collected with additional informed consent) to be analyzed for c-MET status.

This study, MET111643 (formerly XL880-204), "A Phase 2 Study of GSK1363089 Administered Orally to Subjects with Metastatic Gastric Cancer" is ongoing. In this study, subjects with gastric cancer are enrolled to receive GSK1363089 at 240 mg per dose on an Intermittent 5&9 dosing schedule or at 80 mg per dose on a daily dosing schedule. Subjects are assessed for the presence of 7q31 amplification (the chromosomal region containing *c-MET*).

1.3.3.3. GSK1363089 Clinical Safety Profile

GSK1363089 has been studied using two dosing schedules: Intermittent 5&9 (XL880-001 and MET111644) and daily (XL880-002). With both schedules, hypertension and proteinuria appear to be common GSK1363089-associated effects.

1.3.3.3.1. XL880-001

As of 20 August 2007, 40 subjects enrolled in XL880-001 (A Phase 1 Dose Escalation Study of the Safety and Pharmacokinetics of GSK1363089 Administered Orally to Subjects With Solid Tumors) have been treated with eight dose levels as follows: 0.1 and 0.2 mg/kg (liquid formulation), 0.4 mg/kg (both liquid and capsule formulations), 0.8, 1.6, 2.4, 3.6, and 4.5 mg/kg (capsule formulation). Each cohort received a single oral

dose of GSK1363089 on Day 1 followed by a 72-hour observation period. Then, if there were no GSK1363089-related dose limiting toxicities, subjects received five consecutive daily oral doses of GSK1363089 on Days 4-8. All subjects tolerated their initial dose and received further GSK1363089 treatment.

A total of 676 AEs in 40 subjects have been reported as of 20 August 2007. Thirty-nine (98%) subjects reported at least one AE. Adverse events regardless of relationship to GSK1363089 and reported in ≥20% of subjects were: nausea (45%), diarrhea, fatigue, and vomiting (40% each), hypertension (38%), proteinuria (33%), AST (28%), lactate dehydrogenase (25%), edema peripheral and rash (23% each), and hematuria and constipation (20%).

The majority of the AEs reported in this study were of Grade 1 (436 of 676), 176 were of Grade 2, 54 were of Grade 3, three were of Grade 4. Twenty-seven of 40 subjects (67.5%) reported an AE of Grade 3 or higher. The Grade 4 events were back pain, metastasis to central nervous system, and mental status changes, none of which was considered related to GSK1363089.

For the AEs considered possibly or probably related, the majority were of Grade 1 (172 events), 79 were of Grade 2, and 21 were of Grade 3: elevated LDH reported as blood LDH increased (3 subjects) or blood lactate dehydogenase (1 subject), hypertension (2 subjects), and gamma glutamyl transferase and lipase increased, two subjects each; the remaining related AEs of Grade 3 occurred in one subject each and included vomiting, fatigue, AST, blood alkaline phosphatase increased, tumor hemorrhage, apraxia, confusional state, hematuria, and palmar-plantar-erythrodysaesthesia. There were no reported Grade 4 or 5 AEs assessed as possibly or probably related to GSK1363089 in this study.

A total of 23 SAEs were reported in 15 subjects. Six events in three subjects were assessed as possibly or probably related to study treatment: altered mental status, tumor related hemorrhage, and expressive language disorder, confusion, apraxia, and ataxia.

There were three deaths in subjects on study XL880-001 that occurred within 30 days of the last dose of study drug. These were assessed as not related to GSK1363089 treatment and attributed to disease progression.

The dose of 3.6 mg/kg was well tolerated by the subjects and was subsequently designated the protocol-defined MTD.

1.3.3.3.2. XL880-002

As of 20 August 2007, 24 subjects in study XL880-002 (A Phase 1 Dose-Escalation Study of the Safety and Pharmacokinetics of GSK1363089 Administered Orally Daily to Subjects With Solid Tumors) have been treated across four different dose levels (60 mg/day [6 subjects], 80 mg/day [9 subjects], 120 mg/day [3 subjects], and 100 mg/day [3 subjects]). Two subjects dosed at 120 mg/day had DLTs (one with Grade 3 hypertension, one with Grade 3 dehydration) and required dose delay and reduction. 80mg once daily was determined to be the MTD on this study.

A total of 278 AEs have been reported for the 24 subjects enrolled in this ongoing Phase 1 dose-escalation study of daily administration of GSK1363089. Eighteen of 24 (75%) subjects reported at least one AE. Most (254/278, 91%) of the AEs reported were Grade 1 or 2 in severity, though a total of 17 Grade 3 and 2 Grade 4 events were reported. A total of 9/24 (37.5%) subjects had events of Grade 3 or higher. Grade 4 events were elevated GGT and pulmonary embolus, of which elevated GGT was considered GSK1363089-related. Grade 3 events were: dyspnea (2 subjects) and the following all occurring in one subject each with events not related unless noted: diarrhea (related), heparin-induced thrombocytopenia, perirectal abscess, post-procedural hemorrhage, blood alkaline phosphatase increased (related), blood calcium decreased (related), blood phosphorus decreased (related), electrocardiogram QT corrected interval prolonged, elevated GGT, increased lipase (related), dehydration (related), confusional state, renal failure (related), and hypertension (related). Many of these events were laboratory abnormalities without associated symptoms. A total of 100/278 (36%) events in 15 (63%) subjects were assessed as possibly or probably related to study treatment. Of the related events eight were Grade 3 and one was Grade 4 (GGT elevation).

Twenty one serious adverse events were reported in ten subjects. Four events in three subjects were assessed as possibly or probably related to study treatment including confusional state (Grade 3), dehydration (Grade 3), and hypertension (Grade 3) and elevated creatinine (Grade 2) in one subject.

A subject in Study XL880-002 taking 40 mg of GSK1363089 once a day experienced decreased left ventricular ejection fraction (LVEF) (INDSR# A0715996A). The investigator assessed the case as possibly related to GSK1363089. The event improved upon GSK1363089 discontinuation and medical intervention. The subject's medical history included possible pre-existing cardiomyopathy, prior use of chemotherapy with

potential cardiotoxicity and chest radiation. While decreased LVEF has been described for some other VEGFR TKIs i.e. Sutent (http://www.pfizer.com/files/products/uspi_sutent.pdf.), this event could be confounded by the patient's medical history.

There were three deaths in subjects on study XL880-002 which occurred within 30 days of the last dose of study drug. These were all assessed as not related to GSK1363089 treatment and attributed to sequelae of progressive disease. The events include: progression of metastatic colorectal cancer, progression of metastatic nasopharyngeal carcinoma, and acute respiratory failure.

Reasons for study drug discontinuation were known for a total of 14 of the 24 subjects (58%). One subject dosed at 60 mg withdrew from treatment due to an AE. The reasons for withdrawal of this subject include: sinus tachycardia, nausea, pneumonia, decreased appetite, dyspnoea, and pleural effusion. These adverse events were attributed by the investigator to the underlying process of heparin induced thrombocytopenia and subsequent multiple pulmonary emboli; all were considered not related to GSK1363089.

1.3.3.3.3. MET111644 (Formerly XL880-201)

A total of 105 AEs in 16 subjects have been reported as of 20 August 2007. Nine (56%) subjects reported at least one AE. The AEs, regardless of relationship with GSK1363089, reported in ≥10% of subjects are the following: hypertension (6 subjects, 38%); diarrhea, fatigue, headache, and nausea (each reported in 3 subjects, 19%); and dizziness, dysphonia, and vomiting (each reported in 2 subjects, 13%).

The majority (66 of 105) of AEs reported were of Grade 1. Nineteen events were of Grade 2, nine events were of Grade 3, two events were of Grade 4. No Grade 5 events were reported, and nine events were not assigned a grade. The Grade 3 events were: atrial flutter, pain in extremity, renal hemorrhage, dyspnea, pulmonary embolism, deep vein thrombosis (one event each), and three cases of hypertension. The two Grade 4 events were renal cell carcinoma and depression.

A total of 62 of the 105 events (59%) were considered possibly or probably related to GSK1363089. Eight of the 16 subjects (50%) reported at least one related event. The majority of these related events were of Grade 1 (45 of 62 events), 13 were of Grade 2, and three were of Grade 3. No Grade 4 or 5 related events were reported. Related AEs reported in ≥10% of subjects (2 subjects) were the following: hypertension (7 subjects,

44%), and diarrhea, fatigue, dizziness, and headache, in two subjects each (13%). The only Grade 3 events considered possibly or probably related to GSK1363089 were the events of hypertension in 3 subjects (19%). There were no AEs leading to withdrawals from the study.

Fifteen serious adverse events have been reported in three subjects as of 24 August 2007. Two events of vomiting in one subject were assessed as related to study drug GSK1363089.

One death from progressive papillary renal cell carcinoma that occurred within the 30 days from last administration of GSK1363089 was reported on MET111644. The event was assessed as not related to study treatment. In addition, one subject died 62 days after last study drug administration, following prior discontinuation from the study due to disease progression.

1.3.3.3.4. Ocular Events

As of 11 September 2008 a total of 193 subjects have been exposed to GSK1363089. A total of 5 ocular events have been reported in 5 subjects. All of these events occurred in subjects enrolled in study MET111644 for the treatment of Papillary Renal Carcinoma (PRC).

Three of these are vascular events including retinal vein occlusion, vitreous hemorrhage and optic ischemic neuropathy. These three subjects ranged in age from 49 to 65 years. The events occurred after 365 days, 7 days and 25 days of exposure to the drug, respectively. All of these events were confounded by the presence of hypertension and diabetes in the affected subjects. The events improved in all cases and GSK1363089 was reintroduced without further worsening.

Two subjects, 45 and 59 years old, developed a light-dark adaptation defect. These subjects had been on treatment with GSK1363089 for 308 and 447 days when the events occurred. The study drug was discontinued in the first subject due to disease progression, so the long term effect of continued GSK1363089 exposure on the defect is not known. The second subject, who was asymptomatic but had the defect discovered upon exam, remains on treatment, has stable status in relation to the light-dark adaptation defect, and is being closely monitored for any changes.

The overall prevalence of retinopathy in non-diabetic populations in the US is 7.7% (Cugati, 2006; Klein, 1994). The prevalence for the ocular events in the GSK1363089 program, regardless of causality, is 2.5% (5/193).

Although the prevalence of these events is low, these events have the potential to lead to significant vision loss. Hence, an ocular monitoring plan has been developed and will be implemented in all of the active clinical studies with GSK1363089, to provide additional safety data relating to ocular events (see Section 5.6.5). At the time of writing protocol amendment 4.0, the benefit-risk profile of GSK1363089 remains positive for subjects enrolled in the clinical program with PRC, gastric carcinoma and head and neck cancer.

1.3.3.4. GSK1363089 Clinical Pharmacokinetics

In XL880-001, the Phase 1 study of the Intermittent 5&9 dosing schedule, results from non-compartmental PK analysis indicated relatively rapid absorption of GSK1363089, with median peak concentrations occurring at about 4 hours after dose administration, independent of dose and PK study day. Over the 0.1 to 4.5 mg/kg dose range studied, AUC and Cmax increased in a roughly dose-proportional manner, particularly at doses greater than 1.4mg/kg (~100mg). Concentration-time profiles typically displayed monoexponential decay after attainment of Cmax. In the largest dose cohort (N=14) at the MTD dose of 3.6mg/kg (median 240mg), the median elimination t½ was 34 hours (range: 23 – 61 hours).

In XL880-002, the Phase 1 study using the once-daily dosing schedule, PK results are similar to those from XL880-001. Estimates of multiple-dose accumulation ratios based on pre-dose concentrations at various times during the first 28 days of dosing, the median elimination t½ at the MTD dose of 80mg once daily was 48 hours (range 22 – 72 hours).

1.3.3.5. GSK1363089 Clinical Observations in Study XL880-001

In Study XL880-001, efficacy is an exploratory objective; however, of the 40 subjects, there have been five subjects with partial responses (PRs): three subjects with papillary renal carcinomas and two subjects with thyroid carcinoma. Four additional subjects have had a decrease in tumor measurements not reaching the 30% Response Evaluation Criteria In Solid Tumors (RECIST), and seven other subjects have had stable disease (SD) for >3 months. In this population, these findings are suggestive of anti-tumor activity and warrant further investigation in more defined populations.

1.3.3.6. GSK1363089 Clinical Pharmacodynamics

Immunohistochemical analyses of tumor biopsies taken at baseline from three subjects in the XL880-001 clinical trial indicated high expression of c-MET and RON, with a high percentage (approximately 70%) of constitutively activated phospho-c-MET and phospho-RON. Increased labeling of phospho-ERK, phospho-Akt, and Ki67 (a marker of cell proliferation), and decreased apoptotic activity (assessed by terminal deoxynucleotide transferase-mediated dUTP nick-end labeling [TUNEL]), was also observed. In three subjects examined after treatment with GSK1363089 on an Intermittent 5&9 dosing schedule, a marked inhibition of phosphorylation of c-MET, RON, ERK, and Akt associated with a decrease in Ki67 and an increase in TUNEL labeling was observed. These effects were observed as early as after the first five daily doses and at doses as low as 0.8 mg/kg. These results suggest a decrease in proliferation and an increase in apoptosis in tumor cells after GSK1363089 treatment, consistent with the putative mechanism of action of GSK1363089. In one subject, two sets of biopsies were collected: at dosing Cycles 1 and 6. The magnitude of the effect observed after six treatment cycles was more pronounced than after one cycle, suggesting a cumulative effect over time.

1.4. Rationale

1.4.1. Rationale for the Study and Study Design

Gastric cancer remains one of the most common types of cancer in the world today. The clinical outcome for this malignancy remains poor, with a 5-year survival rate of only about 20% (all stages). The primary curative option in this clinical scenario is surgical excision. However, a significant unmet medical need exists for patients with unresectable gastric cancer because no effective therapy has yet been identified.

GSK1363089 is a new chemical entity that inhibits multiple RTKs with growth-promoting and angiogenic properties. The primary targets of GSK1363089 are the HGF and VEGF RTK families (e.g., c-MET VEGFR2/KDR, PDGFR).

Because amplification of *c-MET* has been associated with the pathogenesis of gastric cancer, inhibition of c-MET receptor activation by agents such as GSK1363089 may be of therapeutic benefit in this patient population.

1.4.2. Rationale for Dosage Selection

Subjects will be enrolled into one of two cohorts in this study:

- Cohort 1 – Intermittent 5&9 dosing; 240mg per dosing day;
- Cohort 2 – Daily dosing; 80mg per dosing day

The dose and schedule for Cohort 1 in this study are based on safety, PK, and pharmacodynamic data and clinical observations from the ongoing Phase 1 study, XL880-001, in which subjects are being dosed using the Intermittent 5&9 dosing regimen, Similarly, the dose and schedule for Cohort 2 in this study are based on safety, PK, and pharmacodynamic data and clinical observations from the ongoing Phase I study, XL880-002, in which subjects are being dosed using the daily dosing regimen.

Subjects in XL880-001 treated at nominal doses of up to 3.6 mg/kg have tolerated GSK1363089 well, with no DLTs. Adverse events that may potentially be associated with GSK1363089 administration (fatigue and hypertension) have been mild to moderate in severity, reversible, and managed with either additional medication (e.g., antihypertensives) or dose delay and/or reduction. Clinical signs (e.g., hypertension, proteinuria) and exploratory pharmacodynamic analyses suggest that modulation of VEGFR and/or c-MET likely occurs following administration of doses ≤3.6 mg/kg.

Based on preliminary PK analyses from XL880-001, fixed dosing is not expected to result in any greater variability in exposure than dosing based on body mass. In study XL880-001, subjects had nominal mass-based doses. Actual doses administered were based on the range into which an individual subject's weight fell. Weight ranges were <60 kg, ≥60 to <80 kg, ≥80 to <100 kg, and ≥100 kg. The dose for subjects weighing ≥60 to <80 kg in the 3.6-mg/kg dosing cohort was 240 mg per dosing day. Therefore, a regimen of 240 mg per dosing day administered on an Intermittent 5&9 dosing schedule is expected to be safe and potentially efficacious in subjects with metastatic gastric carcinoma or adenocarcinoma of the gastroesophageal junction or of the distal esophagus.

In the XL880-002 study, GSK1363089 was tolerated when administered for 28 days at doses up to 80 mg/day. Adverse effects include hypertension, fatigue, nausea, vomiting, and asymptomatic changes in laboratory values. The MTD for daily GSK1363089 dosing has been determined to be 80 mg/day. Based on preliminary PK analysis, GSK1363089 accumulates in plasma with repeated daily dosing. Results from pharmacodynamic analyses of plasma and surrogate tissue biomarkers indicate potential biological effects of

GSK1363089 in subjects. Anti-angiogenic pharmacodynamic marker changes have been detected in this clinical trial consistent with effects reported with other anti-angiogenic agents. The clinical observations of decreased tumor size, prolonged stable disease and/or improvement in tumor markers are consistent with the antitumor activity observed in XL880-001. These findings suggest that GSK1363089 dosed daily may have broad antitumor activity.

The 14-day MTD of GSK1363089 in both XL880-001 (Intermittent 5&9 regimen) and XL880-002 (daily regimen) is similar, with 1200mg (240mg x 5) and 1120mg (80mg x 14) administered over 14 days leading to very similar AUC(0-336h) of 14910 and 15110ng*hr/mL, respectively. Adding a cohort of subjects treated with the daily regimen will allow characterization of the safety, efficacy, and levels of exposure provided by two schedules of GSK1363089, as well as an exploration of whether the intermittent 5&9 regimen provides suboptimal exposure over time and whether continuous exposure provide better efficacy.

2. STUDY OBJECTIVES

The primary objectives of this study are as follows:

- To determine the confirmed response rate of GSK1363089 treatment in subjects with gastric carcinoma or adenocarcinoma of the gastroesophageal junction or of the distal esophagus.
- To further evaluate the safety and tolerability of GSK1363089.

The secondary objectives of this study are as follows:

- To assess the disease stabilization rate, progression-free survival, duration of stable disease, and overall survival in subjects with gastric carcinoma or adenocarcinoma of the gastroesophageal junction or of the distal esophagus who are treated with GSK1363089.
- To characterize the PK and pharmacodynamic parameters of GSK1363089 in subjects with gastric carcinoma or adenocarcinoma of the gastroesophageal junction or of the distal esophagus.

Note: Each of the primary and secondary study objectives will be assessed for each dosing Cohort (1 – Intermittent 5 days on/9 days off dosing; 2 – daily dosing) separately and together.

The exploratory objectives of this study are as follows:

- To characterize the *c-MET* amplification status of patients in this study population and possible associations with response to GSK1363089 therapy. In addition, the mutational status as well as expression of *c-MET* and related genes and proteins (e.g., RON) and of relevant downstream targets may also be assessed.
- To evaluate the efficacy and safety profiles between two GSK1363089 dosing regimens - Intermittent 5 days on/9 days off and daily.
- To evaluate the effect of GSK1363089 treatment on potential pharmacodynamic markers of clinical activity in paired tumor biopsies (e.g. phospho-MET).

3. STUDY DESIGN

3.1. Overview of Study Design

This is a Phase 2, non-randomized, sequential-cohort, open-label, single-stage, safety and efficacy study, with an initial cohort of subjects enrolled onto the Intermittent 5&9 regimen, and then a 2nd cohort of subjects enrolled onto the daily regimen. Enrolment into Cohort 2 will not begin until enrolment into Cohort 1 has completed.

The estimated length of the study is 40 months for subject accrual and treatment. This study consists of a Pre-Treatment Period (screening and baseline evaluations), a Study Treatment Period, an optional Treatment Extension Period, and a Post-Treatment Period (including an end-of-treatment visit and an extended follow-up period). Throughout the study, AEs will be recorded as volunteered. Adverse events will be graded according to the NCI CTCAE v3.0. A copy of this grading scale will be provided to the study sites upon request, or it can be accessed at http://ctep.cancer.gov/reporting/ctc.html. All doses of GSK1363089 are to be administered on an empty stomach.

3.1.1. Pre-Treatment Period

Subjects will undergo evaluations to determine study eligibility. Screening will be conducted within 28 days before initial treatment on Day 1. The following baseline evaluations must be done within 72 hours of the initial treatment: physical examination, measurement of vital signs, the Eastern Cooperative Oncology Group (ECOG) performance status (Appendix B), hematology, serum chemistry panel, urinalysis, and a pregnancy test (in women of childbearing potential). In addition, the appropriate arrangements must be made to ensure that 10 unstained slides or a paraffin block of archived tumor tissue are shipped to the central analysis laboratory, no later than 14 days after the subject’s first dose of study drug.

It is important to note that in the event that enrolment of 30 evaluable subjects in Cohort 2 is completed before the target of 4 subjects with *c-MET* amplification, as defined by ≥2 copies of *c-MET* per copy of chromosome 7 (see Section 3.3.1 for full definition), has been reached, then extra subjects, with proven *c-MET* amplification, pre-screened by FISH, will be enrolled in the study, until the target is reached. In this instance, 10 unstained slides or a paraffin block of tumor tissue must be shipped to the central analysis laboratory as soon as possible after the subject signs the consent form. The subject will not be able to start the study treatment period until FISH results are available, and then only if they have *c-MET*-amplified tumor, as defined by ≥2 copies of *c-MET* per copy of chromosome 7.

3.1.2. Study Treatment Period

Treatment will be administered on an outpatient basis. Previously reported AEs and potential risks for GSK1363089 are described in Section 5.11. Dose modifications for GSK1363089 are described in Section 3.4. No investigational or commercial agents or therapies other than those described in Section 4.4 may be administered with the intent to treat a subject's malignancy.

GSK1363089 will be administered at a dose of 240 mg on an Intermittent 5&9 dosing schedule in Cohort 1; and at a dose of 80 mg on a daily dosing schedule in Cohort 2. Each treatment cycle will be 2 weeks in duration. The 8-week Study Treatment Period will consist of four 2-week treatment cycles. Laboratory evaluations and physical examinations will be conducted before each dosing cycle. GSK1363089 treatment will be stopped if there is disease progression at any time.

In-patient hospitalization is not required for study treatment and assessments if the subject is adequately observed and procedures are performed as specified herein.

At specified timepoints during the treatment period, AEs will be solicited by asking the subject, "Are you having any new symptoms?" Answers will be recorded on the AE case report form (CRF) page. Tumor assessment will be performed at the end of the Study Treatment Period (8 weeks).

3.1.3. Treatment Extension Period

In the absence of progressive disease (PD) and unacceptable toxicity, subjects may receive GSK1363089 treatment on the same dosing schedule for up to a total of 2 years at

the discretion of the investigator and beyond 2 years with the approval of the sponsor while participating in this study. Tumor assessment will continue approximately every 8 weeks while the subject remains on study.

3.1.4. Post-Treatment Period

Subjects will return to the study site 30 (±4) days after the last dose of study drug to complete end-of-treatment assessments. On Days 90 and 180 (±15 days) after the last dose of study drug or until death (if before Day 180), the investigator (or designee) will obtain follow-up information including subsequent cancer treatments, any serious adverse events (SAEs) considered to be possibly or probably related to study drug, death, and date and cause of death.

3.2. Study Sites

It is anticipated that this study will be conducted at up to 20 clinical sites, mostly in the United States. For the purposes of this protocol, sites using satellite facilities will be counted as one site.

3.3. Treatment Assignment

It is the responsibility of the investigator to contact GSK (or designee) for a subject number before treating each subject with GSK1363089. GSK (or designee) will have records of the number of subjects treated.

3.3.1. Treatment Groups

Initially, subjects with a histologic diagnosis of poorly–differentiated, advanced or metastatic gastric carcinoma or adenocarcinoma of the gastroesophageal junction will be eligible for enrollment into Cohort 1 (Intermittent 5&9 dosing), until a total of 30 subjects is determined to be evaluable (see Section 8.1.1 for definition of ‘evaluable’). After entry, subjects will be stratified retrospectively into one of the two strata listed below based on the presence of confirmed *c-MET* amplification at 7q31, as defined as at least 3 genomic copies of the *c-MET* gene determined by FISH:

- Stratum A: Subjects with confirmed *c-MET* amplification at 7q31.
- Stratum B: Subjects without confirmed *c-MET* amplification at 7q31.

If fewer than eight of those 30 subjects have amplification of *c-MET,* as defined as ≥3 genomic copies of the *c-MET* gene (Stratum A), up to 10 additional, evaluable subjects will be enrolled to better characterize the effect of GSK1363089 in this subgroup, with a goal of attaining eight evaluable subjects who have amplification of *c-MET,* as defined as ≥3 genomic copies of the *c-MET* gene.

After Cohort 1 has completed enrolment, subjects with a histologic diagnosis of advanced or metastatic gastric carcinoma or adenocarcinoma of the gastroesophageal junction or of the distal esophagus will be eligible for enrollment into Cohort 2. This will include subjects with well- and moderately-differentiated tumors, as well as poorly-differentiated tumors. Enrolment into Cohort 2 will continue until a total of 30 subjects are determined to be evaluable. After entry, subjects will be stratified retrospectively into one of the two strata listed below based on the presence of confirmed *c-MET* amplification at 7q31, as defined as at least 3 genomic copies of the *c-MET* gene:

- Stratum A: Subjects with confirmed *c-MET* amplification at 7q31.
- Stratum B: Subjects without confirmed *c-MET* amplification at 7q31.

If fewer than 8 of the evaluable subjects in Cohort 2 have a *c-MET* amplification, as defined as ≥3 genomic copies of the *c-MET* gene, then up to 10 extra evaluable subjects will be enrolled, in order to have at least 8 *c-MET*-amplified subjects in this Cohort, as defined as ≥3 genomic copies of the *c-MET* gene.

In addition, as part of Amendment 4.0, subjects in both cohorts will also be stratified according to a more stringent definition of *c-MET* gene amplification (i.e. ≥2 copies of *c-MET* per copy of chromosome 7). This is because in an analysis of an initial group of Cohort 1 subjects whose tumors had "at least 3 genomic copies of the *c-MET* gene", a number of them did not have true *c-MET*-gene amplification (i.e. ≥2 copies of *c-MET* per data from Smolen *et al*, gastric tumor cell lines with true *c-MET*-amplification, consistent with so-called homogeneously staining regions (HSRs), are significantly more sensitive to a specific c-MET tyrosine kinase inhibitor (PHA-665752) than cell lines with no- or low-level-copy number increase, such as those with chromosome 7 aneuploidy (Smolen, 2005).

Therefore, in addition to the definition of *c-MET* amplification used in this protocol up to Amendment 3.0 (i.e. at least 3 genomic copies of the *c-MET* gene), a more stringent

definition will also be used – i.e. those who meet at least one of the following criteria upon FISH analysis:

1. Ratio of the average copy numbers of *c-MET* to *CEP 7,* across at least 60 cells, of at least two;
2. Any cells containing multiple copies of the *c-MET* gene in homogeneously-staining regions (HSRs)

Note - as part of Amendment 4.0, the 2 definitions of *c-MET*-amplification referred to in this section will be written as follows:

- The original definition will be written as "*c-MET* amplification, as defined as ≥3 copies of the *c-MET* gene"
- The new definition, described above, will be written as "*c-MET* amplification, as defined as ≥2 copies of the *c-MET* gene per copy of chromosome 7"

In Cohort 2, in addition to requiring at least 8 evaluable subjects to have *c-MET* amplification, as defined as ≥3 copies of the *c-MET* gene, there will also be a requirement to have at least 4 evaluable subjects who meet the more stringent definition of *c-MET* gene amplification, as defined as ≥2 copies of the *c-MET* gene per copy of chromosome 7.

In the event that the enrolment of 30 evaluable subjects in Cohort 2 is completed before the target of 4 subjects with *c-MET* amplification (as defined as ≥2 copies of the *c-MET* gene per copy of chromosome 7) has been reached, then extra *c-MET*-amplified subjects (as defined as ≥2 copies of the *c-MET* gene per copy of chromosome 7), identified by pre-screening FISH, will be enrolled in the study, until the target is reached.

All subjects will have their diagnosis of gastric cancer confirmed by independent central pathology review (see Section 5.9.4).

3.4. Dose Reduction or Treatment Delay for Toxicity

For the purpose of this protocol and for assistance with making decisions regarding dose reduction or delays, the following CTCAE Grade 3 definitions have been slightly modified (modifications are in italics):

- Grade 3 hypertension *despite optimal antihypertensive therapy with one or more drugs* (see Management of Treatment-Emergent Hypertension for information regarding management of treatment-emergent hypertension)
- Grade 3 nausea, vomiting, or diarrhea *despite prophylaxis and/or treatment* (see Section 4.4.2 for information regarding supportive care treatment)

In the event of treatment emergent hepatotoxicity, irrespective of whether the investigator considers it related to study drug, the management guidelines described in Section 4.4.4 should be followed.

If a subject experiences a new onset of unacceptable symptoms or signs of toxicity (excluding hepatotoxicity which is covered above), but in the investigator's opinion should continue to receive study drug, study treatment may be delayed for up to 21 days, then resumed either at the same dose or at a reduced dose, according to the criteria set out in Sections 3.4.1 and 3.4.2. The details of how to dose-reduce subjects are set out in Sections 3.4.3 and 3.4.4.

3.4.1. Symptoms/Signs Unrelated to Study Drug

If unacceptable symptoms or signs of toxicity are considered unrelated to study drug and resolve within 21 days, the subject may resume study treatment at his or her current dose level.

If unacceptable symptoms or signs of toxicity are considered unrelated to GSK1363089 but have not resolved to Grade ≤1 or to within 10% of baseline values within 21 days, the subject will receive no further doses of GSK1363089 and will enter the Post-Treatment Period.

3.4.2. Symptoms/Signs Related to Study Drug

If unacceptable study drug-related symptoms resolve within 21 days, the subject may resume study treatment with a reduction in dose.

If, after 21 days, study drug-related symptoms or signs of toxicity do not resolve to Grade ≤1 or to within 10% of baseline values, the subject will receive no further doses of GSK1363089 and will enter the Post-Treatment Period.

3.4.3. Dose Reduction in Cohort 1 (Intermittent 5&9 Dosing)

In Cohort 1, GSK1363089 will be dosed at 240 mg/day on an Intermittent 5&9 dosing schedule. If a single dose reduction is required, the reduction will be to 160 mg/day; if a second dose reduction is required, the reduction will be to 100 mg/day. If a subject requires dose reduction more than 2 times because of unacceptable study drug-related AEs, the subject's continuation on the study must be discussed with and agreed to by the GSK study physician.

3.4.4. Dose Reduction in Cohort 2 (Daily Dosing)

In Cohort 2, GSK1363089 will be dosed at 80 mg/day on a daily dosing schedule. If a single dose reduction is required, the reduction will be to 60 mg/day. If toxicities occur while on the 60 mg/day dosing schedule, further modifications to the schedule will depend on how long the subject has been on 60 mg/day dosing, as follows:

- If toxicities occur after 3 or more weeks on 60 mg/day dosing, then a 1 week treatment rest should be instituted, such that a new schedule of 3 weeks on/1 week off is implemented. If 60 mg/day dosing is still not tolerable on the 3 week on / 1 week off schedule, the subject's continuation on the study, in addition to any further modifications to the dosing schedule, must be discussed with and agreed to by the GSK study physician.
- If toxicities occur after less than 3 weeks on 60 mg/day dosing, the subject's continuation on the study, in addition to any further modifications to the dosing schedule, must be discussed with and agreed to by the GSK study physician.

3.5. Requirement for Collection of Paired Tumor Biopsies

Following the implementation of protocol Amendment 4.0, the collection of fresh, paired tumor biopsies will become a requirement rather than an option, to allow the assessment of the expression of pharmacodynamic markers of GSK1363089 activity, in particular relating to the c-MET pathway (e.g. total c-MET, phospho-MET and downstream markers).

After the first 6 subjects have been enrolled under Amendment 4.0 and have had paired tumor biopsies collected, enrolment will be put on temporary hold, while the safety and acceptability of collecting paired biopsies, plus the pharmacodynamic data, are reviewed

by GSK. If there are no safety concerns, and the pharmacodynamic objective with respect to c-MET has not been met, then subject enrolment will recommence and continue until the target number of subjects for Cohort 2 has been reached.

The safety and pharmacodynamic data for the paired tumor biopsies will continue to be reviewed on an ongoing basis. If a safety concern with obtaining the biopsies should ever arise and/or the pharmacodynamic objective with respect to c-MET has been met, then paired biopsies will no longer be required in this study. This will be communicated to the sites and IRBs by letter from GSK or its designee.

If any subject is not able to provide the second biopsy (i.e. at Day 5-8), then that subject will be allowed to continue on study and will be eligible for inclusion in the safety and efficacy assessments. However, if by so doing, this would prevent at least 12 subjects being enrolled who do provide paired biopsies, then an extra subject will be enrolled.

Instructions for the collection of paired tumor biopsies are provided in Section 5.9.2 and the Pharmacodynamic Laboratory Manual.

4. STUDY POPULATION

4.1. Target Population

This study will be conducted in subjects with metastatic gastric carcinoma.

4.2. Inclusion Criteria

A subject must meet the following criteria to be eligible for the study:

1. The subject has a histologically confirmed diagnosis of advanced or metastatic gastric carcinoma, or adenocarcinoma of the gastroesophageal junction or of the distal esophagus. Subjects with tumors of the gastroesophageal junction or of the distal esophagus may be eligible provided that the tumor is not of squamous or sarcomatous histology.
2. The subject has measurable disease, defined as at least one lesion that can be accurately measured in at least one dimension (longest diameter to be recorded) as ≥20 mm with conventional techniques or as ≥10 mm with spiral computerized tomography (CT) scan.

3. Ten unstained slides of representative tumor tissue or a paraffin block of tumor, archival or recently-prepared, have been identified for shipment to the central analysis laboratory. These samples will be used initially for determining the *c-MET*-amplification status by FISH.
4. The subject consents to provide paired tumor biopsies, directly prior to commencing study treatment and then between Days 5 and 8. These samples will be used for analysis of pharmacodynamic markers of GSK1363089 activity; they are in addition to the tumor tissue described in Inclusion Criterion 3.
5. The subject is at least 18 years old.
6. The subject has an ECOG performance status ≤2.
7. The subject is able to ingest the GSK1363089 capsules.
8. In the adrenocorticotropic hormone (ACTH) stimulation test, the subject has a serum cortisol level ≥20 µg/dL (552 nmol/L) 30-90 minutes after injection of ACTH.
9. The subject has organ and marrow function as follows: absolute neutrophil count (ANC) ≥1500/mm^3, platelets ≥100,000/mm^3, hemoglobin ≥9 g/dL, bilirubin ≤1.5 mg/dL or ≤1.5 x ULN if no liver involvement (or ≤2 mg/dL or ≤2 x ULN with liver involvement); serum creatinine ≤1.5 mg/dL or calculated creatinine clearance ≥60 mL/min, ALT and AST ≤2.5 x ULN if no liver involvement or ≤5 x ULN with liver involvement.
10. The subject is capable of understanding and complying with the protocol and has signed the informed consent document.
11. Sexually active subjects (male and female) must use a medically-accepted method of contraception during the course of the study.
12. Female subjects of childbearing potential must have a negative serum pregnancy test at screening.
13. The subject has had no other diagnosis of malignancy (unless non-melanoma skin cancer or a malignancy diagnosed ≥5 years ago, and has no evidence of disease for 5 years prior to the screening for this study).
14. The subject has a QTcB (Bazett corrected) or QTcF (Frederica corrected) < 470 msec. If the first ECG reading is ≥470 msec, repeat the ECG twice over a 10 minute period. Then use the average QTc value to determine whether the subject can be included.

4.3. Exclusion Criteria

A subject who meets any of the following criteria is ineligible for the study:

1. The subject has received more than two lines of prior cytotoxic chemotherapy for locally advanced or metastatic disease. For the purpose of this protocol, neoadjuvant therapy would not be considered to be prior cytotoxic chemotherapy. In addition, potential subjects who have received prior treatment with c-MET signaling inhibitor (e.g. Small molecule c-MET inhibitors and/or antibodies against HGF) are excluded.
2. The subject has received an investigational drug within 14 days of the first dose of study drug.
3. The subject has received chemotherapy, immunotherapy, or radiation therapy (to ≥25% of his or her bone marrow) within 14 days or has received nitrosoureas or mitomycin C within 6 weeks prior to the scheduled first dose of GSK1363089.
4. The subject has AEs due to investigational drugs or other medications administered more than 21 days prior to enrollment that have not recovered to Grade ≤1 using NCI CTCAE v3.0, with the exception of alopecia greater than grade 1.
5. The subject has known brain metastases.
6. The subject has uncontrolled intercurrent illness including, but not limited to, ongoing or active infection, symptomatic congestive heart failure, unstable angina pectoris, cardiac arrhythmia, or psychiatric illness/social situations that would limit compliance with study requirements.
7. The subject is pregnant or breastfeeding.
8. The subject is known to be positive for the human immunodeficiency virus (HIV).
9. The subject has a previously identified allergy or hypersensitivity to components of the GSK1363089 formulation.
10. The subject is unable or unwilling to abide by the study protocol or cooperate fully with the investigator or designee.

4.4. Concomitant Medication and Treatment

If the subject must use a concomitant medication during the study, it is the responsibility of the principal investigator (PI) to ensure that details regarding the medication are recorded on the CRF.

4.4.1. Anticancer Treatment

If a subject requires additional anticancer treatment (e.g., chemotherapy, radiation therapy, or surgery), he or she will not be able to continue with GSK1363089 treatment and will enter the Post-Treatment Period (end-of-treatment visit/follow-up). Palliative radiotherapy is allowed during the study only in the event that the lesion to be radiated

was recognized prior to study entry, and neither the presence of the lesion nor the need for its radiation represent PD.

4.4.2. Supportive Care

Antiemetics and antidiarrheal medications will not be administered prophylactically before initial treatment with study drug. At the discretion of the investigator, prophylactic use of antiemetic and antidiarrheal medication may be used as per standard clinical practice before subsequent doses of study drug.

Colony-stimulating factors (e.g., erythropoietin and granulocyte colony stimulating factor) and pain medications administered as dictated by standard clinical practice are acceptable while the subject is enrolled in the study.

4.4.3. Management of Treatment-Emergent Hypertension

Subjects may develop hypertension as a result of GSK1363089 treatment. It is recommended that hypertension is addressed prior to treatment initiation. Table 2 provides a recommended algorithm for hypertension management. Decisions to hold or decrease the GSK1363089 dose during treatment must be based on BP readings taken by a medical professional and must be confirmed with a second measurement at least 5 minutes following the first measurement. While subjects are receiving treatment with GSK1363089, the early initiation of antihypertensive treatment for Grade 1 or 2 hypertension to minimize more severe or persistent hypertension is not considered a Grade 3 AE.

Table 2 Management of Treatment-Emergent Hypertension

BP Measurements - Systolic/Diastolic	Treatment/Dose Modification
**Subjects not receiving maximal antihypertensive therapy[a]**	
>140 mmHg (systolic)<br>OR<br>>90 mmHg (diastolic)<br>OR<br>Increase of diastolic BP by ≥20 mmHg regardless of baseline BP	• Add new or additional antihypertensive medications or increase dose of existing medications.<br>• Maintain dose of GSK1363089.
>180 mmHg (systolic)<br>OR<br>>105 mmHg (diastolic)	• Hold GSK1363089.<br>• Add new or additional antihypertensive medications or increase dose of existing medications.<br>• Monitor subject closely for hypotension (if on antihypertensive meds) until GSK1363089 is restarted.<br>• Resume treatment at same dose level when BP falls to <140/90.
**Subjects receiving maximal antihypertensive therapy[a]**	
>160 mmHg (systolic)<br>OR<br>>105 mmHg (diastolic)	• Hold GSK1363089.<br>• Maintain antihypertensive medications and monitor subject closely for hypotension until GSK1363089 is restarted.<br>• Resume treatment at one lower dose level when BP falls to <140/90 (see Section 3.4 for details of dose reduction).

[a] Maximal antihypertensive therapy is defined as four antihypertensive medications given for 2 weeks.

4.4.4. Management of Treatment-Emergent Hepatotoxicity

In the event of treatment-emergent hepatotoxicity, potential contributing factors such as concomitant medications, viral hepatitis, choledocholithiasis, and hepatic metastases should be investigated. Concomitant medications known to be hepatotoxic which may be contributing to liver dysfunction should be discontinued or replaced with alternative medications to allow for recovery of liver function. As generally understood, ALT >3 x ULN and concomitant bilirubin ≥2.0 x ULN (>35% direct bilirubin), in the absence of elevated alkaline phosphatase or biliary injury, suggests significant liver injury.

Table 3 shows the liver-enzyme entry criteria, split by whether there is liver involvement.

Table 3 Liver Enzyme Entry Criteria

	No liver involvement	With liver involvement
**ALT and AST**	≤2.5 x ULN	≤5 x ULN
**Total bilirubin**	≤1.5 mg/dL or ≤1.5 x ULN	≤2 mg/dL or ≤2 x ULN

Liver chemistry stopping and follow-up criteria have been designed to ensure subject safety and evaluate liver event etiology. Appendix G provides a flow diagram of these criteria.

Therapy with GSK1363089 should be terminated if any of the following criteria are met:

11. ALT ≥3 × ULN or ≥3 × Baseline (if baseline ALT >ULN) **and** bilirubin ≥1.5 × ULN or ≥1.5 × Baseline (if baseline bilirubin >ULN **and** >35% direct bilirubin)
12. ALT ≥5 × ULN or ≥5 × Baseline (if baseline ALT >ULN)
13. ALT ≥3 × ULN or ≥3 × Baseline (if baseline ALT >ULN) if associated with the appearance or worsening of hepatitis symptoms
14. ALT ≥3 × ULN or ≥3 × Baseline (if baseline ALT >ULN) that persists for ≥4 weeks

Baseline ALT and total bilirubin should be calculated as the average of **all** liver chemistry results obtained from Screening through Day 1. This requirement is based on the variability of baseline and screening ALT values observed in an earlier study.

When any of the liver chemistry stopping criteria is met, do the following:

- **Immediately withdraw GSK1363089**
- Report the event to GSK within **24 hours** of learning its occurrence
- Complete the liver event CRF
- Complete the SAE data collection tool if the event also meets the criteria for an SAE
- Perform liver imaging (CT scan or MRI) within 7 days
- Complete the liver imaging and/or liver biopsy CRFs if these tests are performed
- Perform liver event follow-up assessments, and monitor the subject until liver chemistries resolve, stabilize, or return to baseline values as described below
- Withdraw the subject from the study after completion of the liver chemistry and viral studies and resolution of liver function to baseline levels
- Do not rechallenge with investigational product

In addition, for criterion 1:

- Make every reasonable attempt to have subjects return to clinic within **24 hours** for repeat liver chemistries, liver event follow-up assessments (see below), and close monitoring
- A specialist or hepatology consultation is recommended
- Monitor subjects twice weekly until liver chemistries (ALT, AST, alkaline phosphatase, bilirubin), complete blood count, platelet count, and prothrombin time resolve, stabilize or return to within baseline values
- Measure anti-nuclear antibody, anti-smooth muscle antibody, and Type 1 anti-liver-kidney microsomal antibodies
- Liver biopsy may be performed at the discretion of the investigator

For all other criteria:

- Make every reasonable attempt to have subjects return to clinic **within 24 to 72 hours** for repeat liver chemistries and liver event follow-up assessments (see below)
- Monitor subjects weekly until liver chemistries (ALT, AST, alkaline phosphatase, bilirubin) resolve, stabilize or return to within baseline values

Subjects with ALT ≥3 × ULN **but** <5 × ULN or ≥3 × Baseline (if baseline ALT >ULN) **but** <5 × Baseline (if baseline ALT >ULN) **and** bilirubin <1.5 × ULN or <1.5 × Baseline (if baseline bilirubin >ULN), without hepatitis symptoms or rash, and who can be monitored weekly for 4 weeks:

- Can continue investigational product
- Must return weekly for repeat liver chemistries (ALT, AST, alkaline phosphatase, bilirubin), complete blood count, platelet count, and prothrombin time until they resolve, stabilize or return to within baseline values
- If at any time these subjects meet the liver chemistry stopping criteria, proceed as described above
- If, after 4 weeks of monitoring, ALT <3 × ULN **or** <3 × Baseline (if baseline ALT ≥ULN) and bilirubin <1.5 × ULN **or** <1.5 × Baseline (if baseline bilirubin >ULN), monitor subjects twice monthly until liver chemistries normalize or return to within baseline values

If any of the stopping criteria is met, make every attempt to carry out the **liver event follow-up assessments** described below:

- Viral hepatitis serology including:
  - Hepatitis A immunoglobulin M (IgM) antibody

Hepatitis B surface antigen and hepatitis B core antibody (IgM)

Hepatitis C RNA

Cytomegalovirus IgM antibody

Epstein-Barr viral capsid antigen IgM antibody (or if unavailable, obtain heterophile antibody or monospot testing)

Hepatitis E IgM antibody (if subject resides outside the United States or Canada, or has traveled outside United States or Canada in past 3 months)

- Blood sample (3 mL) for PK analysis, obtained within 72 hours (preferably within 24 hours) of last dose. Record the date/time of the PK blood sample draw and the date/time and amount of the last dose of investigational product before blood sample draw on the CRF. If the date or time of the last dose is unclear, provide the subject's best approximation. If the date/time of the last dose cannot be approximated OR a PK sample cannot be collected in the time period indicated above, do not obtain a PK sample. Instructions for sample handling and shipping are in the PK Laboratory Manual.
- Serum creatine phosphokinase and LDH
- Obtain complete blood count with differential to assess eosinophilia
- Record the appearance or worsening of clinical symptoms of hepatitis, or hypersensitivity, fatigue, decreased appetite, nausea, vomiting, abdominal pain, jaundice, fever, or rash as relevant on the AE CRF
- Record use of concomitant medications, acetaminophen, herbal remedies, other over-the-counter medications, or putative hepatotoxins, on the concomitant medications CRF
- Record alcohol use on the liver event alcohol intake CRF

4.5. Potential Drug Interactions

Drug interaction studies have not been conducted with GSK1363089 in humans. However, *in vitro* studies indicate NADPH-dependent liver microsomal oxidative metabolism of GSK1363089 by CYP3A4. Therefore, use of drugs that are inhibitors or inducers of CYP3A4 should be avoided in subjects who are receiving GSK1363089 (e.g., 3A4 inhibitors ketoconazole, HIV protease inhibitors, diltiazem, and verapamil and 3A4 inducers phenobarbital, phenytoin, and rifampin).

In vitro, GSK1363089 is a moderate inhibitor of hepatic microsomal isozymes cytochrome P450 (CYP): CYP3A4, CYP2C8, CYP2C9, and CYP2D6. Therefore, use of drugs that are substrates of the liver enzymes CYP3A4 (e.g., triazolobenzo‑diazepines, dihydropyrolidine calcium channel blockers, certain HMG‑CoA reductase inhibitors),

CYP2D6 (e.g., tricyclic antidepressants, venlafaxine, fluoxetine, paroxetine, beta blockers, and codeine), CYP2C8 (e.g., repaglinide, cerivastatin, torsemide), and CYP2C9 (e.g., phenytoin, warfarin, and non-steroidal anti-inflammatory drugs) should be avoided in subjects who are receiving GSK1363089 unless deemed clinically necessary (i.e. no non-P450 interacting alternative is available) and the subject can be monitored for the desired drug effect or level and for potential adverse effects. See Appendix D for a more comprehensive list of drugs that inhibit/induce CYP3A4, or are a substrate for CYP3A4, CYP2D6, CYP2C8, or CYP2C9.

GSK1363089 is highly protein bound, and other drugs that are also highly protein bound should be used with caution and may require more frequent monitoring of effects. Examples of such agents include warfarin, diazepam, furosemide, dicloxacillin, propanolol, and phenytoin.

5. STUDY ASSESSMENTS AND PROCEDURES

5.1. Overview

The study consists of the following procedures:

1. **Pre-Treatment Period**: The screening period must occur within 28 days of dosing. The screening visit may be counted as the baseline visit if completed within 72 hours of dosing.

2. **Study Treatment Period:** In Cohort 1, GSK1363089 will be administered at 240 mg per dosing day as capsules. Each treatment cycle will be 2 weeks in duration and will consist of an Intermittent 5&9 dosing schedule. In Cohort 2, GSK1363089 will be administered at 80 mg per dosing day as capsules on a daily dosing schedule. The 8-week Study Treatment Period will consist of four 2-week treatment cycles. GSK1363089 treatment will be stopped if a subject has disease progression.

3. **Treatment Extension Period:** In the absence of PD and unacceptable toxicity, subjects may receive GSK1363089 treatment on the same dosing schedule for up to a total of 2 years at the discretion of the investigator and beyond 2 years with the approval of the sponsor while participating in this study.

4. **Post-Treatment Period:** Subjects will return to the study site 30 (±4) days after the last dose of GSK1363089 for an end-of-treatment assessment. On Days 90 and 180 (±15 days) after the last dose of GSK1363089 or until death (if before Day 180), the investigator (or designee) will obtain follow-up information.

In the absence of toxicity, all scheduled visits should occur within ±3 days of the protocol-specified visit schedule. If the subject experiences toxicity, study treatment may be adjusted or delayed at the discretion of the investigator (see Section 3.4 for toxicity-related delays and/or dose reductions; see Section 4.4.3 for information regarding management of GSK1363089-related treatment-emergent hypertension). The study assessments are presented in Appendix A.

Sites that are unable to perform the PK or pharmacodynamic sample collection as specified will not be permitted to participate in the study.

5.2. Pre-Treatment Period

Informed consent must be obtained before any study-specific tests or evaluations are conducted.

The screening visit will occur within 28 days before administration of study drug and may serve as the baseline evaluations if done within 72 hours of initial dosing. Study eligibility will be based on fulfilling all study inclusion and exclusion criteria. The investigator will use clinical judgment when determining the clinical significance of laboratory values at baseline and throughout the study.

At the screening visit, information will be collected and subjects will have clinical evaluations as follows:

- Informed consent
- Demographics
- Medical and cancer history
- Physical examination, including measurements of height and weight
- Vital signs (5-minute sitting BP, pulse, respiratory rate, oral temperature)
- ECOG performance status assessment (Appendix C)
- Prothrombin (PT)/partial thromboplastin (PTT) times or International Normalized Ratio (INR)

- Clinical laboratory tests (hematology, serum chemistry panel, urinalysis) (see Section 5.6)
- 12-Lead electrocardiogram (ECG) (see Section 5.6.6)
- If a subject presents with abnormal ECGs or clinical symptoms, either or both of which are suggestive of a decreased ejection fraction, then he or she is to be evaluated by cardiac imaging (ECHO or MUGA) for determination of cardiac function prior to enrolment. The results should be discussed with the GSK Study Physician to decide whether the subject can be enrolled
- ACTH stimulation test (see Section 5.6.3)
- Thyroid function tests (TSH, Free T3 and T4) (see Section 5.6.4)
- Ocular assessments – the following examinations must be performed in all subjects (see Section 5.6.5):
    - Best corrected visual acuity
    - Goldman visual field evaluation
    - Measurement of intraocular pressure
    - Slit lamp examination
    - Dilated fundoscopic examination including full field color retinal photographs
- Ocular assessments – the following examinations must be performed in subjects who report poor vision in low illumination or have other symptoms suggestive of night blindness (see Section 5.6.5):
    - Light-dark adaptation testing
    - International Society for Clinical Electrophysiology of Vision (ISCEV) standard Electroretinography (ERG)
    - Cancer-associated retinopathy (CAR) antibody testing
- Radiologic tumor assessment by CT-scans or MRI of chest, abdomen and pelvis; additional imaging is allowed if clinically indicated. Imaging may be performed anytime within 21 days before the first dose of GSK1363089. (see Section 5.9.3)
- Pregnancy test (serum) in women of childbearing potential
- Concomitant medications
- Collection of both of the following sets of tumor samples:
    1. Ten unstained slides of representative, tumor tissue or a paraffin block of tumor tissue for assessment of status of *c-MET* amplification at 7q31 (see Section 5.9.1). Note, these samples can be archival or recently-prepared prior to the study. Samples should be shipped to the GSK-designated central laboratory no later than 14 days after the subject's first dose of GSK1363089. Note – in the event that additional subjects are required, (pre-screened for *c-MET* amplification, as defined as ≥2 copies of *c-MET* per copy of chromosome 7), then tumor samples must be shipped to the central laboratory, and the *c-MET* amplification status demonstrated by FISH, prior to commencing the Study Treatment period.
    2. In addition to the collection of archived tissue for determining *c-MET*-amplification status, fresh, paired tumor biopsy samples for pharmacodynamic

assessment of GSK1363089's clinical activity, the pre-treatment, baseline tumor biopsy sample will be collected as soon as possible **prior** to commencing study treatment, but at least 14 days after the completion of any prior anti-cancer therapy (see Section 5.9.2). Note, this tumor biopsy must not be performed until all inclusion and exclusion criteria have been met. The tumor biopsy must be frozen immediately, per the instructions in the Pharmacodynamic Laboratory Manual. This sample must **not** be used to provide slides for *c-MET* amplification testing without first consulting the GSK Study Physician.

If screening evaluations are not done within 72 hours of the initial dose of GSK1363089, the following evaluations will be repeated at a baseline visit:

- Physical examination
- Vital signs (5-minute sitting BP, pulse, respiratory rate, oral temperature)
- ECOG performance status assessment (Appendix C)
- Clinical laboratory tests (hematology, serum chemistry panel, urinalysis) (see Section 5.6)
- Pregnancy test (urine or serum) in women of childbearing potential
- Concomitant medications

Note: If performed within 24 hours of the first dose of GSK1363089 on Day 1, baseline evaluations may serve as the pre-dose Day 1 visit evaluations. Test results must be available before study drug administration (no more than 72 hours for the ACTH results) with the exception of thyroid function tests.

5.3. Study Treatment Period

Study drug will be administered at the study site at each protocol-defined visit during the Study Treatment Period; other doses will be self-administered.

5.3.1. Week 1, Day 1 — First Dose of Study Drug

5.3.1.1. Pre-Dose Evaluations

- Symptom-directed physical examination (complete the portion[s] of the physical examination relevant to subject symptoms) and weight
- Vital signs (5-minute sitting BP, pulse, respiratory rate, oral temperature)
- ECOG performance status assessment (Appendix C)
- 12-Lead ECG (see Section 5.6.6)
- Pregnancy test (urine or serum) in women of childbearing potential
- PK and pharmacodynamic blood samples collected within approximately 30 minutes pre-dose (see Section 5.7)

Note: The physical examination, weight measurement, ECOG performance status, clinical laboratory tests, and pregnancy test may be performed up to 24 hours before dosing with study drug; test results must be available before study drug administration.

5.3.1.2. Administration of GSK1363089

GSK1363089 should be administered at approximately the same time each day.

After a 2-hour fasting period, subjects will receive GSK1363089. Subjects should drink a full glass of water (approximately 240 cc or 8 oz.) immediately following GSK1363089 administration. Subjects will continue to fast for 1 hour after study drug administration.

5.3.1.3. Post-Dose Evaluations

- Vital signs (5-minute sitting BP, pulse, respiratory rate, oral temperature) taken within approximately 30 minutes before the PK sampling timepoint
- PK and pharmacodynamic blood samples collected at 4 hours post-dose (see Section 5.7)
- Concomitant medications
- Adverse events (see Section 7.1.1)

5.3.2. Week 1, Day 5-8 (Fifth-Eighth Dose of Study Drug)

The second fresh tumor biopsy will be collected preferably from the same organ as the baseline tumor biopsy. It is highly recommended to collect this tumor biopsy on Day 5; however, it is also acceptable to collect it as late as Day 8 (see Section 5.9.2 and the Pharmacodynamic Laboratory Manual).

5.3.3. Week 1, Day 5 (Fifth-Eighth Dose of Study Drug) For Intermittent 5&9 Regimen; Week 2, Day 8 (Eighth Dose of Study Drug) For Daily Dosing Regimen

5.3.3.1. Pre-Dose Evaluations

- Symptom-directed physical examination (complete the portion[s] of the physical examination relevant to subject symptoms)
- Vital signs (5-minute sitting BP, pulse, respiratory rate, oral temperature)
- ECOG performance status assessment (Appendix C)
- Clinical laboratory tests (hematology, serum chemistry panel, urinalysis) (see Section 5.6)

- PK and pharmacodynamic blood samples collected within approximately 30 minutes pre-dose (see Section 5.7)
- Concomitant medications
- Adverse events (see Section 7.1.1)

Note: The physical examination and clinical laboratory tests may be performed up to 24 hours before dosing with study drug; test results must be available before study drug administration.

5.3.3.2. Administration of GSK1363089

GSK1363089 should be administered at approximately the same time each day.

After a 2-hour fasting period, subjects will receive GSK1363089. Subjects should drink a full glass of water (approximately 240 cc or 8 oz.) immediately following GSK1363089 administration. Subjects will then continue to fast for 1 hour after study drug administration.

5.3.3.3. Post-Dose Evaluations

- Vital signs (5-minute sitting BP, pulse, respiratory rate, oral temperature) taken within approximately 30 minutes before the PK sampling timepoint
- PK and pharmacodynamic blood samples collected at 4 hours post-dose (see Section 5.7)
- Optional pharmacodynamic samples (eyebrows) [Intermittent 5&9 dosing only]
- Concomitant medications
- Adverse events (see Section 7.1.1)

5.3.4. First Dose of Subsequent 2-Week Treatment Cycles (Study Treatment Period Weeks 3 [Day 15], 5 [Day 29], and 7 [Day 43])

5.3.4.1. Pre-Dose Evaluations

- Physical examination
- Vital signs (5-minute sitting BP, pulse, respiratory rate, oral temperature)
- ECOG performance status assessment (Appendix C)
- Clinical laboratory tests (hematology, serum chemistry panel) (see Section 5.6)
- PK and pharmacodynamic blood samples collected within approximately 30 minutes pre-dose (see Section 5.7)
- 12-Lead ECG (see Section 5.6.6)

- Urinalysis (Week 5 [Day 29] only) (see Section 5.6)
- Pregnancy test (urine or serum) in women of child-bearing potential (Week 5 [Day 29] only)
- Concomitant medications
- Adverse events (see Section 7.1.1)

Note: The physical examination, pregnancy test, and clinical laboratory tests may be performed up to 24 hours before dosing with study drug; test results must be available before study drug administration (no more than 72 hours for the ACTH results) with the exception of thyroid function tests.

5.3.4.2. Administration of GSK1363089

GSK1363089 should be administered at approximately the same time each day.

After a 2-hour fasting period, subjects will receive GSK1363089. Subjects should drink a full glass of water (approximately 240 cc or 8 oz.) immediately following GSK1363089 administration. Subjects will then continue to fast for 1 hour after study drug administration.

5.3.4.3. Post-Dose Evaluations

- Vital signs (5-minute sitting BP, pulse, respiratory rate, oral temperature) taken within approximately 30 minutes before the PK sampling timepoint
- PK and pharmacodynamic blood samples collected at 4 hours post-dose (Week 7 [Day 43] only, for Intermittent 5&9 dosing; Week 3 [Day 15] only, for daily dosing) (see Section 5.7)
- Concomitant medications
- Adverse events (see Section 7.1.1)

5.3.5. Week 7 [Day 47] For Intermittent 5&9 Regimen Only

5.3.5.1. Pre-Dose Evaluations

- Symptom-directed physical examination (complete the portion[s] of the physical examination relevant to subject symptoms)
- Vital signs (5-minute sitting BP, pulse, respiratory rate, oral temperature)
- Clinical laboratory tests (hematology, serum chemistry panel)
- PK and pharmacodynamic blood samples collected within 30 minutes pre-dose
- Concomitant medications
- Adverse events

Note: The physical examination and clinical laboratory tests may be performed up to 24 hours before dosing with study drug; test results must be available before study drug administration with the exception of ACTH which must be available within 72 hours.

5.3.5.2. Administration of GSK1363089

After a 2-hour fasting period, subjects will receive GSK1363089. Subjects should drink a full glass of water (approximately 240 cc or 8 oz.) immediately following GSK1363089 administration. Subjects will then continue to fast for 1 hour after study drug administration.

5.3.5.3. Post-Dose Evaluations

- Vital signs (5-minute sitting BP, pulse, respiratory rate, oral temperature) taken approximately 5 minutes before the PK sampling timepoint (if applicable)
- PK and pharmacodynamic blood samples collected at 4 hours post-dose
- Optional pharmacodynamic samples (eyebrows)
- Concomitant medications
- Adverse events

5.3.6. Week 8

At any time during Week 8, the subject should return to the study site for the following assessments:

- Radiologic tumor assessment by CT-scans or MRI of sites of disease; additional imaging is allowed if clinically indicated (see Section 5.9.3).
- ACTH stimulation test (see Section 5.6.3)
- Thyroid function test (TSH) (see Section 5.6.4)
- Concomitant medications
- Adverse events (see Section 7.1.1)

5.4. Treatment Extension Period

The following evaluations must be performed on the first day of each 2-week treatment cycle during the Treatment Extension Period, unless noted otherwise:

- Physical examination (Day 1 of odd-numbered cycles)
- Symptom-directed physical examination (complete the portion[s] of the physical examination relevant to subject symptoms) (Day 1 of Cycles 6 and 8)
- Vital signs (5-minute sitting BP, pulse, respiratory rate, oral temperature) [Day 1 of each cycle]
- ECOG performance status assessment (Appendix C) (Day 1 of each cycle)
- Clinical laboratory tests (hematology, serum chemistry panel) (Day 1 of each cycle) (see Section 5.6)
- Urinalysis (Day 1 of odd-numbered cycles) (see Section 5.6)
- 12-Lead ECG (Day 1 of odd-numbered cycles) (see Section 5.6.6)
- Pregnancy test (urine or serum) in women of child-bearing potential (Day 1 of odd-numbered cycles)
- ACTH stimulation test (every 8 weeks) (see Section 5.6.3)
- Thyroid function test (TSH) (every 8 weeks) (see Section 5.6.4)
- Ocular assessments – the following examinations must be performed every 6 months (see Section 5.6.5):
    - Best corrected visual acuity
    - Goldman visual field evaluation
    - Measurement of intraocular pressure
    - Slit lamp examination
    - Dilated fundoscopic examination including full field color retinal photographs
- Radiologic tumor assessment by CT-scans or MRI of sites of disease; additional imaging is allowed if clinically indicated (approximately every 8 weeks) (see Section 5.9.3)
- Concomitant medications (Day 1 of each cycle)
- Adverse events (Day 1 of each cycle) (see Section 7.1.1)

Note: The physical examination, clinical laboratory tests, and pregnancy test may be performed up to 24 hours before dosing with study drug; test results must be available before the first dose of the new 2-week treatment cycle (no more than 72 hours for the ACTH results) with the exception of thyroid function tests.

Subjects should continue to fast for 2 hours before and 1 hour after each dose of study drug.

5.5. Post-Treatment Period

5.5.1. End-of-Treatment Visit

Thirty days (±4 days) after the last dose of GSK1363089, subjects are to return to the study site to complete all end-of-treatment assessments as follows:

- Physical examination, including weight
- Vital signs (5-minute sitting BP, pulse, respiratory rate, oral temperature)
- ECOG performance status assessment (Appendix C)
- Clinical laboratory tests (hematology, serum chemistry panel, urinalysis) (see Section 5.6)
- 12-Lead ECG (see Section 5.6.6)
- ACTH stimulation test (see Section 5.6.3)
- Thyroid function test (TSH) (monthly; Daily dosing cohort only) (see Section 5.6.4)
- Ocular assessments - the following examinations must be performed according to the criteria and timelines described below (see Section 5.6.5):
    - Best corrected visual acuity
    - Goldman visual field evaluation
    - Measurement of intraocular pressure
    - Slit lamp examination
    - Dilated fundoscopic examination including full field color retinal photographs

  If study-drug discontinuation was not due to a new or worsening ocular event, the ocular assessments can occur up to and including the 30-day follow-up visit; note that if the most recent exam was within 28 days this repeat exam is not required. However, if the study-drug discontinuation was due to a new or worsening ocular event, the ocular assessments must be conducted as soon as possible (no later than 14 days from onset of the ocular event) and irrespective of whether the most recent exam was within 28 days.
- Pregnancy test (urine or serum) in women of child-bearing potential
- Concomitant medications
- Adverse events (see Section 7.1.1)

5.5.2. Extended Follow-Up Period

On Days 90 and 180 (±15 days) after the last dose of study drug or upon death (if before Day 180), the investigator (or designee) will acquire the following information about subjects who were enrolled and received at least one dose of study drug:

- Subsequent cancer treatments

- Any SAEs considered to be possibly or probably related to GSK1363089, provided that no new treatments have been initiated that would confound the ability to assess the event
- If subject died, the date and cause of death

5.6. Laboratory Assessments

Local laboratories will perform all laboratory tests, and results will be provided to the investigator. Blood and urine samples for hematology, serum chemistry, and urinalysis will be prepared using standard procedures. Laboratory panels are defined as follows:

Hematology

- WBC count with differential
- RBC count
- hemoglobin
- hematocrit
- platelet count

Serum chemistry

- albumin
- ALP
- ALT
- amylase
- AST
- blood urea nitrogen (BUN)
- calcium
- carbon dioxide
- chloride
- creatinine
- GGT
- glucose
- lactate dehydrogenase
- lipase
- phosphorus
- potassium
- sodium
- total bilirubin
- total protein

Urinalysis

- appearance
- color
- pH
- specific gravity
- ketones
- protein
- glucose
- bilirubin
- urobilinogen
- occult blood

Microscopic examination of sediment will be performed only if the results of the urinalysis dipstick evaluation are positive. See also Section 5.6.

Abnormalities in clinical laboratory tests that lead to a change in subject management (e.g., dose reduction or delay, requirement for additional medication or monitoring) are considered clinically significant for the purposes of this study and will be recorded on the AE CRF page. If values meet criteria defining them as serious, they must be reported as SAEs (see Section 7.2.1).

5.6.1. Liver Function Tests

Liver function testing (LFT) will be performed at Screening/Baseline, at specific timepoints throughout the study and at the follow-up visit. LFT should include:

- Alanine aminotransferase (ALT)
- Aspartate aminotransferase (AST)
- Alkaline Phosphatase
- Total Bilirubin

A direct and indirect bilirubin level should be obtained if the total bilirubin level is ≥1.5 x ULN for subjects with normal liver function at baseline or ≥1.5 x baseline for subjects with abnormal liver function at baseline. Liver chemistry threshold stopping criteria and dose modification guidelines have been designed to assure subject safety. Guidelines for interpretation of the liver function tests are described in Section 4.4.4.

5.6.2. Urinalysis

Dipstick urinalysis should include protein, specific gravity, glucose, blood, and leukocyte esterase.

If dipstick confirms protein in the urine, i.e. a reading of ≥ +1, the Urine Protein Creatinine ratio (UPC) should be determined, as described in Appendix E.

Where the UPC ratio is ≥ 3 (i.e. correlates to roughly 3 grams or more of protein excretion in a 24 hour period), a 24-hour urine collection must be performed. Once the 24-h urine collection has been performed and the amount of protein in the urine determined, dose modification decisions should be made according to Section 3.4.

5.6.3. ACTH Stimulation Test

To monitor adrenocortical function at baseline and during treatment, ACTH stimulation tests will be performed.

An ACTH stimulation test will be performed at the following timepoints: screening; during week 8 of the Study Treatment Period; every 8 weeks during the Treatment Extension Period; and at the 30-day follow-up visit. The test should be performed at about the same time of day each time. Results should be available no more than 72 hours before dosing.

A blood sample will be collected to check the pre-test serum cortisol level. Subjects will then receive an intravenous (IV) injection of 250 µg of cosyntropin (ACTH). Approximately 30-90 minutes after the injection of ACTH, a blood sample will be collected to measure the serum cortisol level.

Post-ACTH cortisol levels <20 µg/dL (552 nmol/L) are considered clinically significant; if observed during screening, the subject is ineligible; if observed while on-study, it should be recorded on the AE CRF page.

5.6.4. Thyroid-Stimulating Hormone (TSH) Test

Thyroid function tests will be performed at Screening; during the 8th week of the Study Treatment Period; every 8 weeks during the Treatment Extension Period; and at the 30-day follow-up visit. The test should be performed at about the same time of day each time. At Screening, serum thyroid-stimulating hormone (TSH), Free T3 and T4 will be assessed. Only TSH will be performed for subsequent assessments. Free T3 and T4 will be assessed only if the TSH test is abnormal. Elevated TSH has been observed in studies with other small molecule tyrosine kinase inhibitors of VEGFR.

5.6.5. Ocular Assessments

A complete eye examination by an ophthalmologist is required at baseline (up to 28 days before administration of study drug) and must be repeated every 6 months while on study drug and upon occurrence of new or worsening ocular symptoms (e.g. reduced visual acuity, new-onset night blindness) and discontinuation of study drug. Subjects currently on study must be reconsented and have a complete eye examination within 28 days after acceptance of protocol amendment 4.0. If study-drug discontinuation was not due to a new or worsening ocular event, the eye exam can occur up to and including the 30-day follow-up visit; and if the most recent exam was within 28 days a repeat exam will not be required. However, if the study-drug discontinuation was due to a new or worsening ocular event, the eye exam must be conducted as soon as possible (no later than 14 days from onset of the ocular event) and irrespective of whether the most recent exam was within 28 days.

All testing must be performed independently in each eye.

The following elements must be included in the ophthalmologic evaluation:

1. Best corrected visual acuity

2. Goldman visual field evaluation
3. Measurement of intraocular pressure
4. Slit lamp examination
5. Dilated fundoscopic examination including full field color retinal photographs

Light-dark adaptation testing, International Society for Clinical Electrophysiology of Vision (ISCEV) standard Electroretinography (ERG), and cancer-associated retinopathy antibody testing (e.g. Recombx™ CAR [Anti-Recoverin] Antibody Test, Athena Diagnostics, Inc.) must be performed in subjects who report poor vision in low illumination or have other symptoms suggestive of night blindness prior to initiation of therapy with GSK1363089. These tests can be conducted at any time up to 28 days prior to commencing study drug.

The presence of ocular symptoms or abnormalities on ophthalmologic evaluation at baseline does not preclude a subject from enrolling on the study. Such subjects may enroll on the trial if the treating physician, ophthalmologist and GSK study physician consider it safe to do so. However, it is recommended that counseling be provided to subjects with specific pre-existing ocular conditions before enrollment on the study. The following are suggested recommendations for such counseling:

1. Subjects with evidence of proliferative diabetic retinopathy should be counseled regarding their risks for ocular hemorrhage and vision loss.
2. Subjects found to have a small cup-to-disc ratio on initial ophthalmologic exam should be counseled regarding increased risk of non-arteritic ischemic optic neuritis (NAION). Other risk factors for NAION include atherosclerosis, arterial hypertension, diabetes mellitus, and gastro-intestinal ulcer.

There is no clear association of ocular hemorrhage or NAION with exposure to GSK1363089 at the time of writing Amendment 4.0.

The following are the recommendations for further testing in subjects with specific ocular changes during therapy with GSK1363089:

1. Fluorescein angiography must be performed in subjects with new onset macular edema and in those with vascular occlusion.
2. New-onset visual-field loss must be confirmed by Humphrey 24-2 visual field testing with fovea sensitivity on Swedish Interactive Threshold Algorithm (SITA).

3. Light-dark adaptation testing, ISCEV standard ERG, and cancer-associated retinopathy (CAR) antibody testing (e.g. Recombx™ CAR [Anti-Recoverin] Antibody Test, Athena Diagnostics, Inc.) should be performed for subjects who report poor vision in low illumination or other symptoms suggestive of night blindness..

The GSK study physician should be contacted if new ocular symptoms develop or if there is evidence of worsening of baseline ocular examination findings in any subject on study treatment. Modifications to therapy should be made only after discussion between the treating physician and GSK study physician. Ocular events that do not clearly meet the criteria for an SAE may nevertheless be reported as an SAE, as a medically-important event, after consultation with the GSK study physician.

5.6.6. 12-Lead Electrocardiogram (ECG)

There is no preclinical or clinical evidence of QTc liability with GSK1363089 to date. However, 12- lead ECG monitoring will be conducted in this study as a part of safety monitoring. Subjects will have a 12-lead ECG at Screening, pre-dose on Day 15, Day 29, and Day 43 of the Study Treatment Period; once monthly during the Treatment Extension period; and at the 30-day follow-up visit (see Appendix A).

5.6.6.1. At Screening

If a subject presents with:

- Abnormal ECGs or clinical symptoms, either or both of which are suggestive of a decreased ejection fraction, then he or she is to be evaluated by cardiac imaging (ECHO or MUGA) for determination of cardiac function. The results should be discussed with the GSK Study Physician to decide whether the subject can be enrolled.
- Significant QTc prolongation (defined as a QTc interval > 470 msec), the subject will NOT be eligible for the study.

The subject should take at least 10 minutes of rest before ECG testing. Subjects should be in the semi-recumbent or supine position. The same position must be used for subsequent ECGs. If an ECG demonstrates a prolonged QT interval, two additional ECGs over 30 minutes will be obtained, and the averaged QTc values of the three ECGs will be used to determine whether the subject is eligible for the study.

ECGs must include QTc measurements performed by one of two methods outlined below:

- The machine (method used by the machine must be recorded on the appropriate page of the CRF), or
- Manually, calculated using Bazett's or Frederica's formula given below, and must be recorded on the appropriate page of the CRF.

Bazett's formula is: QTcB = QT/RR0.5

Frederica's formula is: QTcF = QT/RR0.33

5.6.6.2. While on Study Drug

If, while receiving study drug, the subject meets the criteria below upon evaluation of 12-Lead ECG, he/she will be withdrawn from the study.

- QTcB or QTcF > 500 msec (machine or manual overread)
- If subject has bundle branch block then criterion is QTcB or QTcF > 530 msec

The instructions for conducting the ECGs and determining the QTc values are described above in Section 5.6.6.1. Of note, these criteria are based on an average QTc value of triplicate ECGs. If an ECG demonstrates a prolonged QT interval, two additional ECGs over 30 min will be obtained, and the averaged QTc values of the three ECGs will be used to determine whether the subject should be discontinued from the study.

5.7. Pharmacokinetic Assessments

Pharmacokinetic blood samples will be obtained from subjects during the 8-week Study Treatment Period at the following timepoints (Appendix B):

- For Intermittent 5&9 dosing:- pre-dose and at 4 hours post-dose on Days 1, 5, 43, and 47; and pre-dose only on Days 15 and 29
- For daily dosing:- pre-dose and at 4 hours post-dose on Days 1, 8 and 15; and pre-dose only on Days 29 and 43.

Detailed instructions for PK sample preparation and shipping will be provided to the study sites in a separate PK Laboratory Manual. Blood samples for PK assessments will be obtained from all subjects, when possible.

5.8. Pharmacodynamic Assessments - Blood

This section only covers pharmacodynamic blood sampling. See Section 5.9.2. for pharmacodynamic tumor tissue sampling.

Pharmacodynamic blood samples will be collected during the 8-week Study Treatment Period at the following timepoints (Appendix B):

- For Intermittent 5&9 dosing:- pre-dose and at 4 hours post-dose on Days 1, 5, 43, and 47; and pre-dose only on Days 15 and 29
- For daily dosing:- pre-dose and at 4 hours post-dose on Days 1, 8 and 15; and pre-dose only on Days 29 and 43.

Detailed instructions for pharmacodynamic sample preparation and shipping will be provided to the study sites in a separate Pharmacodynamic Laboratory Manual. Blood samples for pharmacodynamic assessments will be obtained from all subjects, when possible.

5.9. Tumor Assessments

5.9.1. Archival Tumor Tissue

Archival or recently-prepared, paraffin-embedded tumor biopsies will be used to analyze *c-MET* gene amplification status. Sites must identify 10 unstained slides of representative tumor tissue or a paraffin block of tumor, collected pre-treatment, and ship them to a GSK-designated central laboratory no later than 14 days after the subject's first dose of study drug.

It is important to note that these paraffin-embedded tumor-tissue samples are to be collected **in addition to** the baseline, fresh, frozen biopsy collected as the first of the paired biopsies described in Section 5.9.2.

In the event that the enrolment of 30 evaluable subjects in Cohort 2 is completed before the target of 4 subjects with *c-MET* amplification (as defined as ≥2 copies of the *c-MET* gene per copy of chromosome 7) has been reached, then extra *c-MET*-amplified subjects (as defined as ≥2 copies of the *c-MET* gene per copy of chromosome 7), identified by pre-screening FISH, will be enrolled in the study, until the target is reached.

In addition to *c-MET* amplification status, the mutational status as well as expression of *c-MET* and related genes and proteins (e.g., RON) and of relevant downstream targets may also be assessed

Gene sequencing and amplification will be determined using standardized assays. The results will be communicated to the enrolling sites by GSK or its designee.

5.9.2. Fresh Frozen Tumor Tissue - Paired Tumor Biopsies

The collection of paired tumor biopsies is required for all subjects who are enrolled in Cohort 2 after the implementation of Protocol Amendment 4.0. These samples will be used to study the effect of GSK1363089 on the expression of pharmacodynamic markers (e.g. total c-MET, phospho-MET and downstream markers).

Detailed instructions for sample preparation will be provided in the Pharmacodynamic Laboratory Manual and kits provided to the sites. The key points are summarized below:

- The baseline tumor biopsy should be collected as soon as possible prior to commencing study treatment, after all other inclusion and exclusion criteria are met, and at least 14 days after the completion of any prior anti-cancer therapy.
- The second tumor biopsy should be collected as close to Day 5 as possible, although it is acceptable to collect it up to Day 8 (Appendix B). It is important that the second tumor biopsy be obtained from the same organ as the pre-treatment biopsy, if at all possible.

Subjects should undergo core biopsies of either the primary gastroesophageal tumor or site of metastasis. The biopsies may be performed percutaneously under imaging guidance, or endoscopically, according to institutional practice. When endoscopic biopsies are performed we recommend at least 5-6 core biopsies with Jumbo forceps to ensure adequate yield of tissue. For the imaging-guided biopsies, 1-2 core biopsies will be performed with a 16-18-gauge needle. We recommend discussion of the feasibility of core biopsies with the radiologist/endoscopist performing the procedure to ensure that core biopsies can be indeed be performed.

Fine needle aspiration must not be used, as it would provide insufficient tumor tissue for studying the effect of GSK1363089 on the expression of pharmacodynamic markers.

All biopsies must be immediately embedded in OCT and snap frozen at -80 °C, in accordance with instructions in the Pharmacodynamic Laboratory Manual. It is highly recommended that a cytopathologist/pathologist be present during the tissue procurement procedures to ensure the presence of cancer cells in the specimen, immediately prior to snap freezing.

Note – these samples **must not** be used to provide paraffin-embedded tissue for determination of the subject's *c-MET*-amplification status. See instead Section 5.9.1.

5.9.3. Tumor Response Assessment

5.9.3.1. Routine Tumor Assessment

The scanning criteria are outlined in Table 4.

Table 4 Tumor Scanning Criteria

Pre-treatment screening period	Study treatment period / Treatment extension period
Within **21 days** of the first dose of GSK1363089:<br>• CT-scans or MRI of chest, abdomen and pelvis<br>• Any additional imaging as clinically indicated	• CT-scans or MRI of sites of disease **every 8 weeks**<br>• Any additional imaging as clinically indicated<br>• Confirmation of response: not less than **28 days after** first documentation of response

Tumor response for subjects with measurable lesions should be assessed routinely (after 8 weeks of treatment and approximately every 8 weeks thereafter) using the RECIST criteria (Therasse et al. 2000); (http://ctep.cancer.gov/guidelines/recist.html or http://imaging.cancer.gov/clinicaltrials/imaging/). Bone lesions, leptomeningeal disease, ascites, pleural and pericardial effusions, lymphangitis cutis, lymphangitis pulmonis, inflammatory breast disease, abdominal masses (not followed by CT or magnetic resonance imaging [MRI]), and cystic lesions should be considered non-measurable. The same assessment method should be used to assess a lesion pre-treatment and post-treatment. See Appendix F for full details.

5.9.3.2. Confirmation of Tumor Response

To be assigned a status of confirmed PR or complete response (CR), changes in tumor measurements must be confirmed by repeated studies that should be performed ≥28 days after the criteria for response are first met. In the case of SD, follow-up measurements must have met the SD criteria at least once after study entry at a minimum interval of 6 weeks. See Appendix F for full details.

5.9.4. Central Pathology Review

Subjects enrolled in this study will require central, independent confirmation of their histologic diagnosis of gastric cancer by a central pathologist (this will occur retrospectively, not prior to enrollment). One or more of the stained slides collected at baseline (see Section 5.6.6) will be provided to the central pathologist.

5.10. Withdrawal Criteria

Subjects may withdraw their consent to participate in the study at any time, without prejudice. The investigator may withdraw a subject if, in his or her clinical judgment, it is in the best interest of the subject, or if the subject cannot comply with the protocol. When possible, the tests and evaluations listed for the termination visit should be carried out. GSK should be notified of all subject withdrawals as soon as possible.

If a subject fails to return for the protocol-defined visits, an effort must be made to determine the reason. If the subject cannot be reached by telephone, a registered letter, at the minimum, should be sent to the subject (or the subject's legal guardian) requesting contact with the clinic. This information should be recorded in the CRF.

The investigator will also withdraw a subject upon GSK's request or if GSK chooses to terminate the study. Upon occurrence of a serious or intolerable AE, the principal investigator (PI) will confer with the GSK representative. If a subject is discontinued due to an AE, the event will be followed until it is resolved. If the AE is not resolved within a reasonable time (approximately 30 days), the event will be followed until its clinical relevance and etiology can be reasonably explained.

5.10.1. Managing Subject Withdrawals

If a subject withdraws from the study at any time either at his or her request or at the PI's discretion, the reason for withdrawal will be recorded in the CRF. All subjects who withdraw from the study prematurely will undergo all end-of-treatment assessments, if possible.

Every effort must be made to undertake protocol-specified safety follow-up procedures.

5.10.2. Replacements

Subjects who do not receive any study drug will be replaced. If a subject is prematurely discontinued from the study for reasons other than GSK1363089-related safety and no outcome measures have been performed, he or she may be replaced.

If, after Amendment 4.0 is implemented, a subject consents to provide paired tumor biopsies, but subsequently is not able to provide the second of the paired biopsies, then that subject will be allowed to continue on study and will be eligible for inclusion in the safety and efficacy assessments. However, if by so doing, this would prevent at least 12 subjects being enrolled who do provide paired biopsies, then an extra subject will be enrolled.

5.11. Warnings and Precautions

In the two Phase 1 studies, GSK1363089 was associated with Grade 3-4 CNS changes, both events occurring in subjects with colorectal cancer and receiving opiates.
One subject with brain metastases developed tumor hemorrhage from a metastatic lesion (see Section 1.3.3.3). Subjects with known brain metastases are excluded from this study. Another subject developed Grade 2 hand-foot syndrome requiring dose reduction.

Many subjects receiving GSK1363089 have developed hypertension. One subject in Study XL880-002, dosed at 120 mg/day of GSK1363089 developed Grade 3 hypertension. This subject had a modification of their antihypertensive regimen during the first two weeks of GSK1363089 dosing, which may have exacerbated the effects of GSK1363089 on hypertension. Since GSK1363089 treatment may result in hypertension, particular caution should be exercised in changing or reducing concomitant antihypertensive medications, particularly agents that may produce BP increases with withdrawal (e.g., beta blockers).

Adverse events considered possibly or probably related to GSK1363089 treatment with a frequency of ≥10% were hypertension or increased BP (42%), fatigue (25%), diarrhea (21%), nausea (18%), increased AST (16%), increased lactate dehydrogenase (16%), vomiting (14%), proteinuria (12%), increased ALT (11%), and blood urine (11%).

No evidence available at the time of the approval of this clinical protocol indicated that special warnings or precautions were appropriate other than those noted above, and/or in Section 5.6.5 relating to ocular events, and/or in the Investigator's Brochure.

6. INVESTIGATIONAL PRODUCT

6.1. Dose and Schedule of Study Drug and Comparator(s)

Chemical Name: Cyclopropane-1,1-dicarboxylic acid {3-fluoro-4-[6-methoxy-7-(3-morpholin-4-yl-propoxy)-quinolin-4-ylamino]phenyl}-amide (4-fluoro-phenyl)- amide, bisphosphate salt

GSK will provide each investigator with adequate supplies of GSK1363089. All study medication capsules will be stored at room temperature and inventoried according to applicable state and federal regulations.

Doses will be administered at the clinical site at each protocol-defined visit during the 8-week treatment period. Subjects should fast 2 hours before study drug administration, drink a full glass of water (approximately 240 cc or 8 oz.) immediately following GSK1363089 administration, and continue fasting through 1 hour after each dose.

Subjects will be provided with sufficient study drug supply and instructions for self-administration. Any unused study drug must be returned to the study site for disposal. The GSK1363089 capsule components are listed in Table 5.

6.2. GSK1363089 Capsules

GSK1363089 will be supplied as 20-, and 100--mg capsules. The GSK1363089 capsule components are listed in Table 5. The percentage compositions of the three GSK1363089 capsule shells are presented in Table 6.

Table 5 GSK1363089 Capsule Components

Ingredient	Function
GSK1363089	Active ingredient
Avicel PH-113 (microcrystalline cellulose)	Filler/binder

Table 6 Composition of GSK1363089 Capsule Shells

20 mg, White		100 mg, Green	
Titanium dioxide	2.9079%	Yellow iron oxide	1.1958%
Gelatin	qsp 100%	Black iron oxide	0.5599%
		Titanium dioxide	1.0602%
		Gelatin	qsp 100%

No comparator will be used in this study.

6.3. Blinding and Randomization

This is an open-label, non-randomized study.

6.4. Compliance

Drug accountability and subject compliance will be assessed using drug dispensing and return records.

6.5. Study Drug Accountability

The investigator will maintain accurate records of receipt of all study drug including dates of receipt. In addition, accurate records will be kept regarding when and how much study drug is dispensed and used by each subject in the study. Reasons for deviation from the expected dispensing regimen must also be recorded. At completion of the study, to satisfy regulatory requirements regarding drug accountability, all unused study medication will be reconciled and destroyed according to applicable state and federal regulations.

7. SAFETY

7.1. Adverse Events and Laboratory Abnormalities

7.1.1. Clinical Adverse Events

An AE is any untoward medical occurrence in a patient or clinical investigation subject administered a pharmaceutical product whether or not it is considered drug related. This would include any side effect, injury, toxicity, sensitivity reaction, abnormal or worsening of a laboratory value, concurrent illness or sudden death. Pre-existing conditions that worsen during a study will be reported as AEs. Toxicity grade will be defined according to the NCI CTCAE v3.0 (http://ctep.cancer.gov/reporting/ctc.html).

The relationship of the AE to the Investigational Drug will be based on the following four definitions, as assessed by the investigator:

1. Not Related
   The AE is ***clearly not related*** to the Investigational Drug because the AE is attributed to an alternate cause(s).

2. Unlikely Related
   The AE is considered ***doubtfully related*** to the Investigational Drug. The AE is not temporally related to the administration of the Investigational Drug, and other factors provide a more plausible explanation of the AE.

3. Possibly Related
   The AE ***may be related*** to the Investigational Drug. The AE is temporally related to the administration of the Investigational Drug, and there are facts to support a causal relationship between the AE and the Investigational Drug; however, other causal explanations for the AE also exist.

4. Probably Related
   The AE is ***likely related*** to the Investigational Drug. The AE is temporally related to the administration of the Investigational Drug and is more likely explained by exposure to the Investigational Drug than by other factors or causes.

7.1.2. Laboratory Test Abnormalities

Laboratory test results will be recorded on the appropriate CRF. Clinically significant laboratory abnormalities, defined as laboratory test results that prompt a change in clinical management (e.g., dose reduction, dose delay, and/or additional medication) will be reported on the CRF AE page. Laboratory data from unscheduled visits that have an associated AE or SAE will be recorded on CRFs and included in the clinical database.

7.2. Handling of Safety Parameters

7.2.1. Serious Adverse Events

Any clinical AE or abnormal laboratory test value that meets the definition of serious noted below and occurs in a subject during the course of the study, irrespective of the treatment received by the subject, must be reported to GSK (or designee) within 24 hours of the investigator becoming aware of the occurrence.

Serious adverse event definition and reporting requirements will be in accordance with the International Conference of Harmonisation (ICH) Guideline for Clinical Safety Data Management, Definitions and Standards for Expedited Reporting, Topic E2A.

An SAE is defined as any untoward medical occurrence that at any dose:

1. Results in death

2. Is immediately life-threatening (i.e., in the opinion of the investigator, the AE places the subject at immediate risk of death; it does not include a reaction that, had it occurred in a more severe form, might have caused death)
3. Requires inpatient hospitalization or results in prolongation of an existing hospitalization
4. Results in persistent or significant disability or incapacity
5. Is a congenital anomaly or birth defect
6. Is an important medical event that may not be immediately life-threatening, result in death, or require hospitalization, but may be considered an SAE when, based upon appropriate medical judgment, it jeopardizes the subject or may require medical or surgical intervention to prevent one of the outcomes listed above.

Any SAE, whether or not considered to be related to treatment with study drug, will be reported to GSK (or designee) within 24 hours of the investigator becoming aware of the event and will be recorded on both the SAE form and the CRF AE page. Additional SAE information including medications or other therapeutic measures used to treat the event, action taken with the study drug due to the event, and the outcome/resolution of the event will be recorded on the SAE form. Forms for reporting SAEs will be provided to the study sites.

Conversely, some hospitalizations, particularly those that are the result of elective or previously scheduled surgery for pre-existing conditions, which have not worsened after initiation of treatment, will not be classified as SAEs. For example, an admission for a previously scheduled ventral hernia repair would not be classified as an SAE; however any complication or untoward event that occurs during the hospitalization or procedure would be collected as an AE. Pre-specified study hospitalizations for observation are not considered SAEs.

All overdoses, whether or not resulting in an AE, require reporting within 24 hours to GSK (or designee).

The investigational site must report all AEs and SAEs that occur through the 30-day end-of-treatment follow-up visit. SAEs that meet the criteria for expedited reporting to the Food and Drug Administration (FDA) will be reported in accordance with US regulations governing safety reporting (Title 21 of the Code of Federal Regulations [CFR] 312.32 and 312.33). Reporting of SAEs by the investigator to his or her Institutional Review Board (IRB) will be done in accordance with the standard operating

procedures and policies of the IRB. Adequate documentation must be maintained showing that the IRB was properly notified.

PPD is the contract research organization (CRO) that has been designated to manage SAE reporting. In case of an SAE, the investigator will be able to contact the PPD SAE Reporting Line or the PPD Safety Medical Monitor at all times:

Telephone number:	1-800-201-8725
SAE Fax number:	1-888-488-9697

7.2.2. Treatment and Follow-Up of Adverse Events

All AEs will be monitored until resolution or, if the AE is determined to be chronic, a cause is identified. If an AE is considered potentially related to study treatment and remains unresolved at the conclusion of the study, the event will be followed until resolution, stabilization, or initiation of treatment that confounds the ability to assess the event. All AEs will be collected at the 30-day follow-up visit.

7.2.3. Follow-Up of Abnormal Laboratory Test Values

In the event of clinically significant abnormal laboratory test values, the tests should be repeated immediately and followed up until they have returned to the normal range or an adequate explanation of the abnormality is found.

7.2.4. Pregnancy

If a subject becomes pregnant during the study, she will be taken off treatment and will be followed through the end of her pregnancy. The outcome of a pregnancy (for a subject or for the partner of a subject) and the medical condition of any resultant offspring must be reported to GSK (or designee). Forms for reporting pregnancies will be provided to the study sites upon request.

8. STATISTICAL METHODS, ANALYSES, AND DETERMINATION OF SAMPLE SIZE

8.1. Statistical and Analytical Plans

Details of the planned analyses will be documented in a separate Statistical Analysis Plan.

8.1.1. Analysis Populations

The safety population will be defined as all subjects who received at least one dose of study drug.

The evaluable population will include subjects in the safety population who:

1. have a baseline and post-baseline tumor assessment, and
2. who have received at least 75% of protocol-mandated doses during the 8-week study treatment period;

as well as subjects who, prior to completing the 8-week Study Treatment Period:

1. require study drug discontinuation due to GSK1363089-related toxicity, or
2. have objective evidence of progressive disease.

All efficacy analyses will be performed on both the safety and the evaluable populations.

All safety analyses will be performed on the safety population.

All efficacy and safety analyses will be conducted on each cohort separately as well as together.

8.1.2. Primary Efficacy Analysis

The primary efficacy variable is the objective response rate (ORR), defined as the proportion of subjects for whom their best overall response is a confirmed CR or confirmed PR by RECIST.

ORR will be summarized using the point estimate of the proportion and an exact 95% confidence interval, for each cohort separately.

If the observed response rate is high enough to warrant an investigation of variables associated with response, exploratory stratified and logistic regression analyses may be performed. Variables that may be considered include age, sex, disease characteristics, performance status, and previous treatment history.

8.1.3. Secondary Efficacy Analyses

Secondary efficacy variables are:

1) Disease stabilization rate (DSR) is defined as the proportion of subjects for whom the best overall response is confirmed PR, confirmed CR, or SD

2) Duration of progression-free survival (PFS) is defined as the time between the date of first dose of study drug and death or disease progression.

3) Duration of Stable Disease is defined as the time between the date of first dose and death or disease progression, in subjects whose best overall response was not PD.

5) Duration of overall survival is defined as time between date of death and date of first dose.

DSR will be summarized using the point estimate of the proportion and an exact 95% confidence interval.

PFS, OS, and duration of stable disease will be summarized using Kaplan-Meier methods. Kaplan-Meier plots and data listings will also be presented.

8.1.4. Safety Analysis

Safety endpoints include adverse events, laboratory data, and concomitant medications.

Adverse event verbatim terms recorded on the CRF will be mapped to preferred terms using the Medical Dictionary for Regulatory Activities (MedDRA).

A treatment emergent adverse event (TEAE) is defined as any adverse event with an onset date on or after date of first dose of study drug, or any ongoing event on date of first dose that worsens in severity after date of first dose. Only TEAE's with an onset date prior to date of last dose + 30 days will be tabulated in the summary tables.

TEAEs will be summarized for overall incidence, worst reported severity, and relationship to study treatment according to system organ class and preferred term.

Laboratory variables will be categorized according to the CTCAE v3.0 and will be summarized using mean change from baseline and shift in category from baseline.

All concomitant medications collected on the CRF's will be mapped to the WHO dictionary. Prior and concomitant medications will be summarized separately by ATC Level and Preferred Term.

8.2. Determination of Sample Size

The trial will accrue 30 evaluable subjects in each Cohort, with or without confirmed c-MET amplification, as defined as ≥3 genomic copies of the *c-MET* gene. After the accrual of each cohort of 30 evaluable subjects, enrollment may be extended (to a maximum of 40 subjects) until at least eight subjects with confirmed c-MET amplification at 7q31, as defined as ≥3 genomic copies of the *c-MET* gene, are enrolled in each cohort. These will be stratified as follows:

- Stratum A: Subjects with confirmed *c-MET* amplification at 7q31.
- Stratum B: Subjects without confirmed *c-MET* amplification at 7q31

Additionally, as part of Amendment 4.0, there will be a requirement in Cohort 2 to have at least 4 evaluable subjects who meet a more stringent definition of *c-MET* gene amplification (i.e. ≥2 copies of *c-MET* per copy of chromosome 7; see Section 3.3.1 for a more detailed definition).

In the event that the enrolment of 30 evaluable subjects in Cohort 2 is completed before the target of 4 subjects with *c-MET* amplification, as defined by ≥2 copies of *c-MET* per copy of chromosome 7, has been reached, then extra subjects, with proven *c-MET* amplification, pre-screened by FISH, will be enrolled in the study, until the target is reached.

This sample size will allow an initial assessment of the tolerability of GSK1363089 in metastatic gastric cancer subjects, when dosed either in the intermittent 5&9 or daily schedules, building on the safety data from the two Phase I studies (77 subjects), which tested each of these schedules in subjects with a variety of solid tumors. Some hypothesis testing will also be possible with regards to the primary efficacy variable, which is the objective response rate (ORR), defined as the proportion of subjects for whom their best overall response is a confirmed CR or confirmed PR by RECIST:

For each Cohort, if at least 6 of the 30 evaluable subjects respond, this regimen of GSK1363089 will have demonstrated a response rate of 20%. This design is used to test the following hypothesis on the ORR:

$$Ho: Po \leq 0.10 \text{ versus } Pa \geq 0.25$$

Assuming a type I error rate (alpha) of 0.10 and a type II error rate (beta) of 0.20, with 30 evaluable subjects and at least 6 observed responses (≥ 0.20) subjects, there is a 0.0732 chance of falsely concluding this regimen is preliminarily effective if the true response rate is 0.10. And the chance for falsely rejecting the effect of the regimen is 0.2026 if the true response rate is 0.25. If additional enrollment is required and at least 8 of the 40 (≥ 0.20) evaluable subjects respond, then the above two probabilities would be 0.0419 and 0.18196, respectively.

With the design of this study, the following table (Table 7) summarizes the expected lower bounds for the 1-sided confidence interval corresponding to the 6/30 or 8/40 scenarios respectively, for each cohort.

Table 7 Lower Bounds for 1-Sided Confidence Intervals

	90% CI	95% CI	97.5% CI
≥ 6 responders of 30	0.10427	0.0759	0.0506
≥ 8 responders of 40	0.1171	0.0925	0.0706

8.3. Pharmacokinetic Analysis

Pharmacokinetic samples will be analyzed using a validated bioanalytical method and will be evaluated using descriptive statistics in conjunction with available safety and pharmacodynamic data.

8.4. Pharmacodynamic Analysis

Pharmacodynamic samples will be analyzed using bioanalytical methods and will be evaluated using descriptive statistics in conjunction with available safety and PK data.

9. DATA QUALITY ASSURANCE

Accurate and reliable data collection will be assured by verification and cross-check of the CRFs against the investigator’s records by the study monitor (source document verification) and by the maintenance of a drug–dispensing log by the investigator. Collected data will be entered into a computer database and subject to quality assurance procedures.

10. STUDY COMMITTEES

The GSK Safety Review Team (SRT) will meet on a monthly basis and will continually assess for subject safety and recommend changes to the protocol and study as required to preserve subject safety and to prevent any untoward toxicities to subjects. The SRT will consist of GSK Physicians from Oncology R&D, Global Clinical Safety & Pharmacovigilance, and Clinical Pharmacology & Discovery Medicine. There is also representation from Biomedical Data Sciences, as well as *ad-hoc* representation from other functional areas within GSK as needed. In addition, all available safety data will be reviewed with study PIs on at least a quarterly basis.

11. ETHICAL ASPECTS

11.1. Local Regulations

The study must fully adhere to the principles outlined in “Guideline for Good Clinical Practice” (GCP) ICH E6 Tripartite Guideline (January 1997). The investigator will ensure that the conduct of the study complies with the basic principles of GCP as outlined in the current version of 21 CFR, subpart D, Part 312, “Responsibilities of Sponsors and Investigators,” Part 50, “Protection of Human Subjects,” and Part 56, “Institutional Review Boards.”

11.2. Informed Consent

It is the responsibility of the investigator, or a person designated by the investigator, to obtain written informed consent from each subject participating in this study after adequate explanation of the aims, methods, anticipated benefits, and potential hazards of the study. In the case where the subject is unable to read, an impartial witness should be present during the entire informed consent discussion. After the subject has orally consented to participation in the trial, the witness’ signature on the form will attest that the information in the consent form was accurately explained and understood.

The CRF for this study contains a section for documenting informed subject consent, and this must be completed appropriately. If new safety information results in significant changes in the risk/benefit assessment, the consent form should be reviewed and updated as necessary. All subjects (including those already being treated) should be informed of the new information, should be given a copy of the revised form, and should give their consent to continue in the study.

Subjects currently on study at the time of acceptance of protocol amendment 4.0 must be reconsented to allow them to have complete eye examinations as described in Section 5.6.5.

11.3. Institutional Review Board

This study is being conducted under a United States Investigational New Drug application. This protocol (and any modifications) and appropriate consent procedures must be reviewed and approved by an IRB. This board must operate in accordance with the current federal regulations. The investigator will send a letter or certificate of IRB approval to GSK (or designee) before subject enrollment and whenever subsequent modifications to the protocol are made.

12. CONDITIONS FOR MODIFYING THE PROTOCOL

Protocol modifications to ongoing studies must be made only after consultation between a GSK representative and the investigator. Protocol modifications will be prepared, reviewed, and approved by GSK representatives.

All protocol modifications must be submitted to the IRB for information and approval in accordance with local requirements, and to regulatory agencies if required. Approval must be obtained before any changes can be implemented, except for changes necessary to eliminate an immediate hazard to study subjects, or when the change involves only logistical or administrative aspects of the trial (e.g., change in monitor, change of telephone number).

13. CONDITIONS FOR TERMINATING THE STUDY

Both GSK and the investigator reserve the right to terminate the study at any time. Should this be necessary, both parties will arrange the procedures on an individual study basis after review and consultation. In terminating the study, GSK and the investigator will assure that adequate consideration is given to the protection of the subjects' interests.

14. STUDY DOCUMENTATION, CRFs, AND RECORD KEEPING

14.1. Investigator's Files and Retention of Documents

The investigator must maintain adequate and accurate records to enable the conduct of the study to be fully documented and the study data to be subsequently verified. These documents should be classified into two separate categories as follows: (1) investigator's study file and (2) subject clinical source documents.

The investigator's study file will contain the protocol and protocol amendments, CRFs, query forms, IRB and governmental approval with correspondence, sample informed consent, drug records, staff curriculum vitae and authorization forms, and other appropriate documents and correspondence.

Subject clinical source documents (usually predefined by the project to record key efficacy and safety parameters independent of the CRFs) include subject hospital/clinic records, physician's and nurse's notes, appointment book, original laboratory reports, ECG, electroencephalogram, X-ray, pathology and special assessment reports, signed informed consent forms, consultant letters, and subject screening and enrollment logs. The investigator must keep these two categories of documents on file for at least 2 years following the marketing application approval date for the study drug and for the indication being investigated or for 2 years after the investigation is discontinued and the FDA is notified. After that period of time, the documents may be destroyed subject to local regulations with prior written permission from GSK. If the investigator wants to assign the study records to another party or move them to another location, GSK must be notified in advance.

If the investigator cannot guarantee the archiving requirement at the study site for any or all of the documents, special arrangements must be made between the investigator and GSK to store these in a sealed container outside of the study site so that they can be

returned sealed to the investigator in case of a regulatory audit. When source documents are required for the continued care of the subject, appropriate copies should be made for storing outside of the study site.

14.2. Source Documents and Background Data

Upon request, the investigator will supply GSK with any required background data from the study documentation or clinic records. This is particularly important when CRFs are illegible or when errors in data transcription are suspected. In case of special problems or governmental queries or requests for audit inspections, it is also necessary to have access to the complete study records, provided that subject confidentiality is protected.

14.3. Audits and Inspections

The investigator should understand that source documents for this study should be made available to appropriately qualified personnel from the GSK Quality Assurance Unit (or designee), or to health authority inspectors after appropriate notification. The verification of the CRF data must be by direct inspection of source documents.

14.4. Case Report Forms

For each subject enrolled, the CRF must be completed and signed by the principal investigator or authorized delegate from the study staff. This also applies to records for those subjects who fail to complete the study. If a subject stops dosing or terminates from the study, the dates and reasons must be noted on the CRF. If a subject terminates from the study because of a DLT, thorough efforts should be made to clearly document the outcome.

All forms should be typed or filled out using indelible ink and must be legible. Errors should be crossed out but not obliterated, the correction inserted, and the change initialed and dated by the investigator or his or her authorized delegate. The investigator should ensure the accuracy, completeness, legibility, and timeliness of the data reported to GSK in the CRF and in all required reports.

15. MONITORING THE STUDY

It is understood that the responsible GSK monitor (or designee) will contact and visit the investigator regularly and will be allowed on request to inspect the various records of the

trial (CRFs and other pertinent data) provided that subject confidentiality is maintained in accordance with local requirements.

It will be the monitor's responsibility to inspect the CRFs at regular intervals throughout the study, to verify the adherence to the protocol and the completeness, consistency, and accuracy of the data being entered on them. The monitor should have access to laboratory test reports and other subject records needed to verify the entries on the CRF. The investigator (or designee) must agree to cooperate with the monitor to ensure that any problems detected in the course of these monitoring visits are resolved.

16. CONFIDENTIALITY OF TRIAL DOCUMENTS AND SUBJECT RECORDS

The investigator must assure that subjects' anonymity will be maintained and that their identities are protected from unauthorized parties. On CRFs or other documents submitted to GSK, subjects should be identified by an identification code and not by their names. The investigator should keep a subject enrollment log showing codes, names, and addresses. The investigator should maintain documents not for submission to GSK (e.g., subjects' written consent forms) in strict confidence.

17. PUBLICATION OF DATA AND PROTECTION OF TRADE SECRETS

The results of this study may be published or presented at scientific meetings. If this is foreseen, the investigator agrees to submit all manuscripts or abstracts to GSK at least 30 days before submission. This allows GSK to protect proprietary information and to provide comments based on information from other studies that may not yet be available to the investigator.

In accordance with standard editorial and ethical practice, GSK will generally support publication of multicenter studies only in their entirety and not as individual study site data. In this case, a coordinating investigator will be designated by mutual agreement.

Any formal publication of the study in which input by GSK personnel exceeded that of conventional monitoring will be considered as a joint publication by the investigator and the appropriate GSK personnel. Authorship will be determined by mutual agreement.

18. REFERENCES

Amemiya H, Kono K, Itakura J, Tang RF, Takahashi A, An F-Q, et al. c-Met expression in gastric cancer with liver metastasis. Oncology 2002;63:286-96.

Amemiya H, Kono K, Mori Y, Takahashi A, Ichihara F, Iizuka H, et al. High frequency of c-Met expression in gastric cancers producing alpha-fetoprotein. Oncology 2000;59:145-51.

American Cancer Society, Inc. Cancer facts and figures 2006. Atlanta: American Cancer Society; 2006.

Cugati S, Cikamatana L, Wang JJ, Kifley A, Liew G, Mitchell P. Five-Year Incidence and Progression of Vascular Retinopathy in Persons Without Diabetes: the Blue Mountains of Eye Study. *Eye*. 2006;20:1239-1245.

Christensen JG, Schreck R, Burrows J, Kuruganti P, Chan E, Le P, et al. A selective small molecule inhibitor of c-Met kinase inhibits c-Met-dependent phenotypes in vitro and exhibits cytoreductive antitumor activity in vivo. Cancer Res. 2003;63:7345-55.

Dicken BJ, Bigam DL, Cass C, Mackey JR, Joy AA, Hamilton SM. Gastric adenocarcinoma: review and considerations for future directions. Ann Surg 2005;241:27-39.

Fuchs CS, Mayer RJ. Gastric carcinoma. N Engl J Med. 1995;333:32–41.

Huang T-J, Wang J-Y, Lin S-R, Lian S-T, Hsieh J-S. Overexpression of the c-Met protooncogene in human gastric carcinoma: correlation to clinical features. Acta Oncologica 2001;40:638-43.

Jhawer M, Kindler, HL, Wainberg Z, et al. Preliminary activity of GSK1363089 (formerly XL880), a Dual c-MET/VEGFR2 Inhibitor, in Poorly Differentiated Gastric Cancer (PDGC): Interim Results of a Multicenter Phase II Study. J Clin Oncol 2008;26: (May 20 suppl; abstr 4572)

Klein RK, Moss SE, Wang Q. Hypertension and Retinopathy, Arteriolar Narrowing and Arteriovenous Nicking in a Population. *Arch Ophthalmol.* 1994;112:92-98

Kuniyasu H, Yasui W, Kitadai Y, Yokozaki H, Ito H, Tahara E. Frequent amplification of the c-Met gene in scirrhous type stomach cancer. Biochem Biophys Res Commun. 1992;189:227-32.

Lal B, Xia S, Abounader R and Laterra J. Targeting the c-Met pathway potentiates glioblastoma responses to gamma-radiation. Clin Cancer Res. 2005;11:4479-86.

Lin W, Kao H-W, Robinson D, Kung H-J, Wu C-W, Chen H-C. Tyrosine kinases and gastric cancer. Oncogene 2000;19:5680-9.

Nakajima M, Sawada H, Yamada Y, Watanabe A, Tatsumi M, Yamashita J, et al. Cancer 1999;85:1894-902.

National Comprehensive Cancer Network. NCCN clinical practice guidelines in oncology: gastric cancer. v.1.2006. [cited 05 October 2006] Available from: http://www.nccn.org/professionals/physician_gls/PDF/gastric.pdf.

Smolen GA, Sordella R, Muir B, et al. Amplification of *MET* may identify a subset of cancers with extreme sensitivity to the selective tyrosine kinase inhibitor PHA-665752. PNAS 2005;103:2316-2321

Sutent Prescribing Information. [cited 1 April, 2008] Available from: http://www.pfizer.com/files/products/uspi_sutent.pdf.

Tahara E. Molecular mechanism of stomach carcinogenesis. J Cancer Res Clin Oncol. 1993;119:265-72.

Therasse P, Arbuck SG, Eisenhauer EA, Wanders J, Kaplan RS, Rubinstein L, et al. New guidelines to evaluate the response to treatment in solid tumors. European Organization for Research and Treatment of Cancer, National Cancer Institute of the United States, National Cancer Institute of Canada. J Natl Cancer Inst. 2000;92:205-16.

Wagner AD, Grothe W, Haerting J, Kleber G, Grothey A, Fleig WE. Chemotherapy in advanced gastric cancer: a systematic review and meta-analysis based on aggregate data. J Clin Oncol 2006;24:2903-9.

Wang J-Y, Hsieh J-S, Chen C-C, Tzou W-S, Cheng T-L, Chen F-M, et al. Alterations of APC, c-met, and p53 genes in tumor tissue and serum of patients with gastric cancer. J Surg Res 2004;120:242-8.

APPENDIX A: STUDY ASSESSMENTS

Study Assessments	Pre-Treatment Period		Study Treatment Period[p] (8 Weeks)							Treatment Extension Period[p]	Post-Treatment Period	
	Screening Within 28 Days of First Dose	Baseline Within 72 Hours of First Dose	Week 1 Day 1	Week 1 Day 5/8[m]	Week 3 Day 15	Week 5 Day 29	Week 7 Day 43	Week 7 Day 47[n]	Week 8	Repeated 2-Week Cycles	30 (±4) Days	90 & 180 (±15) Days
Informed consent	X											
Medical and cancer history	X											
Demographics	X											
Physical examination	X[a]	X[b]			X	X	X			Day 1 of odd cycles	X[a]	
Symptom-directed physical exam			X[a]	X				X		Day 1 cycles 6 & 8		
Vital signs	X	X[b]	X	X	X	X	X	X		Day 1 of each cycle	X	
ECOG performance status	X	X[b,c]	X[c]	X	X	X	X			Day 1 of each cycle	X	
PT/PTT or INR	X											
Hematology, serum chemistry	X	X[b]		X	X	X	X	X		Day 1 of each cycle	X	
Urinalysis	X	X[b]		X		X				Day 1 of odd cycles	X	
12-Lead ECG[d]	X		X		X	X	X			Day 1 of odd cycles	X	
ACTH stimulation test	X								X	every 8 weeks	X	
TSH, Free T3 and T4	X								X[o]	every 8 weeks	X	
Ocular assessments[q]	X									every 6 months	X	
Tumor assessment[e]	X[f]								X	Approx. every 8 weeks		
Pregnancy test[g]	X	X[c]	X[c]			X				Day 1 of odd cycles	X	
Archival tumor tissue for *c-MET* amplification status[h]	X											
Fresh, paired, tumor biopsy tissue[r]	X			X[r]								
Study drug administration[i]			Study drug will be administered on a "5 days on / 9 days off" or a daily dosing schedule.									
PK/PD blood samples[j]			X	X	X	X	X	X				
Concomitant medications	X	X	X	X	X	X	X	X	X	Day 1 of each cycle	X	
Adverse events			X	X	X	X	X	X	X	Day 1 of each cycle	X	
Follow up[l]												X

APPENDIX A: STUDY ASSESSMENTS (CONTINUED)

[a] Physical examination will include measurement of height and weight at screening, weight pre-dose on Day 1, and weight at the 30-day post-treatment visit.

[b] Baseline evaluations should be performed within 72 hours before the first dose. If performed within 24 hours of the first dose, baseline evaluations may serve as the pre-dose Day 1 visit evaluations.

[c] ECOG performance status and pregnancy test may be performed up to 24 hours before study drug administration; test results must be available before study drug administration.

[d] 12-lead ECG will be recorded at screening, at pre-dose on the first day of each 2-week treatment cycle during the Study Treatment Period, once monthly during the Treatment Extension Period, and at the 30-day follow-up visit.

[e] Radiologic documentation must be provided for subjects who are removed from the study for progressive disease.

[f] The baseline radiologic tumor assessment by CT-scans or MRI of chest, abdomen and pelvis may be performed anytime within 21 days before the first dose of GSK1363089. Additional imaging is allowed if clinically indicated. At week 8 and every 8 weeks thereafter, radiologic tumor assessment by CT-scans or MRI of sites of disease; additional imaging is allowed if clinically indicated.

[g] Pregnancy tests will be administered to women of childbearing potential. Results of a screening serum pregnancy test must be available before initial dose of study drug; either serum or urine test may be used at other visits. A positive urine test result must be immediately confirmed by using a serum test.

[h] Tumor tissue will consist of 10 archival or recently-prepared unstained slides or a paraffin block of tumor for shipment to the centralized analysis laboratory no later than 14 days after the subject's first dose of GSK1363089 (or prior to first dose of GSK1363089 if towards study end, only *c-MET*-amplified subjects can be enrolled). See Section 5.9.1 for full details).

[i] In Cohort 1, GSK1363089 will be administered daily on Days 1-5 of each 2-week treatment cycle. In Cohort 2, GSK1363089 will be administered on a daily basis. Study drug will be administered at the study site at each protocol-defined visit during the Study Treatment Period; other doses will be self-administered. Subjects should fast 2 hours before and 1 hour after each dose.

[j] Samples for PK and pharmacodynamic analysis will be collected during the 8-week Study Treatment Period at the following timepoints: in Cohort 2:- pre-dose and at 4 hours post-dose on Days 1, 8 and 15. PK and pharmacodynamic samples will be collected pre-dose only on Days 29 and 43. The pre-dose sample at Week 1 will be considered baseline. See Appendix B for more details.

[k] In Cohort 1, hair plug (eyebrows) may be collected on Day 1 pre-dose, and at 4 hours post-dose on Days 5 and 47. Biopsies of tumor, matched normal tissue, and skin may be collected at pre-dose and within 4-8 hours post-dose on Day 47. In Cohort 2, only tumor biopsies will be collected post-baseline. These are optional, and should be obtained between Days 8 and 15. If a subject remains on study for a prolonged period of time, additional collection of biological samples, including blood, may be performed every 2 months. Detailed instructions for sample preparation will be provided to sites performing these procedures. See Appendix B for more details.

[l] Follow-up will occur 90 and 180 days (±15 days) after the last dose of study drug or until death (if before 180 days); information will be collected on subsequent cancer treatments, any SAEs considered to be possibly or probably related to study drug, and the date and cause of death (if applicable).

[m] These assessments are to be conducted at Day 5 for Cohort 1 and at Day 8 for Cohort 2.

[n] Day-47 assessments are only to be conducted for Cohort 1.

[o] Only TSH will be performed during week 8 and then every 8 weeks. Free T3 and T4 should be assessed only if the TSH test is abnormal

[p] In the absence of toxicity, all scheduled visits should occur within ±3 days of the protocol-specified visit schedule.

[q] The following ocular assessments must be performed in all subjects at baseline (up to 28 days before administration of study drug) and must be repeated every 6 months while on study drug and upon occurrence of new or worsening ocular symptoms (e.g. reduced visual acuity, new-onset night blindness) and discontinuation of study drug: Best corrected visual acuity; Goldman visual field evaluation; Measurement of intraocular pressure; Slit lamp examination; Dilated fundoscopic examination including full field color retinal photographs. See Section 5.6.5 for full details of these assessments plus some extra conditional exams and counseling at baseline.

[r] The baseline tumor biopsy sample will be collected as soon as possible prior to commencing study treatment, but at least 14 days after the completion of any prior anti-cancer therapy (see Section 5.9.2). This sample must **not** be used to provide slides for *c-MET* amplification testing without first consulting the GSK Study Physician. It is highly recommended to collect the second tumor biopsy on Day 5; however, it is also acceptable to collect it as late as Day 8

APPENDIX B: PHARMACOKINETIC AND PHARMACODYNAMIC ASSESSMENTS

Cohort 1 (Intermittent 5&9 Dosing):

	Study Treatment Period					
	**Day 1**	**Day 5**	**Day 15**	**Day 29**	**Day 43**	**Day 47**
**PK and PD Blood Sampling**						
Pre-Dose	X	X	X	X	X	X
4 hours Post-Dose	X	X			X	X
**Optional PD Sampling[a] – Noninvasive samples (eg, hair plug)**						
Pre-Dose	X	X	X	X	X	X
4 hours Post-Dose	X	X			X	X
**Optional PD Sampling[a] – Invasive samples (eg, biopsies of tumor, matched normal tissue, and skin)**						
Pre-Dose	X[b]				X	
4-8 hours Post-Dose		X				X

[a] With the agreement of the investigator and the sponsor and with the subject's consent, other biological samples (e.g. hair plug, tumor biopsy, and normal tissue) may be obtained for pharmacodynamic analyses. During the Treatment Extension Period, optional PD sample collection may occur approximately every 2 months to coincide with tumor assessments. When possible, PK and pharmacodynamic blood samples will be obtained at the same times that biological samples are obtained.

[b] The optional pre-dose Day 1 invasive tissue sample(s) may be collected at any time during the screening period (before first dose of study drug).

Cohort 2 (Daily Dosing):

	Study Treatment Period					
	**Day 1**	**Day 5**	**Day 8**	**Day 15**	**Day 29**	**Day 43**
**PK and Pharmacodynamic Blood Sampling**						
Pre-Dose	X		X	X	X	X
4 hours Post-Dose	X		X	X		
**Pharmacodynamic Tumor Biopsy Sampling[a]**						
Pre-Dose	X[a]	X[a]	X[a]			
4-8 hours Post-Dose		X[a]	X[a]			

[a] Under protocol amendment 4.0, , the collection of repeat tumor biopsy samples for pharmacodynamic analyses is required for all newly enrolled subjects. The baseline tumor biopsy should be collected as soon as possible prior to commencing study treatment, but at least 14 days after the completion of any prior anti-cancer therapy. It is highly recommended to collect the second tumor biopsy on Day 5; however, it is also acceptable to collect it as late as Day 8

APPENDIX C: PERFORMANCE STATUS CRITERIA

**ECOG Performance Status Scale**		**Karnofsky Performance Scale**	
Grade	Descriptions	Percent	Description
0	Normal activity. Fully active, able to carry on all pre-disease performance without restriction.	100	Normal, no complaints, no evidence of disease.
		90	Able to carry on normal activity; minor signs or symptoms of disease.
1	Symptoms, but ambulatory. Restricted in physically strenuous activity, but ambulatory and able to carry out work of a light or sedentary nature (e.g., light housework, office work).	80	Normal activity with effort; some signs or symptoms of disease.
		70	Cares for self, unable to carry on normal activity or to do active work.
2	In bed <50% of the time. Ambulatory and capable of all self-care, but unable to carry out any work activities. Up and about more than 50% of waking hours.	60	Requires occasional assistance, but is able to care for most of his/her needs.
		50	Requires considerable assistance and frequent medical care.
3	In bed >50% of the time. Capable of only limited self-care, confined to bed or chair more than 50% of waking hours.	40	Disabled, requires special care and assistance.
		30	Severely disabled, hospitalization indicated. Death not imminent.
4	100% bedridden. Completely disabled. Cannot carry on any self-care. Totally confined to bed or chair.	20	Very sick, hospitalization indicated. Death not imminent.
		10	Moribund, fatal processes progressing rapidly.
5	Dead	0	Dead

APPENDIX D: CONCOMITANT DRUGS THAT SHOULD BE AVOIDED UNLESS CLINICALLY NECESSARY

Use of drugs that are inhibitors or inducers of CYP3A4 and use of drugs that are substrates of the liver enzymes CYP3A4, CYP2D6, CYP2C8, or CYP2C9 should be avoided in subjects who are receiving GSK1363089 **unless deemed clinically necessary and the subject can be closely monitored for the desired drug effect and potential AEs.** GSK1363089 is highly protein bound, and other drugs that are also highly protein bound should be used with caution (e.g., warfarin, diazepam, furosemide, dicloxacillin, propanolol, and phenytoin). This Appendix provides a list of drugs that inhibit/induce CYP3A4, or are a substrate for CYP3A4, CYP2D6, CYP2C8, or CYP2C9. This list is not entirely comprehensive, and all concomitant medications should be considered for possible interactions with GSK1363089.

**Neuro-psychotropics Antiarrhythmics**<br>• amitriptyline<br>• carbamazepine<br>• clomipramine<br>• clozapine<br>• desipramine<br>• ergotamine<br>• ethosuximide<br>• fluoxetine<br>• haloperidol<br>• imipramine<br>• nortriptyline<br>• paroxetine<br>• perphenazine<br>• risperidone<br>• thioridazine<br>• trimipramine	**Antiarrhythmics**<br>• amiodarone<br>• disopyramide<br>• encainide<br>• flecainide<br>• lidocaine<br>• mexiletine<br>• propafenone<br>• quinidine
**Benzodiazepines**<br>• alprazolam<br>• diazepam<br>• midazolam<br>• phenobarbital<br>• triazolam	**Immunosuppressants**<br>• 17 β-estradiol<br>• budesonide<br>• cortisol<br>• cyclosporine<br>• tacrolimus
**Antihistamines**<br>• astemizole<br>• loratadine<br>• terfenadine	**Calcium channel blockers**<br>• diltiazem<br>• felodipine<br>• nifedipine<br>• nimodipine<br>• nisoldipine<br>• verapamil

**Analgesics**<br>• celecoxib<br>• codeine<br>• dextromethorphan<br>• diclofenac<br>• hydrocodone<br>• ibuprofen<br>• oxycodone<br>• piroxicam	**Angiotensin II receptor blockers**<br>• irbesartan<br>• losartan
**Beta-blockers**<br>• metoprolol<br>• propranolol<br>• timolol	**HMG-CoA reductase inhibitors**<br>• atorvastatin<br>• cerivastatin<br>• lovastatin<br>• simvastatin
**Chemotherapeutics**<br>• cyclophosphamide<br>• ifosfamide<br>• paclitaxel<br>• tamoxifen<br>• vinblastine<br>• vincristine	**Anti-infectious agents**<br>• amprenavir<br>• clarithromycin<br>• clotrimazole<br>• erythromycin<br>• fluconazole<br>• indinavir<br>• itraconazole<br>• ketoconazole<br>• nelfinavir<br>• rifampin<br>• ritonavir<br>• saquinavir<br>• trimethoprim<br>• troleandomycin

**Miscellaneous**
• alfentanil<br>• amodiaquine<br>• bromocriptine<br>• cerivastatin<br>• cimetidine<br>• cisapride<br>• diphenhydramine<br>• estrogen<br>• ethinyl estradiol<br>• fentanyl<br>• fluvastatin<br>• gemfibrozil<br>• grapefruit juice<br>• mibefradil<br>• omeprazole<br>• rosiglitazone<br>• sildenafil<br>• sufentanil<br>• testosterone<br>• tolbutamide<br>• warfarin<br>• zafirlukast

APPENDIX E: URINE PROTEIN CREATININE RATIO (UPC)

Clinical meaning of UPC

There is a good correlation between the ratio of urine protein to creatinine concentrations in a random urine sample and the amount of protein excreted in a 24-hour urine collection period. The creatinine excretion is fairly constant throughout the day regardless of changes in urine flow rate:

Men excrete 20 mg to 25 mg of creatinine/kg of body weight/day

Women excrete 15 mg to 20 mg of creatinine/kg of body weight/day

Normal protein excretion is <100 mg to 150 mg/24 hours.

The UPC ratio is roughly equal to the 24 hour urine protein excretion in g/day.

Calculating Urine Protein to Creatinine ratio (UPC)

UPC ratio = (Urine protein mg/dl) / (urine creatinine mg/dl) = numerically equivalent to gm protein excreted in urine over 24 hrs.

Example: Patient has a urine protein = 90 mg/dl and urine creatinine = 30 mg/dl

UPC ratio = (90 mg/dl) / (30 mg/dl) = 3

Result: UPC is 3 (correlates to roughly 3gm protein excretion in a 24 hour period)

Units for UPC ratio

Note: both the protein concentration and the creatinine concentration must be expressed in the same units (mg/dL or g/L) to perform the UPC calculation. (Units will cancel out; e.g. protein mg /creatinine mg or as protein gm/creatinine gm)

If protein was expressed in mg/dL and creatinine expressed in µmol/L, the UPC ratio would be in mg/µmol, thus, conversion of the units is required, see below:

From	To	Conversion Factor
Conventional Units: mg/dl	SI Units: µmol/l	Multiply by 88.4
SI Units: µmol/l	Conventional Units: mg/dl	Divide by 88.4

Reference:

Xin G, Wang M, Jian L, Xu F, Wang H. Protein-to-creatinine ratio in spot urine samples as a predictor of quantitation of proteinuria. Clinica Chimica Acta 2004;350:35-39

APPENDIX F: THE RESPONSE EVALUATION CRITERIA IN SOLID TUMORS (RECIST) GUIDELINES

Definition of Measurable and Non-measurable Disease

Measurable disease: The presence of at least one measurable lesion.

Measurable lesion: Lesions that can be accurately measured in at least one dimension, with the longest diameter (LD) being:

- ≥20 mm with conventional techniques (medical photograph [skin or oral lesion], palpation, plain X-ray, CT, or MRI),

OR

- ≥10 mm with spiral CT scan.

Non-measurable lesion: All other lesions including lesions too small to be considered measurable (longest diameter <20 mm with conventional techniques or <10 mm with spiral CT scan) including bone lesions, leptomeningeal disease, ascites, pleural or pericardial effusions, lymphangitis cutis/pulmonis, abdominal masses not confirmed and followed by imaging techniques, cystic lesions, or disease documented by indirect evidence only (e.g., by lab values).

Methods of Measurement

The same diagnostic method must be used throughout the study to evaluate a lesion.

Conventional CT and MRI: Minimum sized lesion should be twice the reconstruction interval. The minimum size of a baseline lesion may be 20 mm, provided the images are reconstructed contiguously at a minimum of 10 mm. MRI is acceptable, but when used, lesions must be measured in the same anatomic plane by use of the same imaging sequences on subsequent examinations. Whenever possible, the same scanner should be used.

Spiral CT: Minimum size of a baseline lesion may be 10 mm, provided the images are reconstructed contiguously at 5 mm intervals. This specification applies to the tumors of the chest, abdomen, and pelvis.

Chest X-ray: Lesions on chest X-ray are acceptable as measurable lesions when they are clearly defined and surrounded by aerated lung. However, CT is preferable.

Clinical Examination: Clinically detected lesions will only be considered measurable by RECIST criteria when they are superficial (e.g., skin nodules and palpable lymph nodes). In the case of skin lesions, documentation by color photography - including a ruler and patient study number in the field of view to estimate the size of the lesion - is required.

Baseline Documentation of Target and Non-Target Lesions

- All measurable lesions up to a maximum of five lesions per organ and ten lesions in total, representative of all involved organs, should be identified as ***target lesions*** and recorded and measured at baseline.
- Target lesions should be selected on the basis of their size (lesions with the LD) and their suitability for accurate repeated measurements (either clinically or by imaging techniques).
- A sum of the LD for all target lesions will be calculated and reported as the baseline sum LD. The baseline sum LD will be used as a reference by which to characterize the objective tumor response.
- All other lesions (or sites of disease) should be identified as non-target lesions and should also be recorded at baseline. Measurements of these lesions are not required, but the presence or absence of each should be noted throughout follow-up.
- Documentation of indicator lesion(s) should include date of assessment, description of lesion site, dimensions, and type of diagnostic study used to follow lesion(s).
- All measurements should be taken and recorded in metric notation, using a ruler or callipers.

Response Criteria

Disease assessments are to be performed every 8 weeks after initiating treatment. However, **subjects experiencing a partial or complete response must have a confirmatory disease assessment at least 28 days later.** Assessment should be performed as close to 28 days later (as scheduling allows), <u>but no earlier than 28 days.</u>

Definitions for assessment of response for target lesion(s) are as follows:

<u>Evaluation of Target Lesions</u>

Complete Response (CR) – disappearance of all target lesions.

Partial Response (PR) – at least a 30% decrease in the sum of the LD of target lesions, taking as a reference, the baseline sum LD.

Stable Disease (SD) – neither sufficient shrinkage to qualify for PR nor sufficient increase to qualify for progressive disease (PD), taking as a reference, the smallest sum LD since the treatment started.

Progressive Disease – at least a 20% increase in the sum of the LD of target lesions, taking as a reference, the smallest sum LD recorded since the treatment started or the appearance of one or more new lesions.

Evaluation of Non-Target Lesions

Definitions of the criteria used to determine the objective tumor response for non-target lesions are as follows:

Complete Response – the disappearance of all non-target lesions**.**

Incomplete Response/Stable Disease – the persistence of one or more non-target lesion(s).

Progressive Disease – the appearance of one or more new lesions and/or unequivocal progression of existing non-target lesions.

Evaluation of Overall Response for RECIST-Based Response

The overall response is the best response recorded from the start of the treatment until disease progression/recurrence is documented. In general, the subject's best response assignment will depend on the achievement of both measurement and confirmation criteria.

Table 8 presents the evaluation of best overall response for all possible combinations of tumor responses in target and non-target lesions with or without the appearance of new lesions.

Table 8 Evaluation of Best Overall Response (for RECIST-Based Response Assessment)

Target Lesions	Non-Target Lesions	New Lesions	Overall Response
CR	CR	No	CR
CR	Incomplete response/SD	No	PR
PR	Non-PD	No	PR
SD	Non-PD	No	SD
PD	Any	Yes or No	PD
Any	PD	Yes or No	PD
Any	Any	Yes	PD

Note:

- Subjects with a global deterioration of health status requiring discontinuation of treatment without objective evidence of disease progression at that time should be classified as having "symptomatic deterioration". Every effort should be made to document the objective progression even after discontinuation of treatment.

- In some circumstances, it may be difficult to distinguish residual disease from normal tissue. When the evaluation of complete response depends on this determination, it is recommended that the residual lesion be investigated (fine needle aspirate/biopsy) to confirm the complete response status.

Confirmation Criteria

- To be assigned a status of PR or CR, a confirmatory disease assessment should be performed no less than 28 days after the criteria for response are first met.

- To be assigned a status of SD, follow-up measurements must have met the SD criteria at least once after study entry at a minimum interval of 6 weeks.

APPENDIX G: LIVER CHEMISTRY STOPPING AND FOLLOW-UP CRITERIA

For subjects with normal liver function at Baseline (ALT and bilirubin within normal limits):

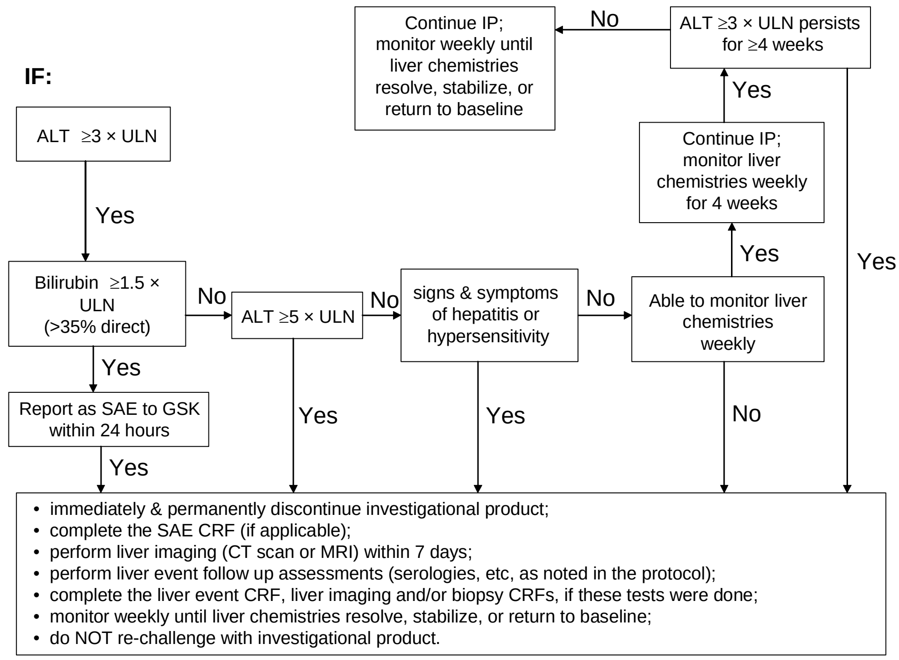

For subjects with abnormal liver function at Baseline (i.e., ALT >ULN or bilirubin >ULN):

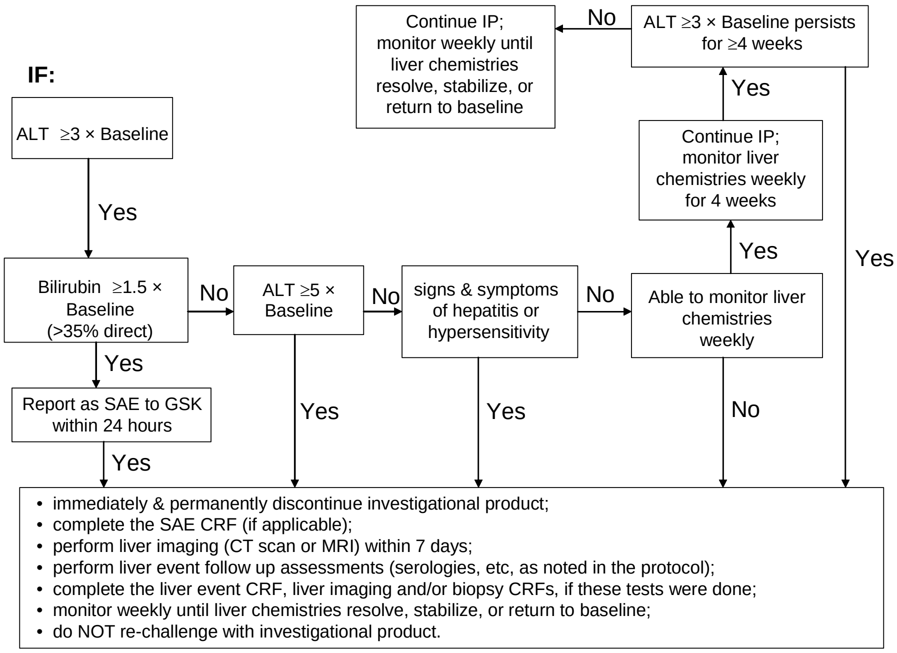

APPENDIX H: SUMMARY OF CHANGES

The two major changes to this study associated with Amendment 4.0 are as follows:

1. Paired tumor biopsy samples, which were optional under Amendment 3.0, have been made mandatory for all newly-enrolled patients, and the timing window for collection of the second tumor biopsy sample has been changed to Day 5-8 from Day 8-15. This is described fully in Sections 3.5 and 5.9.2.
2. Following the observation of a potential ocular safety signal in the GSK1363089 clinical program, an ocular monitoring plan has been introduced in this amendment for ongoing and newly-enrolled subjects, including eye exams to be undertaken during screening and every 6 months while on study drug. Guidance on the interpretation of these assessments, plus possible extra eye exams, and potential changes to the dosing regimen, have also been provided. This is described in Sections 1.3.3.3.4 and 5.6.5.

In addition, a number of other changes have been made to the protocol as part of Amendment 4.0. The key changes are outlined and explained below, in the order in which they appear in the protocol:

3. Section 1.3.3.3.2 – two data errors have been corrected for the daily-dosing Phase I study (XL880-002); namely the number of subjects receiving 80 mg/day has been corrected from 3 to 9, and it has been noted that one of the 2 Grade 4 AEs (elevated GGT) was considered related to GSK1363089.
4. Section 2 – the objectives have been modified to match a change in the study endpoints and analysis plans described in Section 8.1 - two secondary efficacy endpoints have been removed (time to response and duration of response). In addition, the exploratory objectives have been modified to add the option to study the mutational status and expression of genes other than *c-MET* and to add the evaluation of the effect of GSK1363089 treatment on potential pharmacodynamic markers of clinical activity in paired tumor biopsies
5. Section 3.1 – the estimated time of study has been increased from 32 to 40 months, to allow for the slower recruitment rate associated with requiring paired tumor biopsies from all newly-enrolled subjects.
6. Section 3.1.1 – the requirement for archival tumor slides or tissue for FISH analysis of *c-MET* amplification status has been reinstated. In addition, a new requirement

for at least 4 subjects with *c-MET* amplification in Cohort 2, as defined by ≥2 copies of *c-MET* per copy of chromosome 7, has been introduced.

7. Section 3.3.1 – the sample size requirements for *c-MET*-amplified subjects in each cohort are outlined, as well as 2 definitions of *c-MET* amplification.

8. A new section (3.5) has been introduced that describes the requirements for the collection of paired tumor biopsies, both at the subject level and the study level.

9. Section 4.2 (Inclusion Criteria) – two new criteria have been added (the requirement for archival [#3] and fresh [#4] tumor samples). In addition, 2 criteria have been modified:
   - #7 (renumbered as #9) - additional details have been added regarding minimum bilirubin levels for subjects with liver involvement (also in Section 5.6.1)
   - #12 (renumbered as #14) - for QTc intervals, the entry criterion is based on the average of 3 values if the first one is prolonged.

10. Section 4.4.4 – the guidance for the monitoring and treatment of hepatotoxicity has been updated to match language approved by GSK's Global Safety Board.

11. The assessments and procedures for the pre-treatment, treatment and post-treatment periods (Sections 5.2-5.5) have been modified as follows - new ocular assessments have been added; the requirements for archival and fresh tumor samples have been updated; rules for conducting and interpreting ECHO or MUGA after ECG have been added; details of which radiologic tumor assessments to conduct.have been added.

12. Section 5.6.2 and Appendix E – a definition of the urine protein creatinine (UPC) ratio has been added.

13. A new section (5.6.5) has been added, describing the ocular assessments required for ongoing and newly-enrolled subjects.

14. In Section 5.6.6, the instructions around the ECG assessments and when to do ECHO or MUGA have been clarified.

15. Section 5.7.1 has been renumbered as 5.8 and renamed as "Pharmacodynamic Assessments – Blood". Additionally, samples are no longer required for assessment of circulating tumor and endothelial cells.

16. Section 5.7.2 (Potential Supplemental Pharmacodynamic Samples) has been deleted. The instructions for the collection of tumor biopsy samples is described in a new

Section (5.9.2).

17. The Tumor Assessment section has been renumbered as 5.9, and now includes all types of tumor assessment included in the protocol:
    - 5.9.1 – archival tumor tissue for FISH analysis to determine *c-MET*-amplification status.
    - 5.9.2 – fresh-frozen tumor tissue – paired tumor biopsies to study the effect of GSK1363089 on the expression of pharmacodynamic markers.
    - 5.9.3 – tumor response assessments – extra details have been provided around the types of scans and the required body locations. In addition, the time window for the screening-period scan has been reduced from 28 to 21 days. Full details are provided in a new Appendix F.
18. In Section 5.10.2, criteria have been added for replacing subjects who consent to provide paired tumor biopsies, but subsequently are not able to provide the second of the paired biopsies.
19. Section 5.11 (Warnings and Precautions) previously stated - "No evidence available at the time of the approval of this clinical protocol indicated that special warnings or precautions were appropriate other than those noted above, and/or in the Investigator's Brochure". This has been modified with the addition of the bolded text, as follows – "No evidence available at the time of the approval of this clinical protocol indicated that special warnings or precautions were appropriate other than those noted above, **and/or in Section 5.6.5 relating to ocular events,** and/or in the Investigator's Brochure".
20. In Section 6.2 (GSK1363089 Capsules), all mention of the 200-mg capsules has been deleted, as these are no longer used in this study.
21. In Section 8.1, it has been noted that all safety and efficacy analyses will be conducted on each cohort separately as well as together. Additionally, "duration of response" has been deleted from the list of secondary efficacy endpoints.
22. In Section 8.2 (Determination of Sample Size), a new requirement for Cohort 2 is described, whereby at least 4 evaluable subjects who meet a more stringent definition of *c-MET* gene amplification (i.e. $\geq$2 copies of *c-MET* per copy of chromosome 7) must be enrolled.
23. In Section 11.2 (Informed Consent) it is noted that all subjects currently on study at the time of acceptance of protocol amendment 4.0 must be reconsented to allow them to have complete eye examinations as described in Section 5.6.5.

24. The study assessments detailed in Appendix A and Appendix B have been modified to reflect all changes in the procedures & assessments associated with Amendment 4.0.

25. A new Appendix E has been created to define the Urine Protein Creatinine ratio described in Section 5.6.2 (Urinalysis).

26. Detailed RECIST criteria are presented in Appendix F.

27. A schematic representation (flow diagram) of the Liver Chemistry Stopping and Follow-up Criteria described in Section 4.4.4 are presented in Appendix G.

Below is a full copy of Amendment 3.0 of the protocol, with all of the changes that are being instituted as part of Amendment 4.0 highlighted:
