# Supplementary material for: Phase II Study Evaluating 2 Dosing Schedules of Oral Foretinib (GSK1363089), cMET/VEGFR2 Inhibitor, in Patients with Metastatic Gastric Cancer
Source: PLoS One. 2013 Mar 14;8(3):e54014. doi: 10.1371/journal.pone.0054014 (PMC3597709; doi:10.1371/journal.pone.0054014)
Supplement: Appendix S1 — These are the study sites and the approving ethical review boards for participating sites. (DOCX) [file pone.0054014.s004.docx]

Appendix. Study Participation and Ethics Committee Sites

| **Principal Investigator** | **Institution** | **Institutional Review Board** |
| --- | --- | --- |
| Khaldoun Almhanna | Wayne State University  Division of Hematology/Oncology  Karmanos Cancer Institute  4100 John R, Mail Code HW04H0  Detroit, MI 48201 | Wayne State University  Human Investigation Committee  101 E. Alexandrine  Detroit, MI 48201 |
| Jeanne E. Anderson,  MD | Alaska Cancer Research and  Education Center  4001 Laurel Street, Ste. 203  Anchorage, AK 99508 | Western Institutional Review Board  3535 Seventh Ave SW  Olympia, WA 98502-5010 |
| James Burke, MD | Billings Clinic  2825 8th Avenue North  Billings, MT 59101 | Sterling IRB  6300 Powers Ferry Rd.  Suite 600-351  Atlanta, GA 30339 |
| Jeffrey Clark, MD | Massachusetts General Hospital  55 Fruit Street  Boston, MA 02114 | Office for the Protection of Research  Subjects  Dana-Farber Cancer Institute  20 Overland Street, 2nd Floor  Boston, MA 02115 |
| Jerry D. Fain, MD | Texas Oncology, PA  901 West 38th Street Suite 200  Austin, TX 78705 | Sterling IRB  6300 Powers Ferry Rd.  Suite 600-351  Atlanta, GA 30339 |
| James. M. Ford, MD | Stanford University School of  Medicine  Division of Oncology  Center for Clinical Sciences  Research  269 Campus Drive  Stanford, CA 94305-5151 | Medical Human Subjects Panel  Administrative Panels Office  1215 Welch Road, Mod A  Stanford, CA 94305 |
| Charles A. Henderson,  MD | Peachtree Hematology and  Oncology, PC  275 Collier Rd., NW, Suite 400  Atlanta, GA 30309 | Sterling IRB  6300 Powers Ferry Road  Suite 600-351  Atlanta, GA 30339 |
| Howard Hochster, MD | NYU Clinical Cancer Center  160 East 34th Street, 9th Floor  New York, NY 10016 | NYU School of Medicine  Institutional Review Board  550 First Avenue  New York, NY 10016 |
| Jimmy Hwang, MD | Georgetown University Medical  Center  Lombardi Comprehensive Cancer  Center  3800 Reservoir Road, NW  Washington, ND 20007 | MedStar Research Institute –  Georgetown University Oncology  Institutional Review Board  SW104 Medical Dental Building  3900 Reservoir Road, NW  Washington, ND 20057-2197 |
| Yelena Y. Janjigian, MD | Memorial Sloan Kettering Cancer Center  1275 York Avenue, S 1212B  New York, NY 10021 | Institutional Review Board, Memorial Sloan Kettering Cancer  Center  1275 York Avenue, S 1212B  New York, NY 10021 |
| Peter Kennedy, MD | Metropolitan Hematology/Oncology  Medical Group  201 S. Alvarado St., Suite 110  Los Angeles, CA 90057 | Sterling IRB  6300 Powers Ferry Road  Suite 600-351  Atlanta, GA 30339 |
| Hedy Kindler, MD | The University of Chicago Medical  Center  5841 S. Maryland, MC 2115  Chicago, IL 60637-1460 | The University of Chicago  Institutional Review Board  McGiffert Hall, 2nd Floor  5751 S. Woodlawn Avenue  Chicago, IL 60637 |
| Fa-Chyi Lee, MD | University of New Mexico Cancer Center  900 Camino de Salud N.E., MSC 08-4630  Albuquerque, NM 87131-0001 | Western Institutional Review Board  3535 Seventh Ave SW  Olympia, WA 98502-5010 |
| Charles D. Lopez, MD | Department of Medicine, Division of Hematology and Medical Oncology  3181 SW Sam Jackson Park Rd. MC L586  Oregon Health and Science  University  Portland, OR 97239 | Oregon Health and Science University  Institutional Review Board  L-106  3181 SW Sam Jackson Park Rd.  Portland, OR 97239 |
| Barry C. Mirtsching, MD | Center for Oncology Research and  Treatment, PA  7777 Forest Lane, Suite B242  Dallas, TX 75230 | Sterling IRB  6300 Powers Ferry Road  Suite 600-351  Atlanta, GA 30339 |
| James A. Posey, III, MD | University of Alabama at Birmingham  Comprehensive Cancer Center  1824 6th Avenue South  Birmingham, AL 35294-3300 | Western Institutional Review Board  3535 Seventh Avenue SW  Olympia, WA 98502-5010 |
| Ramesh Ramanathan,  MD | TGen Clinical Research Services at Scottsdale Healthcare  10510 N. 92nd Street  Suite 200  Scottsdale, AZ 85258 | Western Institutional Review Board  3535 Seventh Avenue SW  Olympia, WA 98502-5010 |
| William Schelman, MD,  PhD | University of Wisconsin Hospital and Clinics  Clinical Sciences Center, K4/530  600 Highland Avenue  Madison, WI 53792 | Health Sciences Human Subjects  Committee  William S. Middleton VA Hospital  2500 Overlook Terrace Room B3088  Madison, WI 53705 |
| Hope E. Uronis, MD | Seely G. Mudd Bldg., Room #235  10 Bryan Searle Drive  Durham, ND 27710  Duke University Health System | Institutional Review Board (IRB)  Hock Plaza  2424 Erwin Road, Campus Box 2712  Durham, ND 27705 |
| Zev Wainberg, MD | University of California, Los Angeles  10945 Le Conte Avenue, #2338K  Los Angeles, CA 90095 | UCLA Human Subjects Protection  Committee  Office for the Protection of Research  Subjects  11000 Kinross Avenue, Suite 102  Los Angeles, CA 90095 |
