# Supplementary material for: Phase II Study Evaluating 2 Dosing Schedules of Oral Foretinib (GSK1363089), cMET/VEGFR2 Inhibitor, in Patients with Metastatic Gastric Cancer
Source: PLoS One. 2013 Mar 14;8(3):e54014. doi: 10.1371/journal.pone.0054014 (PMC3597709; doi:10.1371/journal.pone.0054014)
Supplement: File S1 — This file contains: Table S1. Drug-related modulation of median plasma HGF, sVEGFR2, sMET and VEGF-A concentrations observed over the first dosing interval in the intermittent 5/9 dosing group. Figure S1. Foretinib inhibits gastric tumor xenograft growth and MET activation in mice xenografts. (A) Mice bearing MKN-45 gastric tumors (n = 10 per group) were treated with vehicle alone (black circles) or foretinib once per day (qd; green arrow) for 21 days at 6 mg/kg (green triangles) or 10 mg/kg (green diamonds), or with 30 mg/kg every other day (q2d; blue arrow) for 42 days (blue circles), or left untreated (black squares). Values represent mean ± SEM of tumor volume obtained from caliper measurements at the indicated days. The log-rank test results were significant (P<0.001) for foretinib at all groups when compared with vehicle control. (B) Mice with MKN-45 tumors measuring 200 to 300 mm3 in size received vehicle, foretinib or pazopanib orally once daily for 3 days at the doses indicated. Tumors were collected at the time points indicated (n = 3 per time point) after last dosing, and phospho-MET (pMET) and total MET (MET) were analyzed by immunoblotting; representative tumor samples are shown. (C) pMET/MET ratios (mean +/− SEM) obtained by quantitative (LI-COR) imaging of tumor samples described in panel B. Filled bars, 4 hours post-dose; clear bars, 24 hours post-dose. Asterisks indicate P<0.01 when compared with vehicle control. (D) Two-site electrochemiluminescent immunoassay analysis of pMET/MET ratio (mean +/− SEM) for tumor samples obtained from animals treated with foretinib 30 mg/kg (black squares) or vehicle (black circles) at the indicated times after dosing. Plasma foretinib concentrations (mean +/− SEM; red triangles) were obtained over the same time course. Figure S2A. Duration of stable disease in the evaluable population (all subjects with best response that was not progressive disease). Figure S2B. Overall survival in the evaluable population. Figure [file pone.0054014.s005.pdf]

Supplemental table 1. Drug-related modulation of median plasma HGF, sVEGFR2, sMET and VEGF-A concentrations observed over the first dosing interval in the intermittent 5/9 dosing group

Marker	Day	n	Median	% change	*P*-value
HGF (pg/mL)	1	42	1018	3.73	NS
	5	36	1056		
sVEGFR2 (ng/mL)	1	42	19.42	-19.78	< 0.0001
	5	36	15.58		
sMET (ng/mL)	1	42	212	14.62	< 0.0001
	5	36	243		
VEGF-A (pg/mL)	1	42	46	193.5	< 0.0001
	5	36	135		

Significant digits of median values reflect the precision of each immunoassay; n indicates number of patients per group; NS: not significant.

Supplemental figure 1. Foretinib inhibits gastric tumor xenograft growth and MET activation in mice xenografts. (A) Mice bearing MKN-45 gastric tumors (n = 10 per group) were treated with vehicle alone (black circles) or foretinib once per day (qd; green arrow) for 21 days at 6 mg/kg (green triangles) or 10 mg/kg (green diamonds), or with 30 mg/kg every other day (q2d; blue arrow) for 42 days (blue circles), or left untreated (black squares). Values represent mean ± SEM of tumor volume obtained from caliper measurements at the indicated days. The log-rank test results were significant ($P < 0.001$) for foretinib at all groups when compared with vehicle control. (B) Mice with MKN-45 tumors measuring 200 to 300 mm^3 in size received vehicle, foretinib or pazopanib orally once daily for 3 days at the doses indicated. Tumors were collected at the time points indicated (n = 3 per time point) after last dosing, and phospho-MET (pMET) and total MET (MET) were analyzed by immunoblotting; representative tumor samples are shown. (C) pMET/MET ratios (mean +/- SEM) obtained by quantitative (LI-COR) imaging of tumor samples described in panel B. Filled bars, 4 hours post-dose; clear bars, 24 hours post-dose. Asterisks indicate $P < 0.01$ when compared with vehicle control. (D) Two-site electrochemiluminescent immunoassay analysis of pMET/MET ratio (mean +/- SEM) for tumor samples obtained from animals treated with foretinib 30 mg/kg (black squares) or vehicle (black circles) at the indicated times after dosing. Plasma foretinib concentrations (mean +/- SEM; red triangles) were obtained over the same time course.

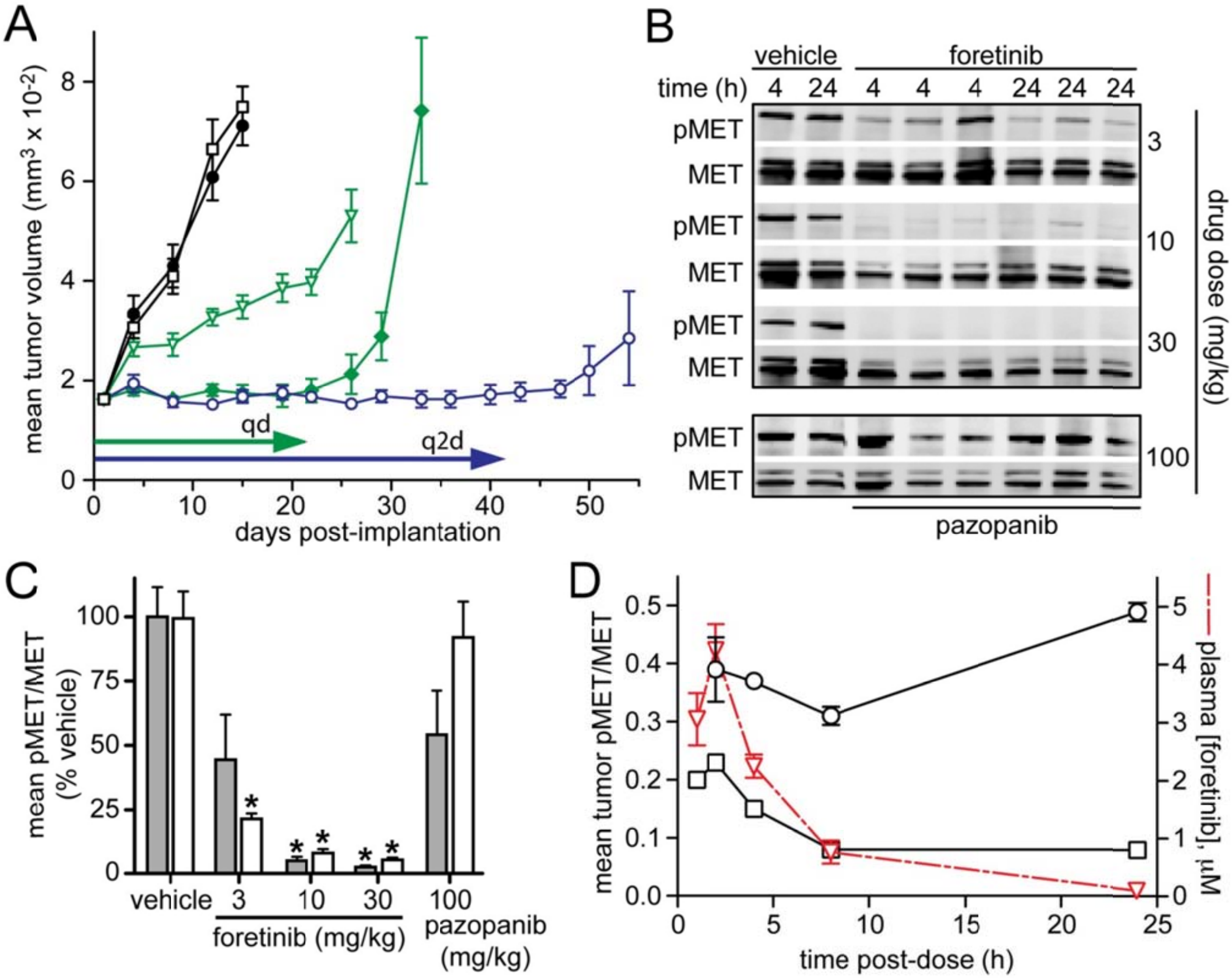
A
mean tumor volume (mm3 x 10-2)
days post-implantation
qd
q2d
B
vehicle
foretinib
time (h)
4 24 4 4 4 24 24 24
pMET
MET
3
10
30
100
drug dose (mg/kg)
pazopanib
C
mean pMET/MET (% vehicle)
vehicle
foretinib (mg/kg)
pazopanib (mg/kg)
D
mean tumor pMET/MET
plasma [foretinib], μM
time post-dose (h)

Supplemental figure 2A. Duration of stable disease in the evaluable population (all subjects with best response that was not progressive disease)

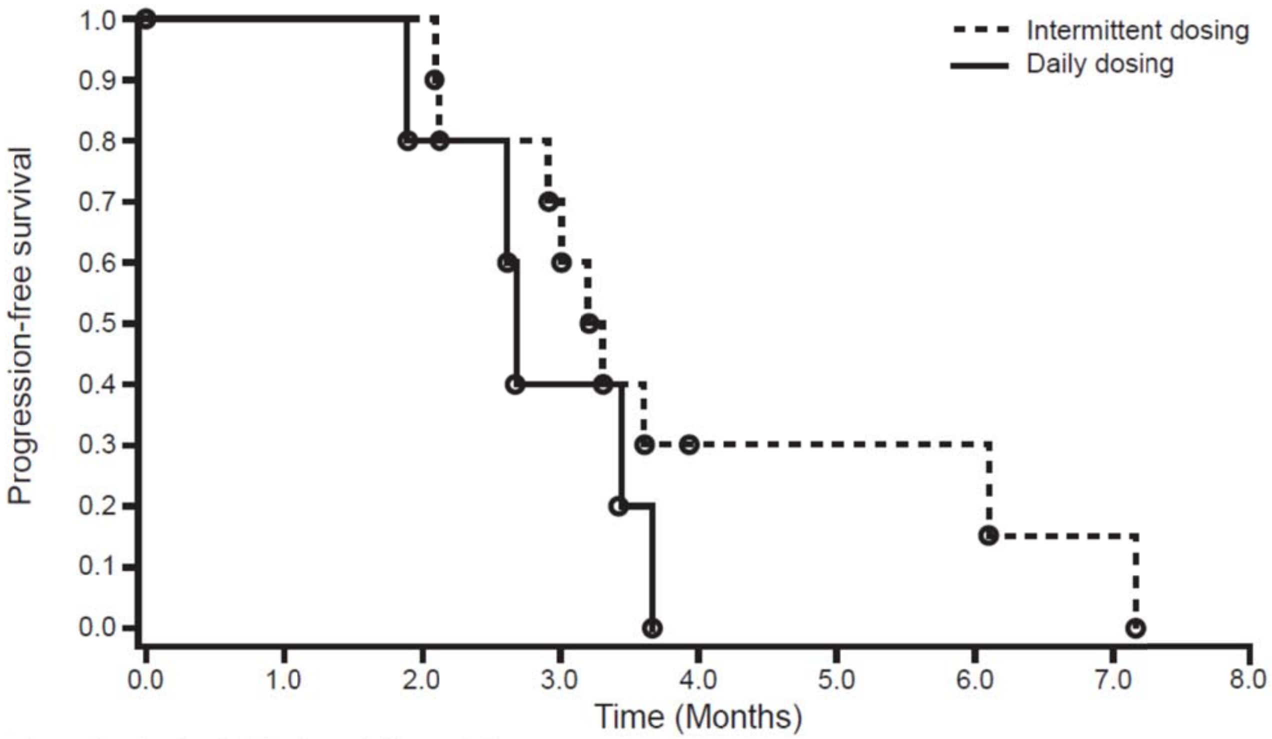

Number of patients at risk at each timepoint

Int Cohort	10	10	10	7	2	2	2	1	0
Daily Dosing	5	5	4	2	0	0	0	0	0

Supplemental figure 2B. Overall survival in the evaluable population

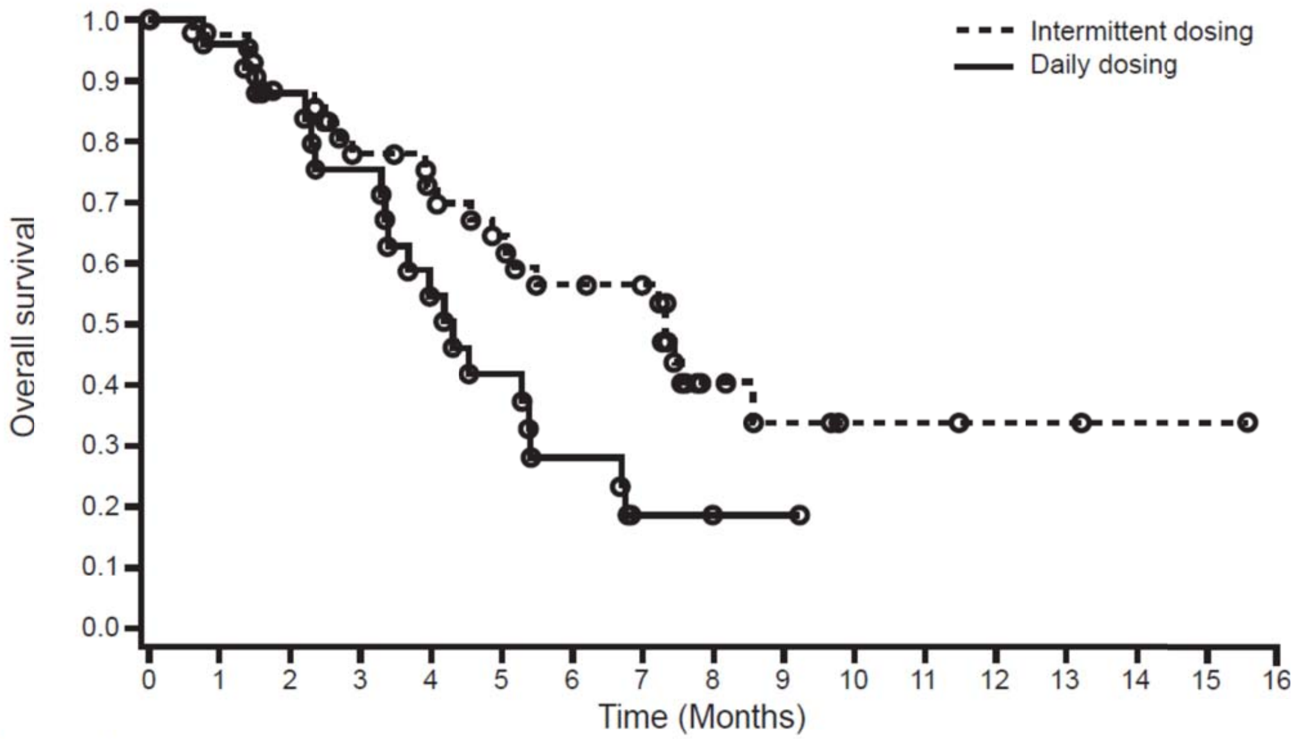

Number of patients at risk at each timepoint

Int Cohort	44	41	35	30	27	24	21	20	7	5	3	3	2	2	1	1	0
Daily Dosing	25	24	21	18	14	9	6	3	2	1	0	0	0	0	0	0	0

Supplemental figure 3. Relationship between trough concentrations on (A) day 5 for the intermittent dosing cohort and percentage change in the sum of the longest diameters (pchange), and on (B) day 15 for the daily dosing cohort and percentage change in the sum of the longest diameters (pchange).

A)

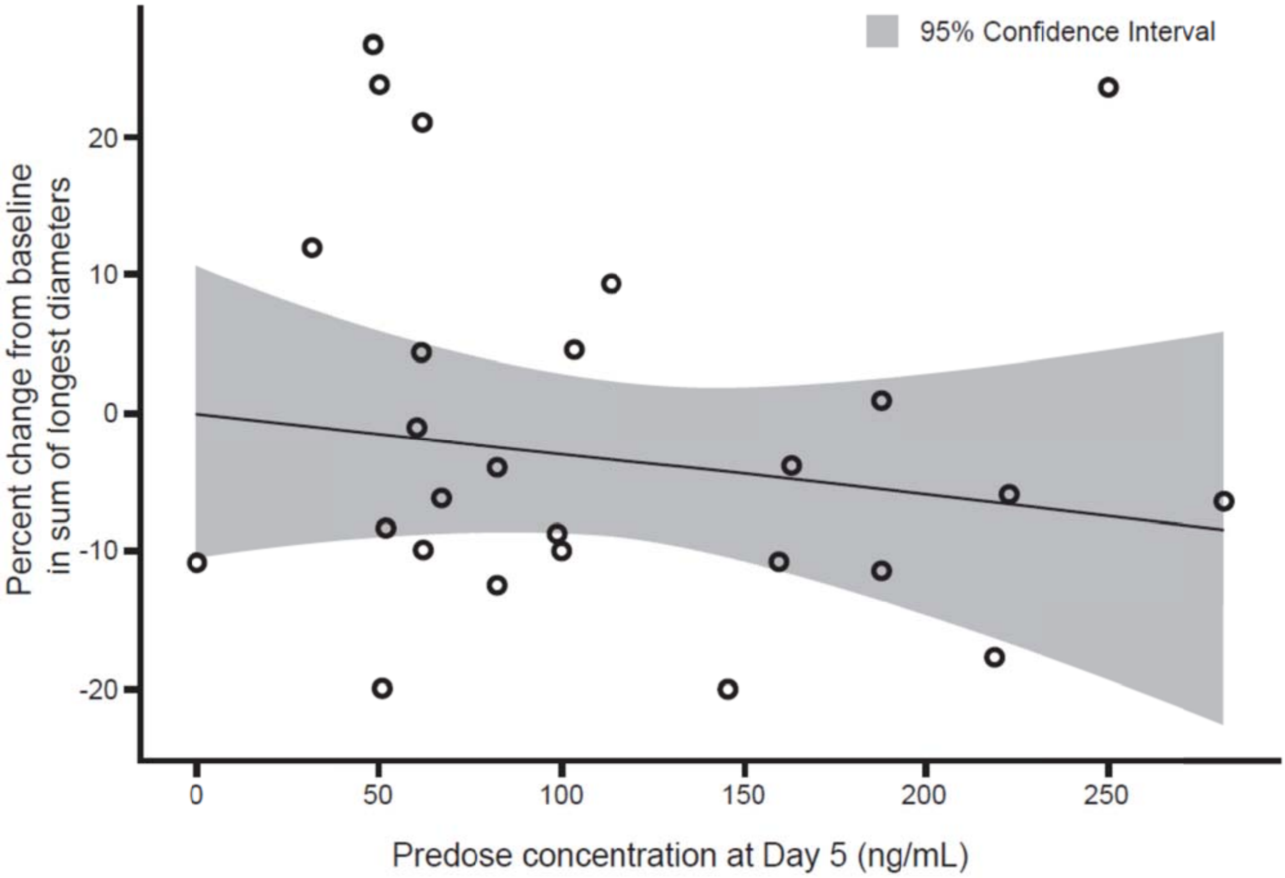

B)

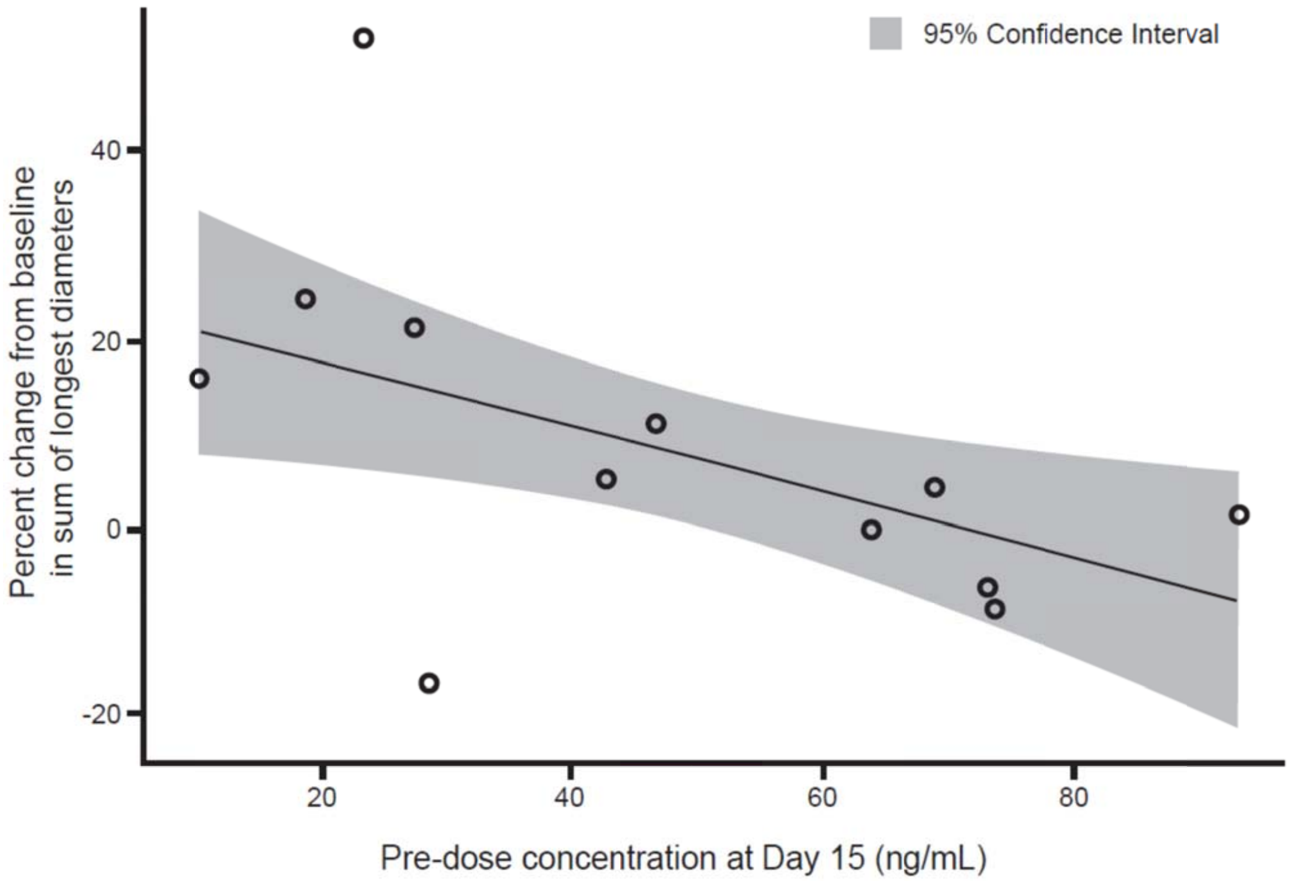
95% Confidence Interval
Percent change from baseline in sum of longest diameters
40
20
0
-20
20
40
60
80
Pre-dose concentration at Day 15 (ng/mL)
